# Collateral sensitivity profiling in drug-resistant *Escherichia coli* identifies natural products suppressing cephalosporin resistance

**DOI:** 10.1038/s41467-023-37624-4

**Published:** 2023-04-08

**Authors:** Dennis Y. Liu, Laura Phillips, Darryl M. Wilson, Kelly M. Fulton, Susan M. Twine, Alex Wong, Roger G. Linington

**Affiliations:** 1 Department of Chemistry, Simon Fraser University, 8888 University Dr., V5A 1S6, Burnaby, BC Canada; 2 Department of Biology, Carleton University, 1125 Colonel By Dr., K1S 5B6 Ottawa, ON Canada; 3 Human Health Therapeutics Research Center, National Research Council Canada, 100 Sussex Dr., K1N 5A2, Ottawa, ON Canada; 4 Institute for Advancing Health Through Agriculture, Texas A&M AgriLife, 1500 Research Parkway, 77845 College Station, TX USA

**Keywords:** Antimicrobial resistance, Natural products, Antibiotics, High-throughput screening

## Abstract

The rapid emergence of antimicrobial resistance presents serious health challenges to the management of infectious diseases, a problem that is further exacerbated by slowing rates of antimicrobial drug discovery in recent years. The phenomenon of collateral sensitivity (CS), whereby resistance to one drug is accompanied by increased sensitivity to another, provides new opportunities to address both these challenges. Here, we present a high-throughput screening platform termed Collateral Sensitivity Profiling (CSP) to map the difference in bioactivity of large chemical libraries across 29 drug-resistant strains of *E. coli*. CSP screening of 80 commercial antimicrobials demonstrated multiple CS interactions. Further screening of a 6195-member natural product library revealed extensive CS relationships in nature. In particular, we report the isolation of known and new analogues of borrelidin A with potent CS activities against cephalosporin-resistant strains. Co-dosing ceftazidime with borrelidin A slows broader cephalosporin resistance with no recognizable resistance to borrelidin A itself.

## Abstract (web summary)

Collateral sensitivity (CS), whereby resistance to one drug is accompanied by increased sensitivity to another, provides new opportunities for antimicrobial drug discovery. Here, Liu et al. screen large chemical libraries across 29 drug-resistant *E. coli* strains to identify multiple CS interactions, including natural products with potent CS activities against cephalosporin-resistant strains.

## Introduction

The rise of antimicrobial drug resistance (AMR) worldwide represents one of the most pressing contemporary threats to human health^1,2^. In the most comprehensive study to date, Murray et al. reported an estimated 4.95 million deaths associated with bacterial AMR globally in 2019 alone^3^. Chief among the list of AMR culprits are pathogenic strains of *Escherichia coli*, including third-generation cephalosporin-resistant and fluoroquinolone-resistant strains, which in total account for more than 800,000 associated deaths^3^. AMR is challenging to address because resistance is an inherent consequence of antimicrobial drug use; even a single long-term infection may result in the emergence of drug resistance^4^. The ubiquitous and non-compliant overuse of antimicrobials in both clinical and agricultural settings contribute to large scale resistance evolution^2^. Despite recent attention to AMR, the incidence of multidrug resistant (MDR) infections continues to rise^5^ and resistance to newer drugs is often observed within a few years of deployment^6^. In contrast, the rate of antimicrobial drug discovery has declined considerably owing to low profitability and shifting priorities within the pharmaceutical industry^7,8^, further exacerbating the AMR crisis.

One approach to treating AMR strains is to exploit orthogonal sensitivities to one drug that arise from resistance to another drug, a phenomenon known as collateral sensitivity (CS)^9–11^. CS interactions have been widely observed in both drug-resistant microbes and in cancer^12^, and are thought to result from trade-offs inherent to the maintenance of pleiotropic drug resistance mutations^9^. Using laboratory-evolved drug-resistant *E. coli* strains, several studies have mapped out CS relationships between known antibiotics and have demonstrated their application against clinical isolates^9^. These include the deployment of two reciprocally CS-active drugs in an alternating drug-cycling regimen, such as cefuroxime and gentamicin in *E. coli*^10^, or the use of a primary drug with its CS-active adjuvant in combination. For example, using mecillinam with cefotaxime has been shown to constrain the evolution of a globally distributed extended spectrum β-lactamase^13^. Most bacterial CS studies to date, however, have been limited to known, commercially available antimicrobials^14^. Few studies have examined the CS potential of natural products (NPs) from an antimicrobial perspective^11,15,16^, despite the obvious value of NP chemical diversity and biological relevance in antibiotic therapy development.

In the context of drug resistance, both known and novel NPs have the potential for CS activity. This provides an opportunity to re-examine the chemical space occupied by known NPs, which may contribute to the discovery and design of new CS-based antimicrobials. Furthermore, CS-active compounds can improve understanding of complex CS mechanisms, as well as provide additional insight into the associated mechanisms of resistance^9,17^.

This work presents a high-throughput screening platform, based on a panel of 29 isogenic drug-resistant mutants of *E. coli*, to map and identify natural products that elicit CS across diverse drug-resistant phenotypes, termed Collateral Sensitivity Profiling (CSP). We first validate this approach using a collection of commercial antimicrobials covering 30 drug classes, revealing both new and previously reported CS relationships. Extrapolating this hypothesis into novel chemical space, we report the screening of a library of 6195 marine Actinobacterial and *Burkholderia* natural product extract prefractions and the observation of widespread CS interactions in nature. From this screen, we describe the isolation and characterization of the borrelidin family of macrolide NPs, including a new analogue borrelidin P, with specific CS activity against cephalosporin-resistant *E. coli* but not wildtype (WT). To our knowledge, this is the first instance of a new NP compound discovered on the basis of its CS activity. Follow-up experiments reveal that threonine—tRNA ligase (ThrRS), the known target of the borrelidins, contributes to the observed CS interaction with *E. coli* strains bearing cell wall biosynthesis mutations. Further investigation suggests that other targets of the borrelidins may also play a role in its CS activity. Finally, we show that co-dosing ceftazidime with borrelidin A slows the emergence of cephalosporin resistance and suppresses existing ceftazidime resistance below key thresholds.

## Results

### Development of a high-throughput collateral sensitivity screen in *Escherichia coli*

To identify CS interactions, a panel of 29 drug-resistant strains covering multiple drug classes was derived from the standard lab model *E. coli* MG1655 (K-12). Twenty-six of these strains carry chromosomal resistance mutations and were isolated as spontaneous mutants on various antibiotic-containing agar plates, while an additional three strains were transformed using multidrug-resistant (MDR) plasmids from previously characterized clinical isolates^18,19^. Each mutant was sequenced to determine the genetic basis of its resistance, relevance to the clinical literature, and to ensure that it harbored only a single mutant gene, where applicable (Supplementary Table 1). This approach allows unbiased comparisons between WT and drug-resistant strains due to a uniform genetic background and thus provides a fundamental perspective of CS in any chemical library. In brief, the target panel is comprised of the following: 7 quinolone-resistant strains with mutations in either DNA gyrase subunit A (*gyrA*) or subunit B (*gyrB*); 4 chromosomal multidrug-resistant (MDR) strains with mutations in one of two multidrug efflux pump pathways (*marR* or *acrR*); 3 rifamycin-resistant strains with mutations in bacterial RNA polymerase subunit β (*rpoB*); 4 cephalosporin-resistant strains with mutations in either cell envelope biosynthesis (*envZ*) or lipopolysaccharide biosynthesis (*rfaH, rfaG*); and 8 aminoglycoside-resistant strains with mutations in the cytochrome o oxidation pathway (*cyoA*), ubiquinone biosynthesis genes (*ubiB, ubiF*), or the 30S ribosomal protein S12 (*rpsL*). The 3 plasmid-borne MDR strains contain vectors from drug-resistant *E. coli* (pAC29, pAC30) or *K. aerogenes* (RK2). Together, the target panel covers a broad range of resistance mechanisms, including DNA replication, RNA synthesis, protein synthesis, cell wall biosynthesis, MDR, and plasmid-borne resistance. Approximately 60% of these strains contain mutations that have been previously reported in drug-resistant pathogens, with varying levels of clinical relevance^20–26^.

We emphasized single, chromosomal mutants in this target panel as opposed to more clinically relevant mobile resistance cassettes for three main reasons. Firstly, the focus of this study was to explore the prevalence of CS in nature using a first-principles approach; target panel selection prioritizing diversity in drug resistance mechanisms was better aligned with this goal compared to exclusive representation by clinically relevant plasmid-borne β-lactamases. CSP therefore aims to identify a broad range of CS responses to provide broad biological target coverage for large libraries containing diverse chemistries. Secondly, CS screening of complex NP extracts necessitated a simple, minimally-variable genetic background due to the potential for interference. Multi-gene mutants and plasmid-borne resistance vectors contain several points of variability, which together may produce synergistic and/or antagonistic interactions that complicate comparison with the WT strain. Instead, we selected only single gene mutants that would unambiguously connect any observed CS interaction to a specific drug resistance mutation and compound/extract. Thirdly, chromosomal resistance mutations are more genetically stable and therefore more amenable to high-throughput screening. In contrast, plasmid-borne strains may require both retention control (selection antibiotic) and copy control (inducer) to standardize expression, significantly complicating CS screening.

**Fig. 1:**
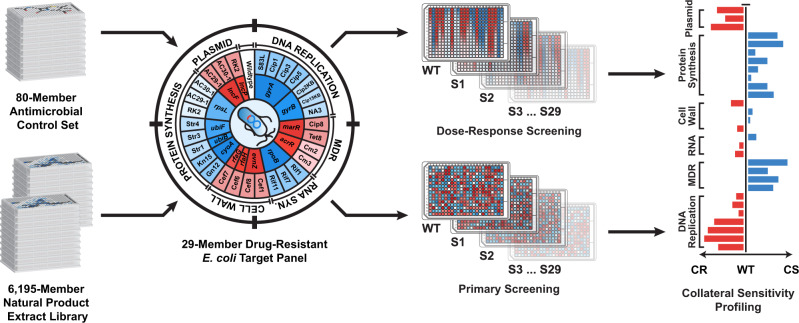
Schematic diagram of collateral sensitivity profiling (CSP). Eighty antimicrobials covering a broad range of drug classes (control set for assay optimization) and an in-house library of 6195 natural product extract prefractions served as the primary source of chemical diversity for this study. Both the antimicrobial control set and the natural product library were systematically screened against wildtype *E. coli* (WT) and 29 drug-resistant strains (S1–S29) as either dilution series (antimicrobials) or at a single stock concentration (natural product prefractions); growth monitored via OD_600_ readings. Resulting bioactivity data for each antimicrobial and extract were normalized to wildtype values and used to construct a collateral sensitivity profile. CR cross resistance; drug-resistant strain(s) exhibiting decreased sensitivity to the drug/extract compared to wildtype (red). CS collateral sensitivity; drug-resistant strain(s) exhibiting increased sensitivity to the drug/extract compared to wildtype (blue). Diverse mechanisms of resistance across the target panel provides insight into the mechanism of collateral sensitivity: MDR multidrug resistance, RNA RNA synthesis, Cell Wall cell wall biosynthesis, Plasmid plasmid-borne multidrug resistance.

For CSP, we developed a fully automated 384-well microtiter assay protocol that systematically tests each chemical sample against all 30 test strains (WT + 29 drug-resistant strains; Fig. 1). Each strain was dispensed into clear microtiter plates at a standardized inoculum before treatment with test compounds (either as dilution series for antimicrobial agents or at a single stock concentration for natural product extracts). Compound-treated plates were incubated overnight, and cell growth assessed via OD_600_ absorbance. The activity of a given test compound/extract against each of the 29 drug-resistant strains was then used to construct an activity ‘fingerprint’ by standardizing to the activity of the WT strain. Loss of activity in one or more mutant strains compared to the WT signified resistance or cross resistance (CR). Conversely, an increase in activity in one or more mutant strains compared to WT inferred a CS interaction. CSP thus provides a clear and comprehensive examination of the CS properties of any compound library in high throughput.

### Profiling of commercial antimicrobials identifies both known and novel CS relationships

**Fig. 2:**
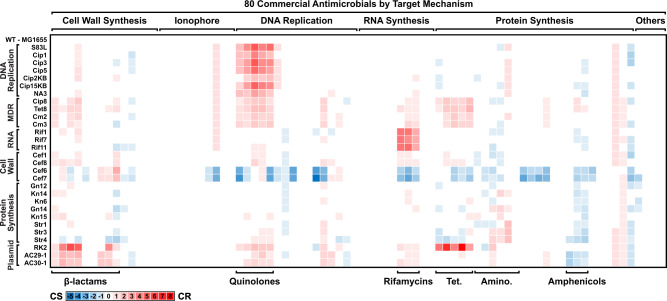
Collateral sensitivity profiling (CSP) of 80 commercial antimicrobials against a drug-resistant *E*. *coli* target panel. Legend: white = no change in Log_2_MIC compared to wildtype; red = increase in Log_2_MIC compared to wildtype (resistance); blue = decrease in Log_2_MIC compared to wildtype (collateral sensitivity). Antimicrobials are categorized along the horizontal axis by their mechanism of action (top) and specifically by their drug classes (bottom). Mutant *E. coli* strains are categorized by their resistance mechanism (left). MDR multidrug resistance, RNA RNA synthesis, Cell Wall cell wall biosynthesis, Plasmid plasmid-borne multidrug resistance, Tet. tetracyclines, Amino. aminoglycosides. Data shows the average of three independent experiments (*n* = 3). For a fully annotated version of this figure see Supplementary Information Fig. 1. Source data are provided as a Source Data file.

Eighty commercially available antimicrobials covering 30 compound classes (Supplementary Table 2) were screened in CSP to evaluate the prevalence of resistance-mediated CS to known drugs. Each antimicrobial was serially diluted (16 two-fold dilutions from 128 μM to ~4 nM) and screened in biological triplicate against the full target panel plus WT (average Z’-factor = 0.71, Supplementary Table 3). Minimum inhibitory concentration (MIC) values for each drug-strain combination were used to calculate fold-change differences (Log_2_MIC_Mutant_ – Log_2_MIC_WT_) between WT and drug-resistant strains (Fig. 2; for a fully annotated table with quantified values, see Supplementary Fig. 1). In total, 34 out of 80 antimicrobial compounds induced at least a 2-fold increase in resistance to one or more drug-resistant strains, with the largest difference corresponding to an upwards 256-fold shift in the MIC. As anticipated, resistance and CR among mutant strains were observed for their original selection antibiotic and same-class analogues, respectively. Both chromosomal and plasmid-borne MDR strains exhibited widespread CR patterns across the entire antimicrobial set, notably against β-lactams, fluoroquinolones, rifamycins, and tetracyclines. Conversely, 43 antimicrobial compounds presented a minimum of 2-fold increase in CS activity to one or more strains; the largest being a downwards 32-fold shift in the MIC. CS activity was generally congruent among same-class analogues. The cephalosporin-resistant cell wall biosynthesis mutants, Cef6 and Cef7, displayed increased sensitivity towards a broad range of antimicrobials, including select fluoroquinolones, rifamycins, select aminoglycosides, macrolides, amphenicols, anthracyclines, and lipopeptides. This finding is consistent with previous studies that have identified a correlation between the frequency of CS interactions and cell wall mutations^11^. Furthermore, amphenicol antimicrobials presented broad and consistent CS activity in strains with rifamycin and aminoglycoside resistance, as well as plasmid-borne MDR. The CS relationship between chloramphenicol and aminoglycoside-resistant *E. coli* has been previously reported by Imamovic, et al.^10^. Here, we show that chloramphenicol’s CS activity can be extrapolated to other amphenicols, which together are also CS-active against select cephalosporin-, fluoroquinolone-, and MDR strains in addition to aminoglycoside-resistant strains. A recent study by Li et al. demonstrated the utility of pleuromutilin antibiotics in treating vancomycin-resistant *Enterococcus faecium*^17^. Analogously, CS profiling has shown that the pleuromutilin antibiotic tiamulin presents potent CS activity across the spectrum of drug-resistant *E. coli* strains. Finally, certain antimicrobials, such as polymyxin B and novobiocin, possessed unique CS activity profiles not shared by their analogues.

### Collateral sensitivity profiling of natural product extracts reveals widespread CS relationships in nature

**Fig. 3:**
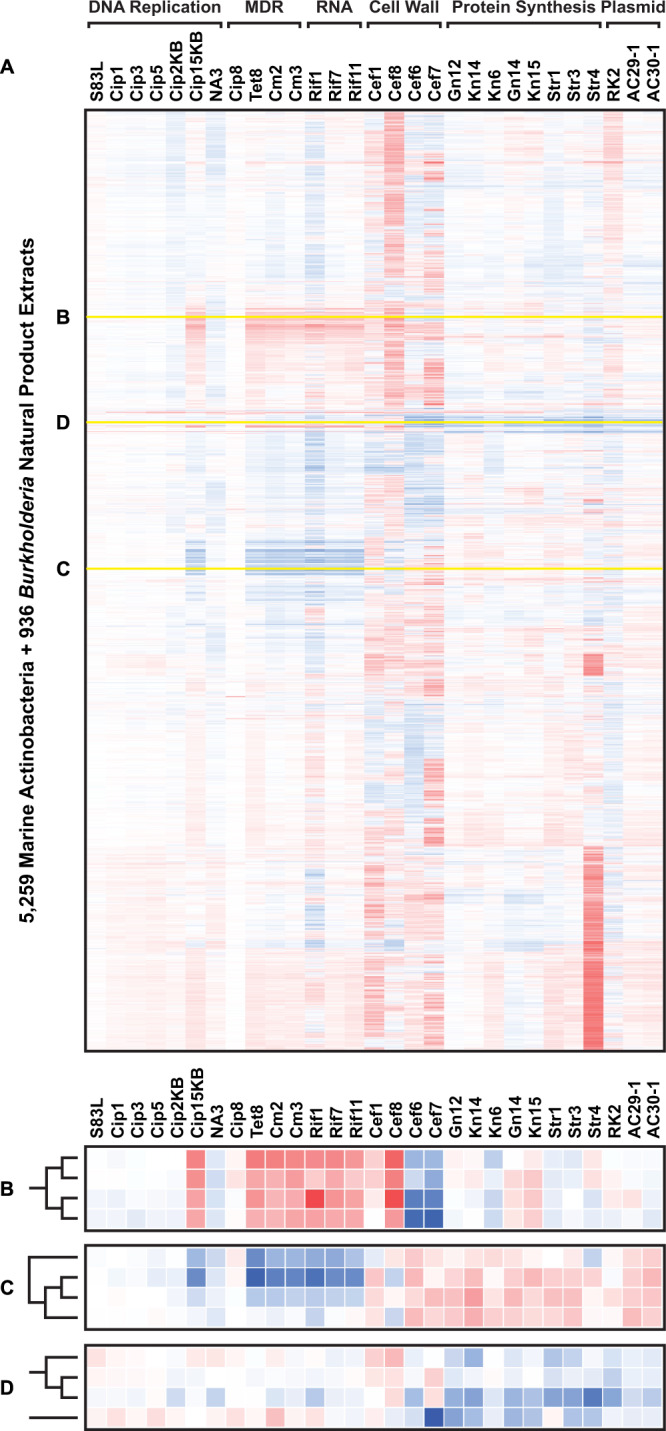
Collateral sensitivity profiling of 6195 natural product extracts. **A** Hierarchical clustering of 5259 marine-derived Actinobacterial and 936 *Burkholderia* natural product extract prefractions (vertical axis) profiled against wildtype *E. coli* and 29 drug-resistant strains (horizontal axis. MDR multidrug resistance, RNA RNA synthesis, Cell Wall cell wall biosynthesis, Plasmid plasmid-borne multidrug resistance). Yellow highlighted lines denote insets **B**, **C**, and **D** with dendrograms. **B** Zoomed inset showing NP cluster producing CR in multidrug-, rifamycin-, cephalosporin-, and fluoroquinolone-resistant strains. **C** Zoomed inset showing NP cluster producing CS in multidrug-, rifamycin-, and fluoroquinolone-resistant strains. **D** Zoomed inset showing NP cluster with CS activity in aminoglycoside- and plasmid-borne multidrug-resistant strains. Legend: white = no observable change in percent growth compared to wildtype; red = increase in percent growth compared to wildtype (resistance); blue = decrease in percent growth compared to wildtype (collateral sensitivity). Source data are provided as a Source Data file.

We next sought to explore the prevalence of CS within the chemically-diverse and bioactivity-rich domain of natural products. Natural products have been widely studied in the past as a privileged source of antimicrobial scaffolds^27^. Our group maintains an in-house library of natural product extract prefractions derived from diverse marine Actinobacterial and terrestrial *Burkholderia* microorganisms. To investigate the CS potential of this NP library, the entire collection (6195 prefractions) was systematically screened using CSP (185,850 combinations; average Z-factor = 0.55; Supplementary Table 3). Resulting growth data (OD_600_ absorbance values) for each extract-strain combination were normalized by strain and expressed as a percent difference from the wildtype standard (%Growth_Mutant_ – %Growth_WT_), then processed through hierarchical clustering to group extracts by their CS phenotype (Fig. 3A). A cut-off of one standard deviation was established to select hits from the primary screen. We rationalized that the lack of additional replicates (due to limited extract material) justified an inclusive primary hit selection approach, given that this was complemented by a comprehensive secondary screen for hit validation.

Extensive patterns of both CR and CS were observed in this NP dataset. Out of 6195 total, 631 extracts (617 Actinobacteria and 14 *Burkholderia*) displayed CR relationships in at least one drug-resistant strain. A significant portion of the NP library elicited strong resistance from the aminoglycoside-resistant Str4 mutant, while a distinct cluster of extracts experienced CR from several MDR strains (Tet8, Cm2, Cm3), the rifamycin-resistant strains (Rif1, Rif7, Rif11), and one fluoroquinolone-resistant strain (Cip15KB; Fig. 3B). By contrast, the multidrug-resistant *marR* mutant Cip8 was not susceptible to most extracts, exhibiting few CR or CS interactions. The cephalosporin-resistant cell wall strains (Cef1, Cef6, Cef7, Cef8) produced strong but variable patterns of both CR and CS across the entire natural product library. Uniformity of response between the four related mutant strains was low but the overall potency, either CR or CS, was high in relation to other strains. Some strains were similarly prone to strong CR and/or CS responses, such as the rifamycin-resistant strain Rif1. In total, 1469 extracts (1249 Actinobacteria and 220 *Burkholderia*) produced CS activity in at least one drug-resistant strain compared to wildtype.

CS profiles within the dataset fell into four main categories: first, CS in one drug-resistant strain; second, CS in multiple strains with mutations in the same gene; third, CS in multiple resistance genes within the same drug class; and fourth, CS in several drug classes. For example, one major cluster of extracts presented a CS profile that mirrored the CR profile shown in Fig. 3B, with increased activity against select MDR, rifamycin-, and fluoroquinolone-resistant strains (corresponding to category four; Fig. 3C). We also observed a group of extracts with potent CS activity extending across all aminoglycoside-resistant and plasmid-borne MDR strains (corresponding to categories three and four; Fig. 3D). Many extracts that elicited CR in a set of drug-resistant strains also induced CS in other strains, and vice versa. Together, these data suggests that the interaction between drug resistance and CS in natural products, and by extension in nature, is both complex and widespread.

### Hit triage and CS-active natural product discovery

A subset of 117 hit extracts were selected for follow-up from the primary screen based on the diversity and potency of their CS profiles. These extracts were prepared as half-step dilution series (16 two-fold dilutions) from their stock concentrations and rescreened against the full target panel (Supplementary Fig. 2). This secondary assay served to both verify the original CS activity from the primary screen and quantify the magnitude of the CS shift between WT and mutant MICs. Although the absolute concentrations of compounds in each prefraction are unknown, the relative difference in these MIC shifts between WT and drug-resistant strains provided a measure of the strength of the observed CS effect. In all cases, positive fold change values between the MICs indicated CR to the active compounds by the drug-resistant mutant, while negative fold change values indicated the presence of CS interactions. Of the 117 extracts selected for secondary screening, 72 extracts exhibited measurable CS activity with at least a two-fold difference in MIC for at least one drug-resistant strain. 39 out of 72 extracts displayed CS activity only, while 33 extracts presented a combination of CS and CR activities, depending on the drug-resistant strain. No extract exhibited solely CR interactions in the secondary assay.

The subset of 72 extracts was further triaged based on CS magnitude and profile diversity to give 28 prefractions for downstream chemical investigation. A high-throughput bioactivity-guided fractionation approach, termed the Peak Library system, was adopted to deconvolute these complex mixtures and to identify the active components. Details regarding the Peak Library system are provided in the methods section and have been described in previous work published by our group^28^. In brief, each extract was separated using reverse-phase high-performance liquid chromatography (RP-HPLC) into 80 discrete subfractions with 0-5 UV ‘peaks’ per fraction. These 28 peak libraries were then rescreened in a tertiary assay against relevant members of the drug-resistant target panel to identify active metabolites. Active subfractions were evaluated by high-resolution mass spectrometry (HRMS) analysis to obtain target masses as candidate leads for isolation.

### Discovery, isolation, and characterization of borrelidin natural products with CS activity against cephalosporin-resistant *E. coli*

**Fig. 4:**
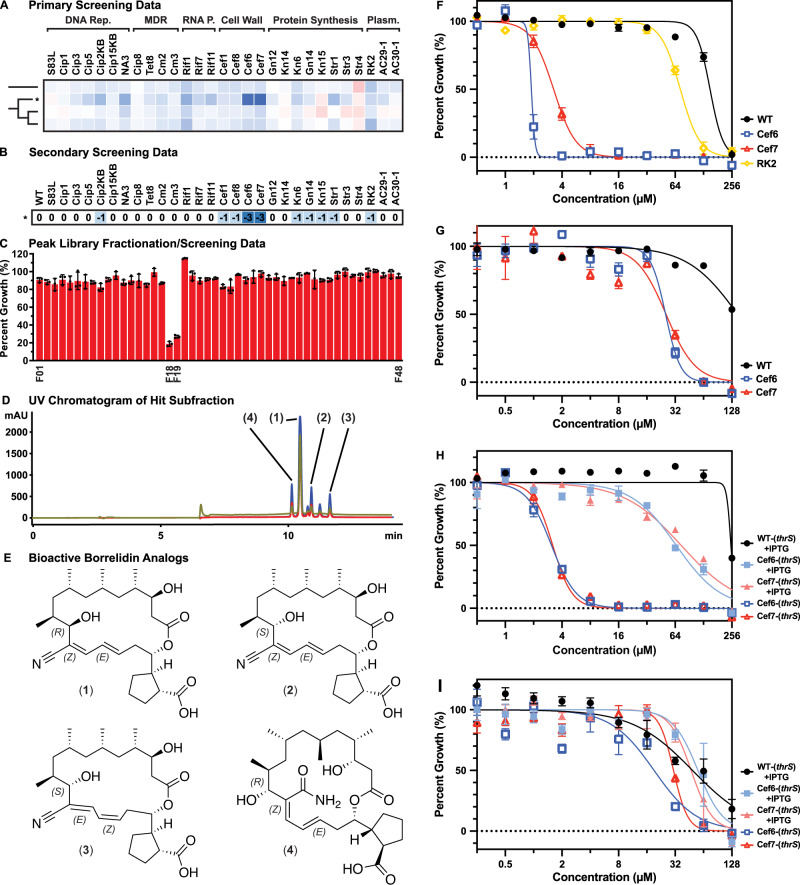
Discovery, isolation, and characterization of CS-active borrelidins. **A** Primary screening data and dendrogram of hit extract RLUS-1732D (*). **B** Secondary screening data of RLUS-1732D (*) as dilution series. Values represent Log_2_(MIC) difference from wildtype; blue = decreasing MIC values (CS). **C** Peak Library fractionation of hit extract RLUS-1732D and subsequent bioactivity testing pointed to two neighboring subfractions with strong inhibition against strain Cef7. **D** HPLC-UV chromatogram of bioactive subfractions F18/19 from RLUS-1732D. Red curve = 210 nm wavelength; blue curve = 254 nm; gold curve = 280 nm. **E** Structures of borrelidin A (**1**), F (**2**), H (**3**), and new compound borrelidin P (**4**). **F** Dose-response curves of wildtype *E. coli* MG1655 (WT), cephalosporin-resistant strains Cef6 and Cef7, and plasmid-borne multidrug resistant strain RK2 treated with **1**. **G** Dose-response curves of WT, Cef6, and Cef7 treated with **4**. **H** Dose-response curves of transformed *E. coli* MG1655, Cef6, and Cef7 with threonine—tRNA ligase gene *thrS*, either in the presence or absence of IPTG inducer, treated with **1**. **I** Dose-response curves of transformed *E. coli* MG1655, Cef6, and Cef7 with *thrS*, either in the presence or absence of IPTG inducer, treated with **4**. **C**, **F**–**I** Data shows the mean of the three independent experiments (*n* = 3); error bars denote standard error of the mean (SEM). Source data are provided as a Source Data file.

Throughout the triaging process, extract RLUS-1732D displayed particular promise in both the primary and secondary CSP screens. Primary screening data showed strong CS activities localized to the cephalosporin-resistant cell wall mutants, but also mild CS activity across multiple drug classes (Fig. 4A). This activity was recapitulated in the secondary assay, with an 8-fold MIC difference for cephalosporin-resistant strains Cef6 (*rfaH* W4*) and Cef7 (*rfaG* E289fs) and 2-fold MIC shifts for Cef1 (*envZ* T402M), Cef8 (*envZ* P248S), and several additional strains (Fig. 4B). Peak Library fractionation produced two discrete, bioactive subfractions (F18/F19) that clearly inhibited the growth of strain Cef7 (Fig. 4C). HPLC-UV-MS analysis of these subfractions revealed the presence of a family of related compounds with strong UV absorbance at 260 nm. In total, four bioactive analogues (**1**, **2**, **3**, and **4**) were isolated from a large-scale culture of the producing organism (RL09-056-NTSA; *Streptomyces* spp.; Fig. 4D). Using standard HRMS and nuclear magnetic resonance (NMR) techniques, compounds **1**, **2**, and **3** were identified as the known macrolide natural products borrelidin A, F, and H, respectively (Fig. 4E). Compound **4** did not match any entries in the Natural Products Atlas database of microbial natural product structures^29^, and so was subjected to full de novo structure elucidation using HRMS, 1D, and 2D NMR analyses (Supplementary Table 4). Detailed interpretation of these data identified compound **4** as a new natural product, named borrelidin P. The absolute configuration of this new metabolite was determined through a combination of dipolar coupling NMR experiments and chemical derivatization using the modified Mosher’s ester method^30^.

Post-purification, all four analogues were serially diluted as 16 two-fold dilutions (256 μM to 7.8 nM final assay concentration for **1**, **2**, and **3**; 128 μM to 4 nM for **4**) and rescreened against members of the target panel to obtain absolute MIC shifts for each strain. Compound **1** displayed the strongest CS activity, with 64-fold and 32-fold decreases in its MIC against cephalosporin-resistant strains Cef6 and Cef7, respectively (Fig. 4F). Furthermore, **1** showed a modest but consistent 2-fold decrease in MIC against strain RK2, which houses a well-known MDR plasmid from clinical isolates of *Klebsiella aerogenes*^19^. Interestingly, no CS activity was observed in the other two strains hosting MDR plasmids (pAC29 and pAC30), suggesting that the CS interaction is specific to the resistance genes within RK2. In congruence with both primary and secondary screening results, **1** remained CS-active against other cephalosporin-resistant strains Cef1 and Cef8, as well as fluoroquinolone-resistant strain Cip2KB (*gyrB* L509G) and aminoglycoside-resistant strain Kn15 (*ubiF* Q120*). However, the potency of these CS activities was reduced, with only 2- to 4-fold shifts in the IC_50_ (instead of full inhibition) depending on the strain (Supplementary Fig. 3A). Compounds **2** and **3** also displayed weak CS activity compared to **1**, with 4-fold and 2-fold decreases in MIC against Cef7, respectively (Supplementary Fig. 3B, C). Lastly, **4** produced an intermediate 8-fold decrease in MIC against both Cef6 and Cef7 (Fig. 4G).

Compound **1** was originally discovered due to its antibacterial activity against *Borrelia* sp., although its antimalarial activity and cytotoxicity have since been reported^31,32^. Mechanistic studies have established borrelidin A as a potent inhibitor of threonyl-aminoacyl tRNA synthetase (threonine—tRNA ligase) in both prokaryotic and eukaryotic targets^33,34^. This inhibition is contingent on an unusual nitrile functional group pendant on the macrocyclic core^35^. Interestingly, **4** displayed CS activity despite the substitution of a primary amide in place of the nitrile group. To test the hypothesis that the observed CS activity of **1** and **4** was related to bacterial threonine—tRNA ligase inhibition, an ASKA library vector containing *thrS* was transformed into WT, Cef6, and Cef7 *E. coli* strains. Induction of ThrRS overexpression, followed by treatment with **1**, resulted in a 32-fold increase in its MIC against strains Cef6 and Cef7 compared to uninduced control (Fig. 4H). This experiment was additionally replicated using **4**, where a 2-fold increase in MIC was observed for strains Cef6 and Cef7, compared to uninduced control (Fig. 4I). These results support the notion that ThrRS is the primary target of borrelidins in *E. coli* in the context of CS. However, it is unclear if ThrRS inhibition is the only mechanism of borrelidin activity as **4** lacks the key nitrile moiety needed for target binding but remains bioactive in strains Cef6 and Cef7. ThrRS overexpression also failed to fully revert the activities of compounds **1** and **4** back to WT levels; instead, the Hill slope curve for both compounds exhibited a shallower slope suggesting increased CS vulnerability at concentrations immediately below the MIC. Interestingly, ThrRS overexpression under identical conditions produced different effects on the MICs of **1** and **4**.The existence of multiple biological targets for the borrelidin family have been previously reported, including by our group, in multiple disease models (cyclin-dependent kinase in yeast and lactate dehydrogenase in *Plasmodium* sp.)^32,35^. Our results suggest that additional targets may exist in bacteria as well, and that this activity is magnified in select drug resistance phenotypes.

### Borrelidin A and ceftazidime are not synergistic in *E. coli*

We next sought to investigate whether the CS activity of the borrelidins was synergistic with the cephalosporin antimicrobial ceftazidime. Both Cef6 and Cef7 strains were selected on ceftazidime and have been determined to carry mutations that affect the lipopolysaccharide biosynthesis pathway. These mutations confer resistance against cephalosporins but also increased sensitivity to borrelidins in a CS context. We reasoned that the presence of ceftazidime in the WT strain may improve borrelidin activity (possibly through permeabilization) and vice versa (borrelidins destabilizing cell wall biosynthesis), thereby producing a synergistic interaction. To that end, ceftazidime and **1** (the most potent borrelidin analogue) were screened together using two-dimensional checkerboard assays against WT, Cef7, and the MDR RK2 strains. The β-lactam antibiotic ampicillin was used as a control for ceftazidime. Resulting absorbance data were normalized as percent growth values, with the combination of the lowest compound concentrations resulting in at least 50% inhibition of growth used to calculate the fractional inhibitory index (FIC). In WT *E. coli*, ceftazidime in conjunction with **1** produced strong additive interactions (FIC = 0.56) but not synergy (Supplementary Fig. 4A). The same was true for **1** with ampicillin (FIC = 0.63). Screening in the cephalosporin-resistant strain Cef7 reproduced the expected CS interaction: decreased activity for ceftazidime but increased activity for **1** (Supplementary Fig. 4B). However, the FIC remained unchanged (0.5), as was the case for **1** with ampicillin (FIC = 0.5). Lastly, we tested the MDR strain RK2 as a CS control that do not possess cell wall resistance mutations; an additive interaction was again observed (FIC = 1; Supplementary Fig. 4C). These data suggest that the borrelidins’ CS activity is not an extension of synergy with ceftazidime but is instead the result of the presence specific mutations causing cephalosporin resistance.

### Cef6/7 mutations do not increase intracellular concentrations of borrelidin A

To test the hypothesis that borrelidin CS activity is due to greater permeabilization in the cell wall biosynthesis mutants, we next performed a mass spectrometry-based compound accumulation study. Cef6 and Cef7 strains, as well as WT, were separately incubated with either vehicle, borrelidin A (**1**), or mupirocin (a control compound that also inhibits protein synthesis via isoleucyl-tRNA synthetase) at their respective IC_50_ for 10 min. Cells of each strain/treatment condition were harvested, with the vehicle control serving to demonstrate specificity of selection product ion transitions. Following the removal of cell culture supernatants, cells were lysed to permit absolute quantification of internalized compounds by targeted liquid chromatography-mass spectrometry (LC-MS) using multiple reaction monitoring (MRM). LC-MS-based quantification revealed no statistically significant increase in the intracellular concentration of **1** between the three strains (Supplementary Fig. 5). Nor was there a difference in the extracellular or intracellular concentrations of the mupirocin control. The increased antimicrobial activity of **1** observed in strains Cef6 and Cef7 is therefore not the result of increased internalization, further supporting the existence of additional biological targets for this family of compounds.

### Ceftazidime-borrelidin A co-dosing regimen limits emergence of ceftazidime resistance in *E. coli*

**Fig. 5:**
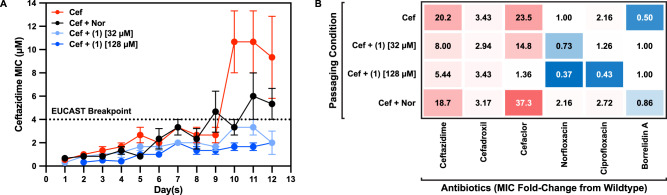
Sublethal doses of borrelidin A slow the emergence of cephalosporin resistance in wildtype *E. coli*. **A** Ceftazidime MIC values as determined through sequential passaging of wildtype *E. coli* MG1655 over twelve days under increasing ceftazidime drug pressure, either alone (red; Cef), with an increasing concentration of norfloxacin (black; Nor), with a constant concentration of **1** at 32 µM (light blue), or with a constant concentration of **1** at 128 µM (blue). Populations that reached 50% growth under the highest concentration of ceftazidime for each treatment condition were used to inoculate the following day’s cultures. Dotted line represents the EUCAST breakpoint for ceftazidime resistance in *E. coli*. Data shows the mean of three independent passaging experiments (*n* = 3); error bars denote the standard error of the mean (SEM). **B** On the 12th day, strains from each passaging condition were tested with three cephalosporins (ceftazidime, cefadroxil, cefaclor), two fluoroquinolones (norfloxacin and ciprofloxacin), and **1** in triplicate. Values indicate average fold-change MICs compared to wildtype across three independent experiments (*n* = 3). Source data are provided as a Source Data file.

One potential application of CS agents is to co-dose them with the corresponding antimicrobial for which resistant strains show CS behavior. In this application, the CS drug would act as an adjuvant to eliminate evolving drug-resistant populations. To investigate this application, we tested the ability of **1** to limit the emergence of cephalosporin resistance in *E. coli* under increasing drug pressure. WT K-12 MG1655 was subjected to four separate drug treatments: ceftazidime alone, ceftazidime with **1** at 32 μM, ceftazidime with **1** at 128 μM, and ceftazidime with norfloxacin as a negative control (no CS relationship with ceftazidime-resistant strains). Cultures were passaged daily under increasing concentrations of ceftazidime, based on MIC values. Figure 5A illustrates ceftazidime resistance acquisition over time under each of these four conditions. Treatment with ceftazidime alone resulted in a steady increase in MIC over the first 9 days, followed by a significant increase to 8 μM MIC on the 10th day, surpassing the EUCAST^36^ breakpoint for ceftazidime resistance in *E. coli* (>4 μM). Co-dosing with **1** at 32 μM (1/8th its MIC in WT *E. coli*) marginally suppressed the average MIC compared to ceftazidime alone and prevented resistance from reaching the EUCAST breakpoint for 12 days. Increasing **1** treatment concentration to 128 μM (its IC_50_ in WT *E. coli*) reduced resistance by up to 4-fold compared to ceftazidime alone and stabilized resistance between 1 to 2 μM for up to 12 days. In contrast, co-dosing ceftazidime with norfloxacin failed to significantly suppress the emergence of ceftazidime resistance, reaching the breakpoint as early as the 9th day (Supplementary Fig. 6).

At the conclusion of the serial passaging experiment, cultures from each condition were subjected to treatment with three cephalosporin drugs (ceftazidime, cefadroxil, cefaclor), two fluoroquinolones (norfloxacin, ciprofloxacin), and **1** (Fig. 5B). After 12 days, ceftazidime drug pressure resulted in a 20-fold increase in MIC for ceftazidime, but also a 24-fold increase in CR to cefaclor, highlighting the prevalence of intra-class cross-resistance. Ceftazidime treatment also produced CS behavior with **1**, demonstrating the reproducibility of this interaction. Co-dosing with **1** at 32 μM reduced ceftazidime and cefaclor resistances to 8-fold MIC and 15-fold MIC, respectively. Increasing the concentration of **1** to 128 μM reduced cephalosporin resistance even further, to 5-fold MIC for ceftazidime and negligible increase for cefaclor. Co-dosing with norfloxacin, however, failed to change ceftazidime resistance significantly (19-fold MIC) but greatly exacerbated cefaclor CR (37-fold MIC), illustrating one of the risks of ineffective co-dosing selection. Norfloxacin treatment furthermore produced mild resistance towards the two fluoroquinolone compounds. Yet, resistance against **1** was not observed for any treatment with **1**; a mild CS interaction was instead detected with the fluoroquinolones. These results conclude that borrelidin NPs are capable of suppressing the emergence cephalosporin resistance in *E. coli*.

## Discussion

Recent studies have demonstrated the value of exploiting CS using antimicrobial chemistry to curtail the emergence of drug resistance^10,11,13,37^. In this work, we present a high-throughput screening platform, termed CSP, for the systematic identification of CS-active compounds in large chemical libraries. CSP leverages a diverse single mutant target panel to facilitate annotation of the gene/pathway responsible for resistance, and by extension highlight one-compound-to-one-gene CS interactions. Initial screening of 80 commercial antimicrobials covering 30 drug classes using CSP revealed both known and novel CS relationships (Fig. 2). Imamovic and Sommer have previously identified CS interactions between aminoglycoside-resistant strains and multiple antimicrobials, including cefuroxime, tetracyclines, and chloramphenicol^10^. We have observed similar CS interactions in our aminoglycoside-resistant strains against these same compounds and/or same-class analogues, suggesting an underlying CS relationship despite variations in the specific mutations that confer aminoglycoside resistance. Interestingly, the aminoglycoside resistance-chloramphenicol CS interaction shown in this study extended to the fluorinated methyl-sulfonyl chloramphenicol analogue florfenicol, but not the defluorinated methyl-sulfonyl analogue thiamphenicol, further suggesting that this class of compounds possesses a unique structure-activity relationship with its CS target. In addition, all three amphenicols exhibited reproducible CS activity in both plasmid-borne MDR strains carrying clinical resistance cassettes from the IncF incompatibility group^18^. Whether these CS relationships derive from a common CS target or are caused by different CS relationships for each resistant strain remains an open question, but the consistent CS profiles between members of the same antibiotic class demonstrates the varied and pervasive nature of CS relationships in drug-resistant populations.

Subsequent CSP screening of our 6195-member natural product extract library also revealed widespread CS interactions in nature (Fig. 3). To our knowledge, this screen represents the largest and most comprehensive examination of antimicrobial CS in natural products to date. In contrast to previous CS studies which have focused on known antimicrobials, or in one instance antimicrobial peptides^11^, NPs offer unparalleled chemical diversity and biological relevance from a therapeutic perspective^27^. The broader conclusion from this study is that CS interactions are prevalent in NP libraries, and are compound class specific, suggesting that a large number of CS agents remain to be discovered. The discovery of NP-based CS agents differs from conventional NP adjuvant or synergy screens where a primary antibiotic is co-dosed along with the test extract to discover NPs that restore the activity of the primary compound. Instead, CSP exclusively identifies extracts with preferential activity against drug-resistant mutants over WT, where the CS agent is itself active against the resistant strain.

Using a set of triage protocols and secondary screening procedures, hit extracts from the library were examined in detail. These efforts led to the isolation of the borrelidin family of NPs, with borrelidin A (**1**) producing MICs 128- and 64-fold lower in the Cef6 (*rfaH* W4*) and Cef7 (*rfaG* E289fs) strains compared to WT, respectively. Several studies from the existing literature help to contextualize this finding. First, knockouts of genes responsible for lipopolysaccharide (LPS) biosynthesis (*rfa*) in *E. coli* have been shown to increase resistance against β-lactams, including cephalosporin compounds^38–40^. These mutants produce a variably truncated LPS core that discourages trimerization of outer membrane porins, which themselves facilitate the entry of small polar drugs. Recently, Wang et al. reported that truncated LPS mutants exhibit decreased transcription and expression of porins OmpF and OmpC, but increased transcription of the β-lactam resistance gene *blr*^39^. Second, both laboratory and clinical isolates of β-lactam-resistant *E. coli* have been found to contain mutations in *thrS*^41^, the known target of the borrelidins. Studies have shown that inactivation of ThrRS results in a stringent response that reduces bacterial growth rate, which contributes to the observed resistance phenotype^41,42^. In this study, we show that **1** is a concentration-dependent inhibitor of ThrRS that preferentially kills cephalosporin-resistant *E. coli* (with deleterious mutations in *rfa* genes) as well as a MDR strain carrying the RK2 plasmid from clinical isolates of *K. aerogenes*^19^ (Fig. 4). We furthermore demonstrate that this CS activity cannot be replicated by combination therapy between **1** and ceftazidime in the WT, and is therefore solely the result of the resistance mutations (Supplementary Fig. 4). Several studies have found that *rfa* mutants exhibit increased sensitivities to large, nonpolar antibiotics due to truncated LPS and thus a weaker protective barrier^38^; a finding corroborated in our own antimicrobial screen (Supplementary Fig. 1). While this offers a possible explanation for the borrelidins’ CS activity, quantitative mass spectrometry has shown that the intracellular concentrations of **1** do not increase between WT and the cephalosporin-resistant *rfa* mutants (Supplementary Fig. 5). This finding refutes increased influx, decreased efflux, and inactivation/digestion of **1** as the cause for CS. The combination of existing evidence would suggest that the loss of biological fitness owing to both *rfa*-related LPS truncation and ThrRS inhibition is responsible for the observed CS interaction.

The cytotoxicity exhibited by the known borrelidins precludes their use in a clinical context,^35,43^ although the toxicity profile for the new desnitrile analogue **4** has yet to be determined. Compound **4** remains active in a CS context despite lacking the nitrile moiety widely reported to be essential for ThrRS inhibition. ThrRS overexpression only weakly attenuates the activity of **4** compared to **1**, and to a much lesser extent, suggesting that alternative targets may play a role in the observed CS activity. This finding is consistent with previous studies that have found secondary targets of **1** in other systems^32,35^.

Together, the borrelidins serve as valuable proof-of-concept that NPs inactive against wildtype pathogens may possess potent CS activities against their drug-resistant variants. CSP of our NP library has revealed plentiful examples of such relationships, with unique distributions of CS activity in a host of diverse drug-resistant strains. Because the CS-active compounds prioritized from this screen are selectively active against drug-resistant populations, they are typically only weakly active against WT strains, making them unsuitable as frontline monotherapies. Instead, the value of CS agents stems from their ability to selectively target drug-resistant populations via mechanisms that are distinct from partner antimicrobials^11^. Co-dosing **1** at either 32 or 128 μM (sub-MIC concentrations in WT) with ceftazidime suppressed ceftazidime resistance below the EUCAST^36^ breakpoint for *E. coli* (4 μM) over a period of 12 days (Fig. 5A). By contrast, in the CS-null condition (ceftazidime co-dosed with norfloxacin), resistance to ceftazidime evolved at the nearly the same rate as ceftazidime alone, despite the presence of two antibiotics with different mechanisms of action. Furthermore, the resulting drug-resistant strains showed extensive CR to other cephalosporins and fluoroquinolones (and thus MDR; Fig. 5B). Co-dosing with **1** presented no self-resistance and instead led to the emergence of CS to fluoroquinolones, supporting the fundamental finding that CS manifests in fitness trade-offs concomitant with the evolution of drug resistance. Together these results pave the way for the discovery of a new suite of natural product-based CS agents with direct application in the prevention and treatment of AMR infections.

## Methods

### Generation of drug-resistant *E. coli* strains

Isolation of ciprofloxacin-resistant and nalidixic acid-resistant strains was performed as described above. Additional single-step AMR mutants of *Escherichia coli* K-12 MG1655 were selected as reported previously for ciprofloxacin^44^. Briefly, 200 μL aliquots of lysogeny broth (LB; 5 g/L yeast extract, 10 g/L tryptone, 10 g/L NaCl) were inoculated with a single colony each and grown overnight at 37 °C with 150 rpm shaking in 96-well plates. Each liquid culture was then plated in its entirety on an LB agar plate containing one of the following antibiotics: tetracycline (8.44 μM), chloramphenicol (49.5 μM), rifampicin (60.8 μM), ceftazidime (1.14 μM), gentamicin (7.85 μM), kanamycin (15.5 μM), streptomycin (13.8 μM), or ampicillin (17.9 μM). To ensure collection of independent mutants, 20 replicate plates were generated for each antibiotic, and a single colony was picked from each plate showing growth.

Whole-genome sequencing was carried out on each AMR mutant. Bacterial cultures were grown overnight in 5 mL LB; DNA was extracted using the BioBasic One-4-All Genomic DNA Mini-prep Kit (Bio Basic, Markham, Ontario, Canada) according to the manufacturer’s instructions. Sequencing was carried out using the MiSeq platform with Nextera XT DNA library preparation and paired-end 250 basepair reads. Raw FASTQ sequence files were trimmed using Trimmomatic v.0.35^45^, with parameters LEADING:20 TRAILING:20 SLIDINGWINDOW:4:20 MINLEN:36. FastQC was used to quality check trimmed FASTQ files (https://www.bioinformatics.babraham.ac.uk/projects/fastqc/). Reference-guided assembly was carried out using Bowtie2, with the *E. coli* K-12 (MG1655) genome as a reference (GenBank NC_000913.2). Mutation effects were predicted using SnpEff^46^. Strains carrying the multidrug resistant plasmids RK2, pAC29 and pAC30 were generated by CaCl_2_ transformation, as described below for ThrRS overexpression strains. The IncF plasmids pAC29 and pAC30 were taken from clinical isolates of *E. coli* PB29 and PB30^18^, while RK2 is a well-described vector from MDR *K. aerogenes* isolates^47^.

### Commercial antimicrobial collateral sensitivity profiling

Drug-resistant *E*. *coli* strains were inoculated from glycerol stocks into 3 mL overnight cultures in LB media (Fisher), growing at 37 °C, 200 rpm. Saturated overnight cultures were diluted in LB media according to turbidity to achieve ~5 × 10^5^ CFU/mL of final inoculum density and dispensed (Matrix WellMate) into sterile polystyrene 384-well microplates (Thermo Scientific^TM^ 265202) with a final screening volume of 30 μL. Antimicrobials (for a comprehensive list and manufacturer see Supplementary Table 2) were prepared in either dimethyl sulfoxide (DMSO) or water to produce stock solutions of 19.2 mM. Antimicrobial stocks were transferred to 384-well compound plates (ABGene 3487) and reformatted as 1:1 dilution series, before being pinned into each respective assay plate (200 nL) using a high-throughput pinning robot (Tecan Freedom EVO 100; V&P Scientific pin tool) to achieve final screening concentrations in the range of 128 μM to 3.91 nM. In each 384-well assay plate, column 1 was reserved for blank control (DMSO vehicle; LB media), column 2 was reserved for growth control (DMSO vehicle; LB media; target bacteria), and columns 23/24 reserved for strain-specific antimicrobial controls (DMSO vehicle, LB media; target bacteria; two of ciprofloxacin, tetracycline, chloramphenicol, rifampicin, ceftazidime, gentamicin, or ampicillin depending on target bacteria selection conditions). After compound pinning, assay plates were read using a plate reader (Molecular Devices SpectraMax i3x running SoftMaxPro software; BioTek Synergy Neo2 running GEN5 software) to obtain OD_600_ absorbance values at t_0_, sealed with lids and placed in a humidity-controlled incubator (Thermo Cytomat) at 37 °C, 5% CO_2_ for 20 h. Post-incubation, OD_600_ readings were taken at t_20_. Post-data acquisition, absorbance values were normalized by subtracting t_0_ values from the corresponding t_20_ values and then dividing by the average of the difference of the blank and growth controls. The MIC for each compound against each strain was determined as the lowest concentration for which there was 90% inhibition compared to controls. The full screening protocol was repeated three times to obtain three independent biological replicates. The Z’-factor for the antimicrobial CSP screen was ascertained by first calculating the Z’-factor for all four compound plates against each test strain, then averaging across three replicates, and finally averaging again across all thirty strains (Supplementary Table 3). For a robust Z’-factor calculation, each antimicrobial that presented a discernable MIC value was considered a control along with compounds assigned to columns 23/24 of each plate; positive control wells were defined as those above the MIC while negative control wells were defined as below the MIC for any given antimicrobial/control compound.

### Natural product extract library preparation and screening

Cell culturing, extraction, library preparation, and crude extract fractionation were performed in the following sequence. Actinobacterial strains were isolated from marine sediment collected from the North American west coast including sites in California, Oregon, and Washington State^28,35^. *Burkholderia* strains were isolated using selection medium from various soil samples collected in British Columbia, Canada^48^. Bacterial isolates were grown under standard fermentation conditions^28,35,48^, extracted using 1:1 methanol/dichloromethane, and fractionated using reverse-phase C18 cartridges with an eluotropic methanol/water step gradient (20%, 40%, 60%, 80%, 100% *v/v*) followed by a 100% ethyl acetate wash, affording six prefractions per extract. Prefractions were concentrated to dryness *in vacuo*, resuspended in 1 mL of DMSO, and stored in 96-deepwell plates at −70 °C. Replicates of DMSO stock solutions were reformatted into 384-well plates for screening, which proceeded via the protocol outlined above. Only one biological replicate was acquired due to material limitations of the library. For the natural products screen, the Z-factor was calculated instead of the Z’-factor to better control for the discrepancy in signal between complex extracts and pure antimicrobials. This was achieved by replacing the original negative controls (compound well values below the MIC) in the Z’-factor calculation with that of the screening data in its entirety, thereby incorporating sample variability^49^. The interquartile range method was used to reject outliers and prevent hits from skewing the data. See Supplementary Table 3 for a list of Z-factor values for each strain and the equation used.

### Data analysis and secondary screening

Primary screening data was subjected to plate-based normalization as described above, then normalized again across all plates for each given strain to standardize the maximum growth. Lastly, WT normalized percent growth values for each extract were subtracted from their respective strains’ growth values such that positive values inferred increased resistance while negative values indicated collateral sensitivity. Processed screening data were uploaded to the Morpheus data analysis platform (https://software.broadinstitute.org/morpheus/) and hierarchically clustered using Spearman-Rank correlation and average linkage parameters^50^. 117 hit extracts were selected from this analysis for secondary screening. DMSO library stocks of these prefractions were reformatted into 16 two-fold dilutions in 384-well microplates starting from their stock concentrations. Secondary screening and subsequent data processing were performed as described for the primary NP screen. Twenty-eight prefractions were selected for further chemical investigation from the secondary screen based on the magnitude and reproducibility of their CS profiles.

### Microbial fermentation and natural product extraction

Frozen stocks (1:1 glycerol:SYP media; −70 °C) of each of the 28 producing organisms were plated on solid MB media (per 1 L dH_2_O: DIFCO^TM^ Marine Broth, 37.4 g; agar, 15.0 g) at RT for up to 5 days. Discrete colonies were used to inoculate 7 mL of SYP liquid media (per 1 L dH_2_O: Instant Ocean, 31.2 g; Soluble Starch, 10 g; Yeast Extract, 4 g; Peptone, 2 g) with glass beads, shaking at room temperature (RT), 200 rpm for 4 days. 3 mL of this liquid culture was then used to inoculate 60 mL of SYP liquid media with a coiled stainless-steel spring, shaking at RT, 200 rpm for an additional 5 days. 45 mL of the previous culture was used to inoculate 1 L of SYP liquid media inside a sterile 2.8 L wide-neck Fernbach flask containing a larger coiled stainless-steel spring and XAD-17 resin (20 g), shaking at RT, 200 rpm for another 7 days. After fermentation, the large-scale culture was filtered with Whatman paper, and the residue extracted with DCM/MeOH (1:1) three times while stirring. The resulting suspension was filtered again to remove cellular debris and torn filter paper. Celite resin (20 g) was added to the filtrate and evaporated to dryness *in vacuo*. Extract-adhered dry resin was packed into a reusable prep column and prefractionated using the CombiFlash via an eluotropic series of MeOH/H_2_O as described for NP library preparation. The appropriate fractions were collected separately and concentrated to dryness under vacuum for further analysis. Resulting prefractions corresponding to hits from the secondary screen were rescreened against selected strains to ensure reproducibility of activity. Bioactive prefractions from the fermentation step were then prioritized for subsequent bioassay-guided fractionation via the Peak Library system.

### Peak library bioactivity-guided fractionation system

For each bioactive extract, a standardized volume of the DMSO stock solution was transferred from storage at −70 °C, evaporated to dryness *in vacuo*, resuspended in MeOH/H_2_O (1:1), and centrifuged to remove particulate. The resulting supernatant was fractionated via HPLC-MS (Phenomonex Kinetix 2.6 µm XB-C18 150 × 4.6 mm) into 80 discrete subfractions in a 96-deep-well microplate. HPLC gradients used for fractionation varies based on the prefractionation process of each extract. Prefraction A (20% MeOH/H_2_O wash): gradient of MeOH/H_2_O + 0.02% formic acid (5% MeOH for 5 min, 5% to 40% MeOH over 30 min, 100% MeOH for 8 min) at a flow rate of 1 mL min^−1^, collecting 1 subfraction every 0.5 min starting at 5 min. Prefraction B (40% MeOH/H_2_O wash): gradient of MeOH/H_2_O + 0.02% formic acid (5% MeOH for 5 min, 10% to 60% MeOH over 30 min, 100% MeOH for 8 min) at a flow rate of 1 mL min^−1^, collecting 1 subfraction every 0.5 min starting at 5 min. Prefraction C (60% MeOH/H_2_O wash): gradient of MeOH/H_2_O + 0.02% formic acid (5% MeOH for 5 min, 30% to 80% MeOH over 30 min, 100% MeOH for 8 min) at a flow rate of 1 mL min^−1^, collecting 1 subfraction every 0.5 min starting at 5 min. Prefraction D (80% MeOH/H_2_O wash): gradient of MeOH/H_2_O + 0.02% formic acid (5% MeOH for 5 min, 50% to 100% MeOH over 30 min, 100% MeOH for 8 min) at a flow rate of 1 mL min^−1^, collecting 1 subfraction every 0.5 min starting at 5 min. Prefraction E (100% MeOH wash): gradient of MeOH/H_2_O + 0.02% formic acid (5% MeOH for 5 min, 70% to 100% MeOH over 30 min, 100% MeOH for 8 min) at a flow rate of 1 mL min^−1^, collecting 1 subfraction every 0.5 min starting at 5 min. 96-deep-well microplates containing extract subfractions were concentrated to dryness using a Thermo SpeedVac concentrator (heating at 40 °C for 8 h, 1200 rpm). Dry extracts were resuspended in 10 µL of DMSO and reformatted into 384-well compound plates. Screening of peak libraries proceeded as described for the primary and secondary NP screens.

### Natural product isolation and discovery

Hit extract prefraction RLUS-1732D (produced by *Streptomyces* spp. strain RL09-056-NTS-A) was profiled by UPLC-HRMS using a Waters SYNAPT G2Si ESI-qTOF-MS system running MassLynx software and LC-MS using an Agilent 6130 MS instrument running ChemStation. Four related analogs with UV maxima at ~260 nm and retention times between 10.0 to 12.0 min were prioritized for purification based on its Peak Library screening data. The major component, possessing a *m/z* of 512.2981 [M + Na]^+^ was purified by HPLC to yield 9.82 mg of a white amorphous powder. Extensive 1D and 2D NMR analyses (Bruker Avance III 600 MHz with 5 mm QCI cryoprobe running Topspin; data processing performed with MNova) identified this compound as the known macrolide borrelidin A (**1**; Supplementary Fig. 7–9). Eleven other borrelidin analogs produced by RL09-056-NTSA were isolated across the A, B, C, and D prefractions at microgram quantities and assayed against select mutant strains. While **1** presented the strongest MIC shift, three additional analogs also presented recognizable CS activities. Two analogs shared identical masses to borrelidin A but eluted later at 11.1 and 12.3 min, respectively. These corresponded to the conformational analogs borrelidins F (**2**; Supplementary Fig. 10–14) and H (**3**; Supplementary Fig. 15–19) which differ at the configurations of the alcohol at C11 (**2**) or the olefin at C14 (**3**). The fourth bioactive analog eluted earlier than **1** at 10.2 min and possessed a formula that did not correspond to any known borrelidin derivatives^43,51–54^. Consequently, this new molecule was named borrelidin P (**4**). Interpretation of HRMS and NMR data (Supplementary Fig. 20–30) suggested the presence of a primary amide for **4** in place of the nitrile functional group in **1** (Supplementary Fig. 31). An identical 2D structure had recently been reported as a semisynthetic derivative of **1** by Hu et al.^55^. However, comparison of the NMR data between **4** and the semisynthetic derivative suggested that the new compound possessed a different configuration to **1** at one or more positions. To verify this result, we replicated the semisynthetic transformation of **1** reported by Hu et al., which yielded 12-desnitrile-12-carbamoyl-borrelidin A (**5**; Supplementary Fig. 32–33). The NMR spectra of **5** mirrored that of the published semisynthetic derivative and was different from the natural product. Detailed interpretation of the 2D NOESY spectrum for **4** and comparisons against both **1** and **5** revealed an alternative 12-(*Z*)*−*14-(*E*) geometry in the diene region of the natural product (as opposed to a 12-(*E*)-14-(*E*) arrangement for **5**), completing the planar structure determination for this new metabolite. To assign the absolute configuration of **4**, the natural product was derivatized via a series of reactions to facilitate the eventual conjugation of the modified Mosher’s ester on secondary alcohols. Compound **4** was first protected by esterification of the carboxylic acid at C23 to produce the methyl ester-capped intermediate (**6**; Supplementary Fig. 34–40). Next, **6** was linearized using a base-catalyzed reaction to open the macrolactone ring, resulting in the linearized borrelidin P methyl diester (**7**; Supplementary Fig. 41–45). Lastly, **7** was reacted with (*R*)-Mosher’s acid chloride to form the linearized borrelidin P methyl diester (*S*)-MTPA conjugate (**8**, Supplementary Fig. 46–52). Due to material limitations, only the (*S*)-MTPA conjugate was made with the Mosher’s analysis performed instead using the variable temperature method reported by Latypov et al.^56^ Chemical shift differences for protons adjacent to the secondary alcohols at C3 and C11 were determined to be 3-(*S*)−11-(*R*) (Supplementary Table 5. For a detailed summary of the structure elucidation details for **4**, please refer to Supplementary Note 1.

Borrelidin A (**1**): white amorphous powder; ^1^H-NMR (600 MHz, CD_3_OD): δ 6.92 (d, *J* = 11.3, 1H), 6.61 (dd, *J* = 14.2, 11.8, 1H), 6.33 (ddd, *J* = 14.8, 10.4, 4.6, 1H), 4.98 (d, *J* = 10.2, 1H), 4.19 (d, *J* = 9.8, 1H), 3.94 (d, *J* = 8.8, 1H), 2.67 (dt, *J* = 17.2, 9.0, 1H), 2.59 (ddd, *J* = 14.6, 10.5, 4.1, 1H), 2.53 (d, *J* = 15.0, 1H), 2.40 (d, *J* = 14.8, 1H), 2.24 (dd, *J* = 16.0, 10.2, 1H), 2.02 (overlap), 2.01 (overlap), 1.88 (overlap), 1.86 (overlap), 1.82 (overlap), 1.81 (overlap), 1.78 (overlap), 1.63 (overlap), 1.42 (m, 1H), 1.24 (t, *J* = 12.4, 1H), 1.19 (t, *J* = 11.8, 1H), 1.09 (ddd, *J* = 14, 11.4, 2.8, 1H), 1.04 (d, *J* = 6.4, 3H), 0.99 (overlap), 0.97 (overlap), 0.87 (d, *J* = 6.1, 3H), 0.87 (d, *J* = 6.1, 3H), 0.85 (d, *J* = 6.1, 3H), 0.70 (t, *J* = 12.2, 1H); ^13^C-NMR (150 MHz, CD_3_OD): δ 173.3, 145.5, 140.3, 128.9, 119.9, 117.4, 77.5, 73.1, 72.9, 49.4, 47.5, 44.6, 39.1, 37.9, 37.1, 36.7, 35.9, 32.6, 30.5, 28.5, 27.6, 26.2, 20.9, 19.1, 18.7, 15.4; UV/Vis: λ_max_ 260 nm; HRMS (*m/z*): [M + Na]^+^ calcd. for C_28_H_43_NNaO_6_, 512.2983; found, 512.2981.

Borrelidin F (**2**): white amorphous powder; ^1^H-NMR (600 MHz, DMSO-d_6_): δ 6.79 (d, *J* = 11.1, 1H), 6.36 (t, *J* = 12.9, 1H), 6.01 (ddd, *J* = 14.5, 10.2, 4.4, 1H), 4.99 (tt, *J* = 7.5, 3.0, 1H), 3.71 (overlap), 3.70 (d, *J* = 8.2, 1H), 2.59 (m, 1H), 2.38 (overlap), 2.38 (overlap), 2.29 (dd, *J* = 15.6, 3.9, 1H), 2.09 (dd, *J* = 15.5, 8.5, 1H), 1.84 (m, 1H), 1.77 (overlap), 1.75 (overlap), 1.68 (overlap), 1.66 (overlap), 1.63 (overlap), 1.59 (m, 2H), 1.52 (m, 1H), 1.35 (m, 1H), 1.22 (overlap), 1.04 (ddd, *J* = 14.1, 11.3, 3.6, 1H), 0.96 (overlap), 0.96 (overlap), 0.91 (d, *J* = 6.5, 3H), 0.81 (m, 1H), 0.77 (d, *J* = 6.4, 3H), 0.74 (d, *J* = 6.1, 3H), 0.74 (d, *J* = 6.1, 3H), 0.68 (ddd, *J* = 13.8, 10.7, 3.6, 1H); ^13^C-NMR (150 MHz, DMSO-d_6_): δ 144.2, 140.5, 128, 77.1, 74.6, 69.3, 47.8, 47.4, 42.3, 37.5, 36.8, 36.6, 34.8, 34.6, 30.6, 28.7, 26.4, 25.7, 24.8, 20.1, 17.7, 17.7, 15.5; UV/Vis: λ_max_ 260 nm; HRMS (*m/z*): [M + H]^+^ calcd. for C_28_H_44_NO_6_, 490.3163; found, 490.3172.

Borrelidin H (**3**): white amorphous powder; ^1^H-NMR (600 MHz, DMSO-d_6_): δ 7.12 (d, *J* = 11.8, 1H), 6.46 (dd, *J* = 11.3, 1H), 6.02 (td, *J* = 10.4, 7.0, 1H), 4.94 (dt, *J* = 8.6, 4.0, 1H), 3.81 (d, *J* = 7.7, 1H), 3.75 (dt, *J* = 9.5, 2.9, 1H), 2.67 (m, 1H), 2.39 (overlap), 2.33 (m, 1H), 2.23 (dd, *J* = 15.9, 2.3, 1H), 2.01 (dd, *J* = 15.9, 10.3, 1H), 1.86 (overlap), 1.86 (overlap), 1.80 (m, 1H), 1.71 (overlap), 1.69 (overlap), 1.65 (overlap), 1.64 (overlap), 1.52 (m, 1H), 1.36 (m, 1H), 1.11 (overlap), 1.08 (overlap), 1.06 (overlap), 0.93 (overlap), 0.91 (d, *J* = 6.6, 3H), 0.88 (overlap), 0.78 (overlap), 0.76 (overlap), 0.75 (overlap), 0.68 (t, *J* = 11.3, 1H); ^13^C-NMR (150 MHz, DMSO-d_6_): δ 138.3, 135.2, 126.6, 76.4, 74.7, 70.3, 47.9, 47.5, 43, 36.7, 35.2, 34.9, 30.9, 30.6, 28.6, 26.7, 26.1, 24.7, 20.1, 19.1, 18.7, 15.0; UV/Vis: λ_max_ 258 nm; HRMS (*m/z*): [M + H]^+^ calcd. for C_28_H_44_NO_6_, 490.3163; found, 490.3170.

Borrelidin P (**4**): white amorphous powder; ^1^H-NMR (600 MHz, CD_3_OD): δ 6.72 (dd, *J* = 14.8, 11.5, 1H), 6.19 (d, *J* = 11.0, 1H), 5.82 (ddd, *J* = 14.7, 10.0, 4.2, 1H), 5.06 (t, *J* = 8.6, 1H), 3.84 (td, *J* = 6.0, 3.1, 1H), 3.74 (d, *J* = 8.6, 1H), 2.57 (overlap), 2.53 (overlap), 2.40 (overlap), 2.36 (overlap), 1.96 (overlap), 1.87 (overlap), 1.85 (overlap), 1.76 (overlap), 1.74 (overlap), 1.74 (overlap), 1.71 (overlap), 1.64 (overlap), 1.63 (overlap), 1.39 (overlap), 1.37 (overlap), 1.14 (ddd, *J* = 13.0, 9.8, 3.9, 1H), 1.07 (m, 1H), 1.03 (overlap), 0.98, (d, *J* = 6.3, 3H), 0.95 (overlap), 0.86 (d, *J* = 6.6, 3H), 0.85 (overlap), 0.85 (d, *J* = 6.7, 3H), 0.82 (d, *J* = 6.8, 3H); ^13^C-NMR (150 MHz, CD_3_OD): δ 183.6, 174.7, 172.6, 137.9, 136.4, 135.5, 130.0, 83.0, 77.7, 71.6, 49.5, 49.0, 44.0, 40.6, 39.1, 38.8, 37.0, 35.9, 33.1, 30.6, 28.1, 27.8, 26.3, 20.8, 19.3, 16.7, 16.5; UV/Vis: λ_max_ 249 nm; HRMS (*m/z*): [M + H]^+^ calcd. for C_28_H_46_NO_7_, 508.3269; found, 508.3280.

### Synthesis of 12-desnitrile-12-carbamoyl-borrelidin A (5)

The following procedure is adapted from Hu et al.^55^: (Supplementary Fig. 53) Borrelidin A (**1**; 10 mg; 0.021 mmol; 1 eq.), acetaldehyde oxime (Syn/Anti; 9.24 mg; 0.157 mmol; 7.5 eq.), copper oxide (CuO; 1.76 mg; 0.022 mmol; 1 eq.), and MeOH/H_2_O (1:1 *v/v*; 4 mL) were added to a 10 mL conical-bottom flask. The reaction mixture was heated to reflux at 150 °C for 60 h. A small aliquot was removed every 12 h to monitor reaction progress via HPLC-MS. After completion, the reaction mixture was evaporated to dryness *in vacuo* and resuspended in 100% methanol. The resulting solution was injected onto a 2 g C-18 cartridge and washed with an eluotropic gradient of 20%, 40%, 60%, and 80% v/v MeOH/H_2_O. The 20% and 40% fractions containing a mixture of borrelidin A reagent and derivatized products were combined and further purified via HPLC to give (**5**) as a light-yellow amorphous solid (2.0 mg; 19% yield; HRMS (*m/z*): [M + H]^+^ calcd. for C_28_H_45_NO_7_, 508.3274; found, 508.3267).

### Synthesis of borrelidin P methyl ester (6)

(Supplementary Fig. 54b) To a RT, stirred solution of **4** (0.93 mg; 0.00183 mmol; 1 eq.) in DCM/MeOH (1:1 *v/v*, 1 mL) was added (trimethylsilyl)diazomethane (2.0 M in hexanes; 22.9 µL; 0.0458 mmol; 25 eq.). The reaction was allowed to proceed for 4 h at RT. After this time, the reaction mixture was evaporated to dryness via a continuous stream of N_2_ gas to yield **6** (1.1 mg; 87%; HRMS (*m/z*): [M + H]^+^ calcd. for C_29_H_47_NO_7_, 522.3431; found, 522.3426) as an off-white powder. The crude product was used immediately in the subsequent reaction.

### Synthesis of linearized borrelidin P methyl diester (7)

(Supplementary Fig. 54c) To a RT, stirred solution of **6** (0.83 mg; 0.00154 mmol; 1 eq.) in MeOH (1 mL) was added NaOMe (0.5 M in MeOH; 25.3 µL; 0.01264 mmol; 8.2 eq.). The reaction was allowed to proceed for 60 h at RT. After this time, the reaction mixture was quenched with a saturated aqueous solution of NH_4_Cl (5 mL). The mixture was transferred to separatory funnel and DCM (5 mL) was added. The organic layer was separated, and the aqueous layer was extracted with DCM (3 × 5 mL). The combined organic extracts were dried (Na_2_SO_4_), filtered, and solvent was removed *in vacuo*. The crude product was purified via HPLC-MS (Phenomenex Kinetix 2.6 µm XB-C18 150 × 4.6 mm) using a gradient of MeCN/H_2_O + 0.02% formic acid (5% MeCN for 5 min, 35% MeCN for 25 min, 100% MeCN for 8 min) at a flow rate of 1.5 mL min^−1^. The appropriate fractions were pooled and solvent removed *in vacuo* to yield **7** (0.49 mg; 50% yield; HRMS (*m/z*): [M + H]^+^ calcd. for C_30_H_51_NO_8_, 554.3693; found, 554.3693) as an off-white solid.

### (*R*)-MTPA-Cl conjugation of linearized borrelidin P methyl diester (8)

(Supplementary Fig. 55) Compound **7** (0.49 mg; 0.00089 mmol; 1 eq.) was dissolved in pyridine-d_5_ (0.5 mL) in a 5 mm NMR tube, and (*R*)-MTPA-Cl (24.8 µL; 0.1328 mmol; 150 eq.) was added via micro-syringe. The reaction was monitored via ^1^H-NMR. After 15 min, ^1^H-NMR showed complete conversion of the starting material to product **8**. After this time, the reaction mixture was evaporated to dryness under a continuous stream of N_2_ gas. The crude product was dissolved in DCM (5 mL) and a saturated aqueous solution of NaHCO_3_ (5 mL) was added. The mixture was transferred to separatory funnel, and the organic layer separated. The aqueous layer was extracted with DCM (3 × 5 mL). The combined organic extracts were dried (Na_2_SO_4_), filtered, and solvent was removed *in vacuo* to yield crude product **8** (0.38 mg; 78% yield; HRMS (*m/z*): [M + Na]^+^ calcd. for C_60_H_72_F_9_NNaO_14_, 1224.4701; found, 1224.4750) as an off-white solid which was used for ^1^H-NMR variable temperature modified Mosher’s ester analysis.

### Collateral sensitivity profiling and MOA investigation of borrelidin analogs

WT *E. coli*, Cef6, and Cef7 mutant strains were transformed with a *thrS* expression vector from the ASKA collection^57^ as follows. Plasmid extractions from overnight cultures of the ASKA *thrS* clone were prepared using the Thermo Scientific GeneJET Plasmid Miniprep Kit. WT, Cef6 and Cef7 overnight cultures were grown in LB Broth, and 100 μL of each strain were spread and incubated on LB agar for 3 h at 37 °C. Cells were scraped from the LB agar plates and vortexed with 1 mL ice-cold 100 mM CaCl_2_ and incubated on ice for 30 min. A 100 μL aliquot of cells was gently mixed with 5 μL of plasmid extract and incubated on ice for 30 min. The transformant solution was heat shocked at 42 °C for 30 s, then again incubated on ice for 2 min. 1 mL of LB was then added to the sample, and incubated at 37 °C, with shaking (150 rpm) for 2 h. A 100 μL aliquot was plated to selective LB agar containing 92.8 μM chloramphenicol. Individual colonies were picked after 24 h of growth at 37 °C, and plasmid extractions were performed to confirm the presence of the *thrS* plasmid. Pure samples of **1** and **4** were prepared as 1:1 dilution series (borrelidin A: 256 μM to ~7.8 nM; borrelidin P: 128 μM to ~3.9 nM) in DMSO and reformatted onto a 384-well plate. Both compounds were screened in triplicate against WT *E. coli* and Cef6, Cef7, RK2, WT-*(thrS)*, Cef6-*(thrS)*, Cef7-*(thrS)* mutant strains according to the protocol described for the antimicrobial control panel. For the (thrS)-transformed mutants, one set of diluted cultures were dispensed normally while a second set was treated with 0.1 mM IPTG inducer 1 h prior to dispensation. Resultant growth data was processed and normalized to controls, then visualized as Hill slope curves using GraphPad Prism.

### Two-dimensional checkerboard assays

Ceftazidime, **1**, and ampicillin were prepared as 15 two-fold dilution series on three separate compound plates: **1** was arranged in a horizontal gradient across plate one (col. 3–17; 128–0 μM), then replicated vertically for 15 rows (row A–O); ceftazidime and ampicillin were arranged in vertical gradients across plates two and three, respectively (row A–O; 128–0 μM), then replicated horizontally for 15 columns (col. 3–17). Plates one-two and one-three were screened in combination against WT, Cef6, and Cef7 strains using the protocol described above, with the liquid handling procedure modified to pin two compound plates into each assay plate. Resulting growth data was normalized as percent growth values and visualized as two-dimensional heatmaps. The FIC index was calculated by selecting the lowest combination of concentrations for each compound producing at least 50% inhibition in growth. Values less than 0.5 are considered synergistic; values in-between 0.5 and 4 are additive/indifferent, and values greater than 4 are antagonistic.

### Intracellular accumulation of borrelidin

Borrelidin accumulation assays were conducted following the protocol from Richer et al.^58^ using Cef6 and Cef7, with WT *E. coli* as a control. Overnight cultures of each bacterium were grown in LB Miller Broth, and aliquoted into 0.25 mL samples. Five replicates (*n* = 5) were prepared for each bacterial strain per drug condition (no drug, 4 μM **1**, and 4 μM mupirocin), although one replicate was lost for the Cef6 intracellular borrelidin treatment (*n* = 4). Each sample was pelleted and washed in 1 mL 1x phosphate buffered saline (PBS) (0.137 M sodium chloride, 0.0027 M potassium chloride, 0.01 M sodium phosphate dibasic, 0.0018 M potassium phosphate monobasic), centrifuged, and resuspended in 0.5 mL 1x PBS. All samples were then equilibrated at 37 °C with shaking (150 rpm) for 5 min. 0.5 mL of 1x PBS containing 8 μM of either **1** or mupirocin was added to each sample for a final drug concentration of 4 μM; 0.5 mL 1x PBS with no drug was used for control samples. Vials were allowed to stand for 10 min at room temperature, then pelleted by centrifugation (16,200 × *g* for 5 min), with the supernatant (SPNT 1) removed and kept. Pellets were washed in 0.5 mL 1x PBS, centrifuged, and the second supernatant (SPNT 2) was removed and pooled with the first supernatant. Cell pellets were resuspended in 210 μL sterile milliQ dH_2_O, then lysed through a series of freeze/thaws, consisting of 10 cycles of submersion in liquid nitrogen for 3 min, followed by 3 min in a 65 °C water bath. After cell lysis, 5 μL was removed from each sample for spot plating onto LB agar to ensure the absence of cell growth. The lysate was collected by centrifugation (16,200 × *g* for 5 min), removing the supernatant (SPNT 3), resuspending samples in 100 μL sterile methanol, centrifuging again, and removing the final supernatant (SPNT 4) and combining with SPNT 3. Samples were stored at −20 °C before MS analysis.

Quantification of target molecule internalization was achieved by liquid chromatography mass spectrometry (LC-MS) using MRM. RP-HPLC was performed using a Waters CORTECS C_8_ 2.7 μm, 2.1 × 50 mm column. Solvent A: 5 mM ammonium acetate, Solvent B: acetonitrile. The gradient was 15% Solvent B for 0.5 min, 15–50% solvent B over 2 min, 50–95% solvent B over 0.5 min, held at 95% solvent B for 1.5 min, 95–15% solvent B over 0.5 min, and finally re-equilibrated at 15% solvent B for 3 min. **1** and mupirocin concentrations were determined using two period MRM on a SCIEX QTrap 5500 instrument. Calibration curves were prepared with a range of 0.003 to 5 μM. For **1**, the transition 488.3 (Q1) > 271.3 (Q3) was monitored at 3.3 min, using a collision energy of −25 V^59^. For mupirocin, the transition 501.1 (Q1) > 327.1 (Q3) was monitored at 2.6 min, using a collision energy of 17 V^60^. MS data were acquired using Analyst v1.6.2 and peak areas were integrated using Skyline v21.1.0.146. **1** and mupirocin concentrations were determined with a linear regression using a (1/concentration) weighting.

### Borrelidin A co-dosing and ceftazidime resistance sequential passaging

An overnight culture of WT *E. coli* K-12 MG1655 was used to inoculate four separate treatment conditions: treatment A, increasing concentrations of ceftazidime starting at 0.063 μM; treatment B, treatment A plus a constant concentration of **1** at 32 μM; treatment C, treatment A plus a constant concentration of **1** at 128 μM; and treatment D, treatment A with increasing concentrations of norfloxacin starting at 0.016 μM. All treatments are performed in triplicate in 96- well plates with 200 μL of LB media. Four concentrations were tested for each treatment, increasing two-fold from the starting concentration (i.e., treatment A: 0.063 μM, 0.125 μM, 0.25 μM, and 0.5 μM). Post-treatment and inoculation, plates were incubated at 37 °C, 5% CO_2_ for 24 h before an absorbance reading at OD_600_ is taken. The highest drug concentration resulting in at least 50% growth inhibition for each replicate was used to inoculate the subsequent day’s treatment wells at a 1:100 ratio. Ceftazidime and norfloxacin drug concentration gradients were also modified in 2-fold steps as required to ensure that at least one concentration that produced 100% growth. Borrelidin A treatments were maintained at fixed concentrations. Resistance monitoring/sequential passaging was repeated daily for 12 consecutive days, the maximum length of time achievable given the high rate of consumption of a limited NP compound. The difference between day 1 and day 12 MIC values for each passaging condition was analyzed by a one-way ANOVA with Tukey’s HSD test. At the conclusion of the experiment, the most resistant samples from each replicate were archived as glycerol stocks at −70 °C. To determine the MIC for each resistant strain under standard conditions, glycerol stocks were thawed and used to inoculate overnight cultures. Ceftazidime, cefadroxil, cefaclor, ciprofloxacin, norfloxacin, and borrelidin A were prepared as 1:1 dilution series (128 μM to ~3.9 nM) in DMSO and reformatted into a 384-well plate. All compounds were screened against the ceftazidime-resistant strains following standard procedures described above and their MICs determined for each resistant replicate.

### Reporting summary

Further information on research design is available in the Nature Portfolio Reporting Summary linked to this article.

## Supplementary information

Supplementary Information

Peer Review File

Reporting Summary

## Data Availability

The Genome sequence data for all drug resistant *E. coli* mutants generated in this study have been deposited in the NCBI database under accession code PRJNA932790. The NMR data for compounds **1**-**4** have been deposited in the Natural Products Magnetic Resonance Database under accession numbers NP0331529, NP0017652, NP0017654, NP0331530. The raw data from primary and secondary screens, as well as checkerboard assays and resistance passaging have been deposited in Zenodo [10.5281/zenodo.7552218]^61^. Source data are provided with this paper.

## Source data

Source data
