# Supplementary material for: Collateral sensitivity profiling in drug-resistant Escherichia coli identifies natural products suppressing cephalosporin resistance
Source: Nat Commun. 2023 Apr 8;14:1976. doi: 10.1038/s41467-023-37624-4 (PMC10082850; doi:10.1038/s41467-023-37624-4)
Supplement: Supplementary file 1 — Supplementary Information [file 41467_2023_37624_MOESM1_ESM.pdf]

SUPPLEMENTARY INFORMATION

Collateral Sensitivity Profiling in Drug-Resistant *Escherichia coli* Identifies Natural Products Suppressing Cephalosporin Resistance

Dennis Y. Liu[1], Laura Phillips[2], Darryl M. Wilson[1], Kelly M. Fulton[3], Susan M. Twine[2,3], Alex Wong[2], Roger G. Linington[1,*]

[1] Department of Chemistry, Simon Fraser University, 8888 University Dr., V5A 1S6 Burnaby, BC Canada

[2] Department of Biology, Carleton University, 1125 Colonel By Dr., K1S 5B6 Ottawa, ON Canada

[3] Human Health Therapeutics Research Center, National Research Council Canada, 100 Sussex Dr., K1N 5A2 Ottawa, ON Canada

[4] Institute for Advancing Health Through Agriculture, Texas A&M AgriLife, 1500 Research Parkway, 77845 College Station, TX United States

* Corresponding Author

Email: rliningt@sfu.ca

Table of Contents

Supplementary Note 1 Structure Elucidation Details for Borrelidin P (4) 5

Supplementary Fig. 1 (Part I) Collateral-sensitivity profiling of 80 commercial antimicrobials against a drug-resistant *E. coli* target panel. 7

Supplementary Fig. 1 (Part II) Collateral-sensitivity profiling of 80 commercial antimicrobials against a drug-resistant *E. coli* target panel. 8

Supplementary Fig. 2 (Part I) Secondary screening of 120 hit extracts in dilution series against the target panel. 9

Supplementary Fig. 2 (Part II) Secondary screening of 120 hit extracts in dilution series against the target panel. 10

Supplementary Fig. 2 (Part III) Secondary screening of 120 hit extracts in dilution series against the target panel. 11

Supplementary Fig. 3 CS activity profiles of borrelidins A (1), F (2), and H (3). 12

Supplementary Fig. 4 Borrelidin A (1) is not synergistic with ceftazidime. 13

Supplementary Fig. 5 Intracellular concentrations of 1 is not affected by cephalosporin-resistant cell wall biosynthesis mutations in strains Cef6 and Cef7. 14

Supplementary Fig. 6 Ceftazidime MIC values as determined through 12 days of serial passaging wildtype *E. coli* under different drug conditions. 15

Supplementary Fig. 7 HRMS of borrelidin A (1). 16

Supplementary Fig. 8 ^{1}H-NMR spectrum of borrelidin A (1) at 600 MHz in CD_3OD 17

Supplementary Fig. 9 ^{13}C-NMR spectrum of borrelidin A (1) at 150 MHz in CD_3OD 18

Supplementary Fig. 10 HRMS of borrelidin F (2). 19

Supplementary Fig. 11 ^{1}H-NMR spectrum of borrelidin F (2) at 600 MHz in CD_3OD 20

Supplementary Fig. 12 ^{1}H-NMR spectrum of borrelidin F (2) at 600 MHz in DMSO-d_6 21

Supplementary Fig. 13 gCOSY spectrum of borrelidin F (2) at 600 MHz in DMSO-d_6 22

Supplementary Fig. 14 gHSQC spectrum of borrelidin F (2) at 600 MHz in DMSO-d_6 23

Supplementary Fig. 15 HRMS of borrelidin H (3). 24

Supplementary Fig. 16 ^{1}H-NMR spectrum of borrelidin H (3) at 600 MHz in CD_3OD 25

Supplementary Fig. 17 ^{1}H-NMR spectrum of borrelidin H (3) at 600 MHz in DMSO-d_6 26

Supplementary Fig. 18 gCOSY spectrum of borrelidin H (3) at 600 MHz in DMSO-d_6 27

Supplementary Fig. 19 gHSQC spectrum of borrelidin H (3) at 600 MHz in DMSO-d_6 28

Supplementary Fig. 20 HRMS of borrelidin P (4). 29

Supplementary Fig. 21 ^{1}H-NMR spectrum of borrelidin P (4) at 600 MHz in CD_3OD 30

Supplementary Fig. 22 ^{13}C-NMR spectrum of borrelidin P (4) at 150 MHz in CD_3OD 31

Supplementary Fig. 23 gCOSY spectrum of borrelidin P (4) at 600 MHz in CD3OD 32

Supplementary Fig. 24 gHSQC spectrum of borrelidin P (4) at 600 MHz in CD_3OD 33

Supplementary Fig. 25 gHMBC spectrum of borrelidin P (4) at 600 MHz in CD_3OD 34

Supplementary Fig. 26 NOESY spectrum of borrelidin P (4) at 600 MHz in CD_3OD 35

Supplementary Fig. 27 ^{1}H-NMR spectrum of borrelidin P (4) at 600 MHz in DMSO-d_6 36

Supplementary Fig. 28 gCOSY spectrum of borrelidin P (4) at 600 MHz in DMSO-d_6...........37

Supplementary Fig. 29 gHSQC spectrum of borrelidin P (4) at 600 MHz in DMSO-d_6 38

Supplementary Fig. 30 gHMBC spectrum of borrelidin P (4) at 600 MHz in DMSO-d_6..........39

Supplementary Fig. 31 Key 2D-NMR correlations of compound 4 in methanol-d_4 at 600 MHz and 150 MHz for ^{1}H and ^{13}C, respectively..40

Supplementary Fig. 32 HRMS of 12-desnitrile-12-carbamoyl-borrelidin A (5).......................41

Supplementary Fig. 33 ^{1}H-NMR spectrum of 12-desnitrile-12-carbamoyl-borrelidin A (5) at 600 MHz in CD_3OD 42

Supplementary Fig. 34 HRMS of borrelidin P methyl ester (6)..43

Supplementary Fig. 35 ^{1}H-NMR spectrum of borrelidin P methyl ester (6) at 600 MHz in pyridine-d_5..44

Supplementary Fig. 36 ^{13}C-NMR spectrum of borrelidin P methyl ester (6) at 150 MHz in pyridine-d_5..45

Supplementary Fig. 37 gCOSY spectrum of borrelidin P methyl ester (6) at 600 MHz in pyridine-d_5..46

Supplementary Fig. 38 HSQC spectrum of borrelidin P methyl ester (6) at 600 MHz in pyridine-d_5..47

Supplementary Fig. 39 HMBC spectrum of borrelidin P methyl ester (6) at 600 MHz in pyridine-d_5..48

Supplementary Fig. 40 NOESY spectrum of borrelidin P methyl ester (6) at 600 MHz in pyridine-d_5..49

Supplementary Fig. 41 HRMS of linearized borrelidin P methyl diester (7)...........................50

Supplementary Fig. 42 ^{1}H-NMR s spectrum of linearized borrelidin P methyl diester (7) at 600 MHz in pyridine-d_5 51

Supplementary Fig. 43 gCOSY spectrum of linearized borrelidin P methyl diester (7) at 600 MHz in pyridine-d_5 52

Supplementary Fig. 44 gHSQC spectrum of linearized borrelidin P methyl diester (7) at 600 MHz in pyridine-d_5 53

Supplementary Fig. 45 gHMBC spectrum of linearized borrelidin P methyl diester (7) at 600 MHz in pyridine-d_5 54

Supplementary Fig. 46 HRMS of linearized borrelidin P methyl diester (*S*)-MTPA (8)..........55

Supplementary Fig. 47 ^{1}H-NMR spectrum of linearized borrelidin P methyl diester (*S*)-MTPA (8) at 25 ºC, 600 MHz in pyridine-d_5...56

Supplementary Fig. 48 gCOSY spectrum of linearized borrelidin P methyl diester (*S*)-MTPA (8) at 25 ºC, 600 MHz in pyridine-d_5...57

Supplementary Fig. 49 gHSQC spectrum of linearized borrelidin P methyl diester (*S*)-MTPA (8) at 25 ºC, 600 MHz in pyridine-d_5...58

Supplementary Fig. 50 ^{1}H-NMR spectrum of linearized borrelidin P methyl diester (*S*)-MTPA (8) at 5 ºC, 600 MHz in pyridine-d_5...59

Supplementary Fig. 51 gCOSY spectrum of linearized borrelidin P methyl diester (*S*)-MTPA (8) at 5 ºC, 600 MHz in pyridine-d_5...60

Supplementary Fig. 52 gHSQC spectrum of linearized borrelidin P methyl diester (*S*)-MTPA (8) at 5 ºC, 600 MHz in pyridine-d_5...61

Supplementary Fig. 53 Derivatization of 12-desnitrile-12-carbamoyl-borrelidin A (5) from borrelidin A (1) **62**

Supplementary Fig. 54 Borrelidin P esterification (6) and base-catalyzed ring opening (7). **63**

Supplementary Fig. 55 Linearized borrelidin P methyl ester (7) modified Mosher's ester conjugation (8). **64**

Supplementary Table 1. Collateral Sensitivity Profiling target panel consists of 29 unique drug-resistant *E. coli* strains. **65**

Supplementary Table 2. 80-member antimicrobial control library. **66**

Supplementary Table 3. Z'-factor and Z-factor values for CSP screening of the antimicrobial library and the natural product library, respectively. **69**

Supplementary Table 4. Tabulated NMR data for (1) and (4) in methanol-d_4 at 600 MHz and 150 MHz for ^{1}H- and ^{13}C-NMR, respectively **70**

Supplementary Table 5. Tabulated ^{1}H-NMR shifts for linearized Borrelidin P methyl ester (7) and (S)-MTPA conjugated product (8) under two different temperatures at 600 MHz in pyridine-d_5. .... **72**

Supplementary References **73**

Supplementary Note 1 Structure Elucidation Details for Borrelidin P (**4**)

Borrelidin P (**4**) was isolated alongside the known compound Borrelidin A (**1**; **Supplementary Fig. 7 – 9**), which served as a structural template for the elucidation of **4**. The molecular formula of **4** was determined through HRMS as $C_{28}H_{45}NO_7$, which differs from the molecular formula of **1** by the addition of H_2O. The structure of **4** was solved by NMR analysis using a combination of ^{1}H, ^{13}C, gCOSY, gHSQC, gHMBC, and NOESY spectra (**Supplementary Table 4; Supplementary Fig. 20 – 30**). First, comparisons of the ^{1}H-NMR spectra between **1** and **4** in DMSO-d_6 showed close agreement in the chemical shifts of key signals. However, **4** presented an additional set of doublets at δ_H 7.18, integrating for 2. Close examination of the gHSQC spectrum showed no corresponding carbon signal for this doublet, while long-distance gHMBC correlations extending from the nearest olefin at δ_H 6.01 point to the presence of a new carbon signal at δ_C 169.2. Together these data indicated that the nitrile functional group in **1** had been replaced with a primary amide in **4**. This structure satisfies both the pre-determined molecular formula and the 7 degrees of unsaturation (**Supplementary Fig. 31**).

Further elucidation of **4**'s 2D planar structure proceeded in methanol-d_4, which provided improved resolution in the overlapping region of aliphatic signals. Interpretation of the gCOSY spectrum determined that **4** was divided into two spin systems, A and B. Spin system A contained three olefinic signals, four pairs of diastereotopic protons, and an oxymethine at δ_H 5.06 (t) based on ^{1}H-NMR and gHSQC spectra. Using the gCOSY spectrum, spin system A was assigned starting from the olefin at δ_H 6.19 (d), followed by two adjacent olefins, one pair of diastereotopic protons, and finally to the oxymethine. Continuing onwards in the gCOSY spectrum revealed a methine at δ_H 2.53 (obscured beneath the residual water signal in the ^{1}H-NMR spectrum) and then three sequential pairs of diastereotopic protons. Comparisons between **1** and **4** indicated that this region is a pendant 5-membered ring adjacent to the oxymethine. The last methine in the ring is not observed in our analysis, but long-range gCOSY signals from the δ_H 2.53 methine proved that the substructure was cyclized. Furthermore, gHMBC correlations extending from one of two diastereotopic protons at δ_H 1.37 to a carbonyl signal at δ_C 183.6, indicated the presence of a terminal carboxylic acid motif, which is a common feature for borrelidin congeners.

Spin system B contained two oxymethines, four terminal methyl groups, and four pairs of diastereotopic protons. Through a combination of gCOSY, gHSQC, and gHMBC data, spin system B was assigned in a similar manner as spin system A starting from the two oxymethines at δ_H 3.74 (d) and δ_H 3.85 (q). Analysis of the data showed that between the two oxymethines are four repeating pairs of -CH(CH_3)-CH_2- subunits with heavily overlapped signals. gCOSY correlations from the oxymethine at δ_H 3.85 pointed to a pair of methylene protons δ_H 2.4, integrating for 2, while gHMBC correlations revealed a nearby carbonyl at δ_C 174.7. Spin systems A and B were pieced together using HMBC correlations from the terminal ends of both systems. The olefinic group of spin system A at δ_H 6.19 was connected to a quaternary carbon at δ_C 135.5 along with the oxymethine signal δ_H 3.74 of spin system B and the newly identified primary amide. The downfield oxymethine at δ_H 5.06 was connected to the carbonyl at δ_C 174.7 via an ester linkage, thereby completing the macrolactone ring.

Comparisons between **4** and **5** (12-desnitrile-12-carbamoyl-borrelidin A; identical planar structure; **Supplementary Fig. 33 – 34**, **53**) revealed key differences, namely the upfield shift of the olefinic proton at C13 of **4**. Close examination of the NOESY spectra pointed to a unique through space correlation in **4** between the olefin at C13 and the oxymethine at C11, which indicated a 12-(*Z*) configuration across the double bond in contrast to the 12-(*E*) configuration in **5**. Alternating NOESY correlations between C13 to C15 and C14 to C16 is identical to those found in **1** and indicated that the second double bond is 14-(*E*). Initial efforts to deduce the absolute stereochemistry of the secondary alcohols on an intact sample of **4** using Mosher's esters were unsuccessful, likely due to steric hindrance preventing efficient Mosher's ester formation. To resolve this issue the free carboxylic acid of **4** was first protected via esterification to form **6**, before the macrolactone ring was opened via a base-mediated reaction (**7**; **Supplementary Fig. 54**). The 3-(*S*)-11-(*R*) configurations of **4**'s two secondary alcohols were subsequently determined from (**8**; **Supplementary Fig. 55**;

Supplementary Table 5) using the variable temperature Mosher's ester method described by Latypov *et al.*[1].

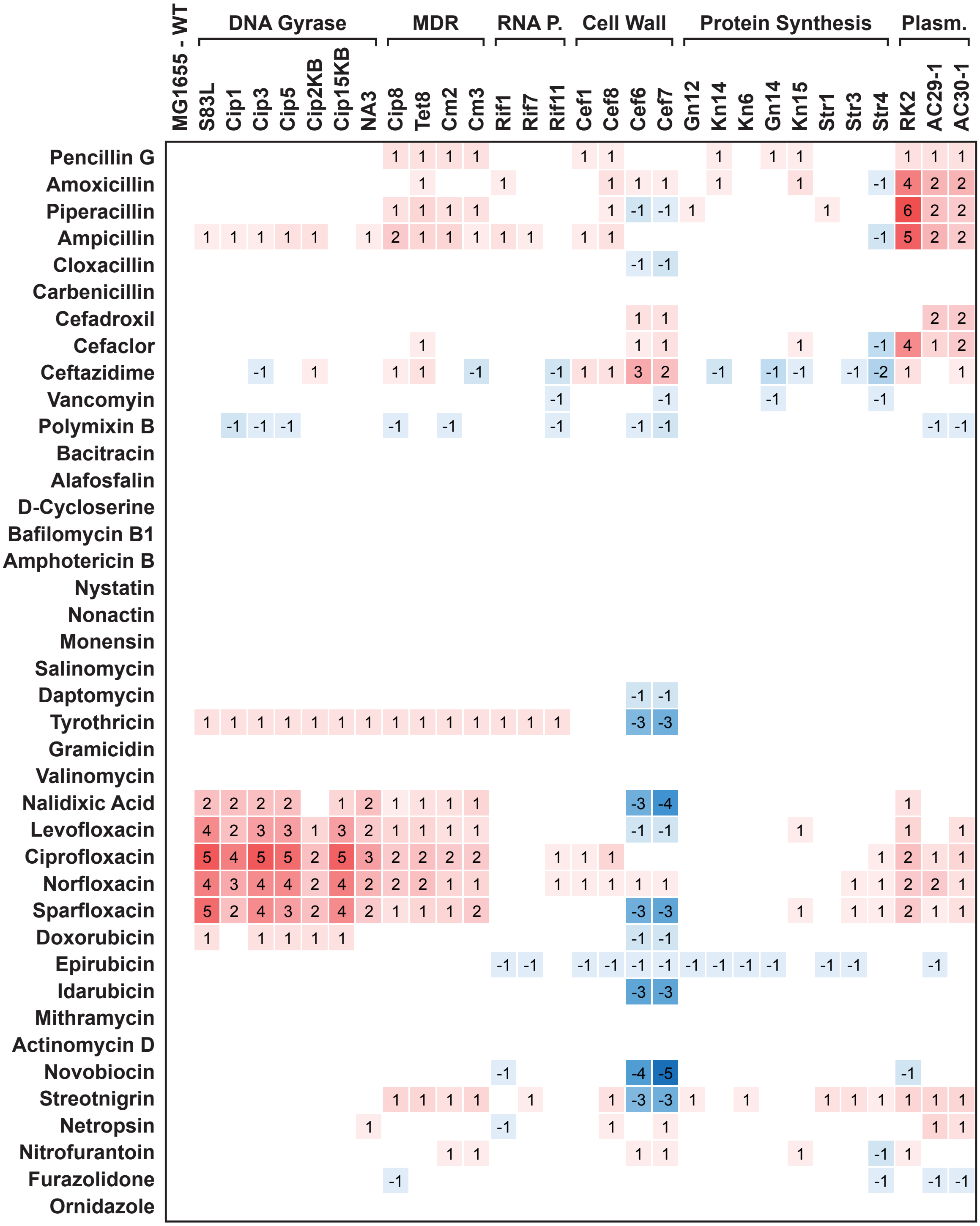

Supplementary Fig. 1 (Part I) Collateral-sensitivity profiling of 80 commercial antimicrobials against a drug-resistant *E. coli* target panel. Legend: white = no change in Log_2MIC compared to wildtype; red = increase in Log_2MIC compared to wildtype by *n* (resistance); blue = decrease in Log_2MIC compared to wildtype by *n* (collateral sensitivity). Mutant *E. coli* strains are categorized by their resistance mechanism (top). MDR = multidrug resistance; RNA P. = RNA polymerase; Plasm. = plasmid-borne multi-drug resistance. Source data are provided as a Source Data file.

		DNA Gyrase							MDR				RNA P.			Cell Wall				Protein Synthesis								Plasm.		
	MG1655 - WT	S83L	Cip1	Cip3	Cip5	Cip2KB	Cip15KB	NA3	Cip8	Tet8	Cm2	Cm3	Rif1	Rif7	Rif11	Cef1	Cef8	Cef6	Cef7	Gn12	Kn14	Kn6	Gn14	Kn15	Str1	Str3	Str4	RK2	AC29-1	AC30-1
Sulfamethazine																														
Sulfapyridine																														
Sulfamethoxazole																														
Sulfadiazine																														
Sulfamerazine																														
Rifampicin										1			4	4	4	1	1	-2	-2									1		1
Rifabutin									1	1	1	1	4	4	5	1	1	-3	-3		1	1						1	1	1
Rifaximin						1		1	1	1	1	1	3	3	3		1	-1	-2				1					1	1	1
Resistomycin																														
Resistoflavine																														
Oxytetracycline				-1		-1	-1		1	1										-1			-1			-1	-1	4		
Tetracycline									2	2	1	1				1	1										-1	7		
Doxycycline									1	2	2	2					1	-2	-2								-1	4		
Demeclocycline									1	1	1	1					1	-1	-1				-1			-1	-1	8	1	
Minocycline									2	2	1	1						-1	-2			1		1		1		3		
Tobramycin						-1										-1	-1							1	-1					
Gentamicin																		-1	-1		-1				-1			-1		
Kanamycin									-1				-1		-1	-1		-1	-1	-1		-1	1		-1	1	1	2	1	1
Amikacin		-1					-1		-1			-1	-1			-1	-1		-1		1		1	1		1	1			
Streptomycin		1		1	1	1	1	1			1										1		1		2	2	2			
Spectinomycin																														
Erythromycin																		-2	-3											
Clarithromycin																		-1	-2											
Midecamycin																		-1	-3											
Azithromycin									1	1	1	1						-2	-3										1	1
Roxithromycin																														
Spiramycin																														
Thiamphenicol																											-1		-2	-2
Chloramphenicol							-1		1	1			-1	-1				-1	-2	-1	-1	-1	-1		-1	-1	-1		-1	-1
Florfenicol				-1	-1		-1	-1		1			-1	-1	-1			-1	-1		-1	-1	-1	-1	-1	-1	-2		-1	-1
Clindamycin																		-2	-1											
Lincomycin																														
Cycloheximide																														
Thiostrepton		1	1	1	1	1	1	1	1	1	1	1	1	1	1	1	1									1	1	1	1	1
Puromycin									1	1	1	1		1		1	1		1	1	1	1	1	1	1	1	1	1	1	1
Tiamulin		-1	-2	-2			-1		-1			-1		-1	-1	-2		-2	-2	-1			-1		-1	-1	-1	-1	-1	-1
Staurosporine																			-2	-1		-1	-1							
Antimycin A																														
Tunicamycin																														
Cyclosporin A																														

Supplementary Fig. 1 (Part II) Collateral-sensitivity profiling of 80 commercial antimicrobials against a drug-resistant *E. coli* target panel. Legend: white = no change in Log2MIC compared to wildtype; red = increase in Log2MIC compared to wildtype by *n* (resistance); blue = decrease in Log2MIC compared to wildtype by *n* (collateral sensitivity). Mutant *E. coli* strains are categorized by their resistance mechanism (top). MDR = multidrug resistance; RNA P. = RNA polymerase; Plasm. = plasmid-borne multi-drug resistance. Source data are provided as a Source Data file.

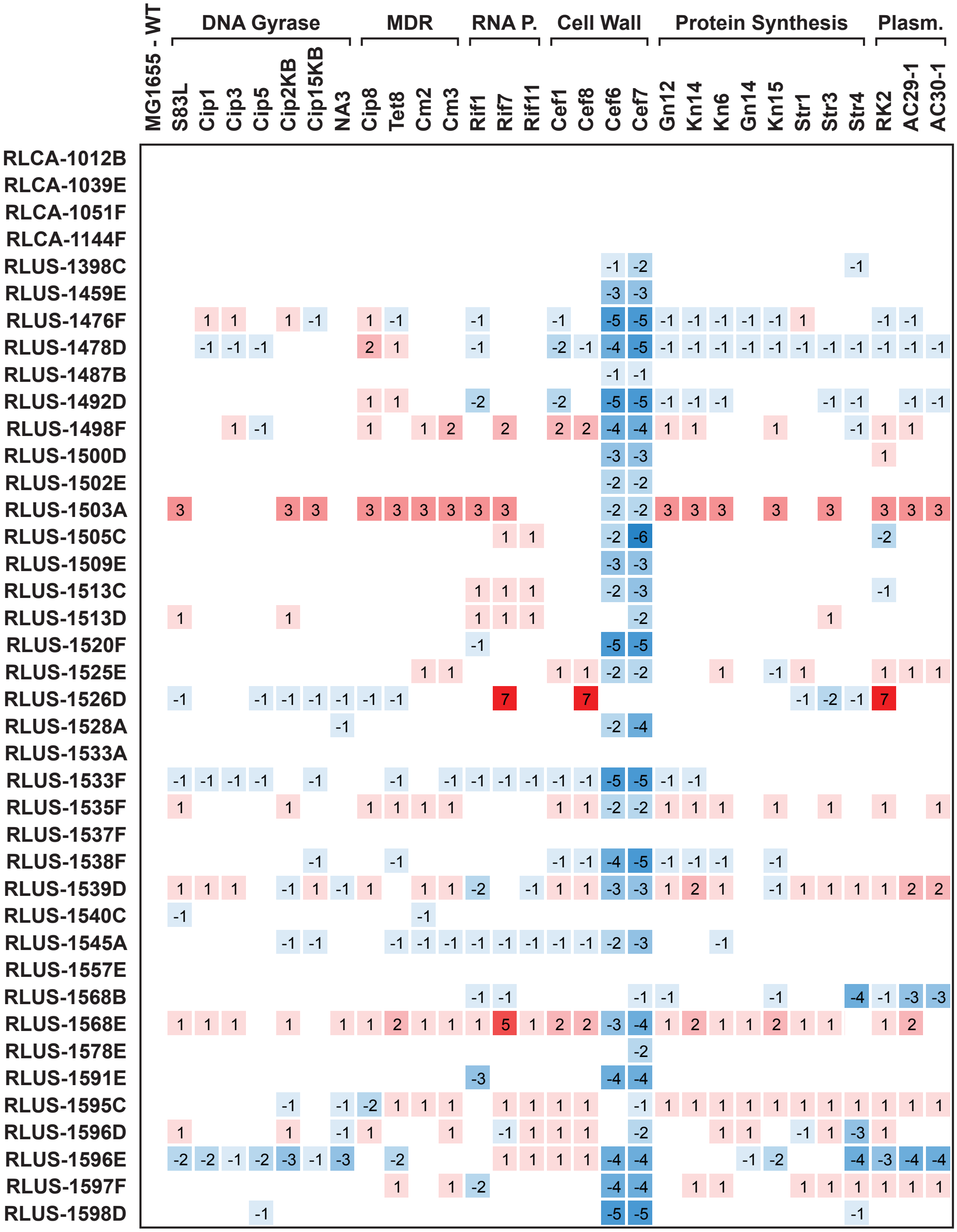

Supplementary Fig. 2 (Part I) Secondary screening of 120 hit extracts in dilution series against the target panel. Legend: white = no change in MIC compared to wildtype; red = increase in Log_2MIC compared to wildtype by *n* (resistance); blue = decrease in Log_2MIC compared to wildtype by *n* (collateral sensitivity). Mutant *E. coli* strains are categorized by their resistance mechanism (top). MDR = multidrug resistance; RNA P. = RNA polymerase; Plasm. = plasmid-borne multi-drug resistance. Source data are provided as a Source Data file.

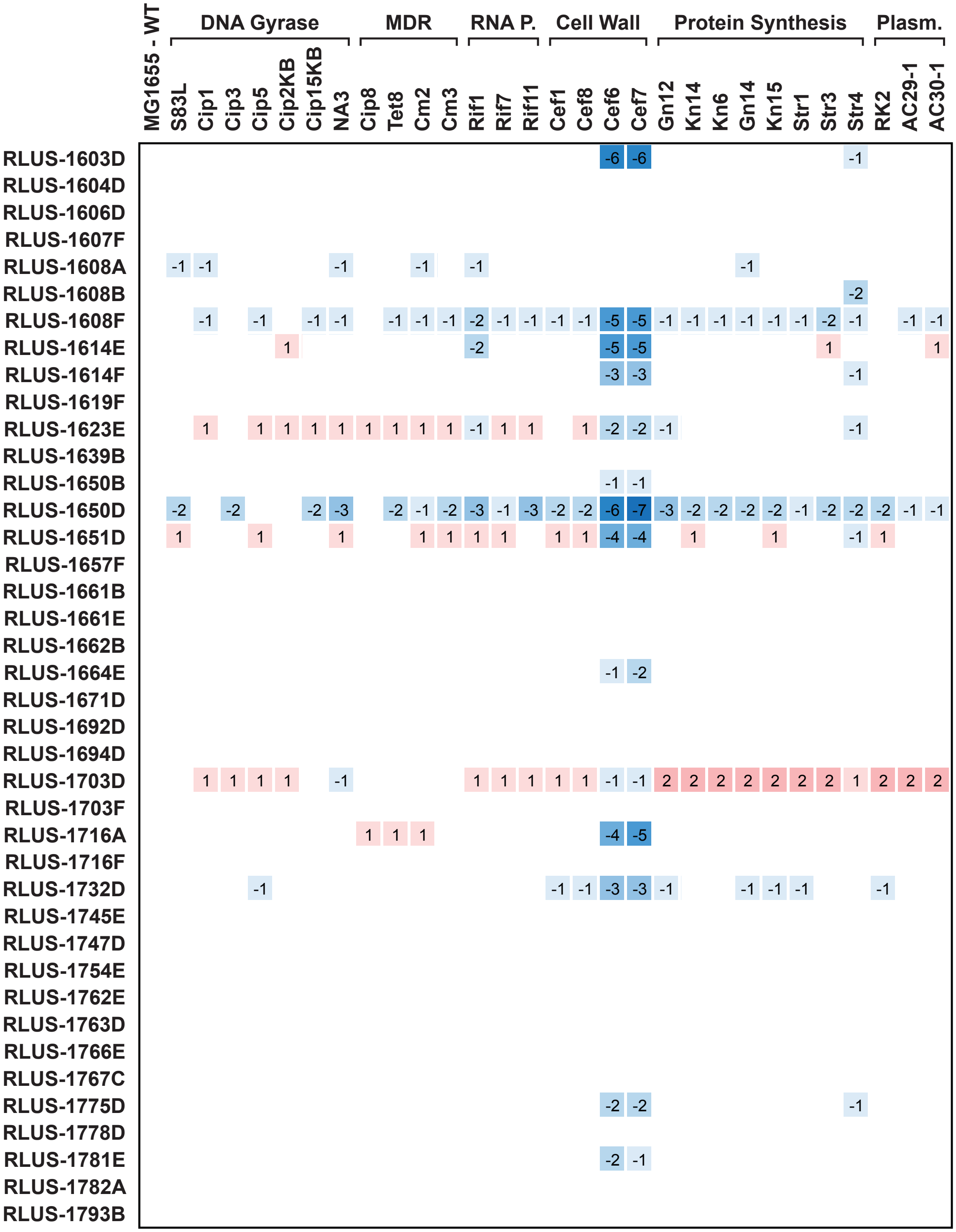

Supplementary Fig. 2 (Part II) Secondary screening of 120 hit extracts in dilution series against the target panel. Legend: white = no change in MIC compared to wildtype; red = increase in Log_2MIC compared to wildtype by *n* (resistance); blue = decrease in Log_2MIC compared to wildtype *by n* (collateral sensitivity). Mutant *E. coli* strains are categorized by their resistance mechanism (top). MDR = multidrug resistance; RNA P. = RNA polymerase; Plasm. = plasmid-borne multi-drug resistance. Source data are provided as a Source Data file.

		DNA Gyrase							MDR				RNA P.			Cell Wall				Protein Synthesis								Plasm.		
	MG1655 - WT	S83L	Cip1	Cip3	Cip5	Cip2KB	Cip15KB	NA3	Cip8	Tet8	Cm2	Cm3	Rif1	Rif7	Rif11	Cef1	Cef8	Cef6	Cef7	Gn12	Kn14	Kn6	Gn14	Kn15	Str1	Str3	Str4	RK2	AC29-1	AC30-1
RLUS-1794B																														
RLUS-1796F			-1	-1	-1	-1		-1	-1									-5	-5											
RLUS-1805D																														
RLUS-1806C																														
RLUS-1811E																														
RLUS-1813E		3		-1		2	-1		2	1	-1	1	1		1	6	6	3	2			2	1	6	-1	2	-1	6	1	2
RLUS-1814E								-2	1	1			-1		1	1	1	-2	-2	1	1	1		-1	1	1		1	1	1
RLUS-1816D																														
RLUS-1833B																														
RLUS-1837A																														
RLUS-1850D																														
RLUS-1852D		1						-1								-1	-1	-3	-4							1				1
RLUS-1859D																														
RLUS-1860C																														
RLUS-1875F																														
RLUS-1878D			-1	-1	-1			-1					-2			-2	-2	-5	-6	-2	-2	-2	-1	-1	-2	-2	-2	-2	-2	-2
RLUS-1938A																														
RLUS-1955E																		-5	-6											
RLUS-1984E																		-4	-4											
RLUS-2000E								-1					-1					-1	-1						1				1	1
RLUS-2005C																														
RLUS-2018D																														
RLUS-2024F			-1	-1	-1	-1							-2			-1	-1	-2	-2	-1	-1		-1	-1	1		-1			
RLUS-2028A																	-1	-3	-4											
RLUS-2828B		1	1	1	1	1	3	1	1	3	1	1	1	1	1			-3	-3	1	2	1	1	3	1	4		1	1	1
RLUS-2028C						1	1	1	2	2	1	1		1	1	-1		-3	-3		1		1	1						1
RLUS-2031F		-2	-2	-2	-2	-2		-2					-3			-2	-2	-4	-5	-2	-2		-2	-2			-2			
RLUS-2035E						-1	1			1	1	3	-1	1	1	1		-1	-1			1	1	1	3	3	1	1	3	3
RLUS-2043E																		-4	-4											
RLUS-2052E		-1		-1	-1	-1		-1	-1				-2				-1	-2	-2						2		-1		1	1
RLUS-2072E								-3										-6	-6											
RLUS-2078F							1		1	1				1	1			-3	-3				1							
RLUS-2096D																														
RLUS-2101D		-3	-3	-3	-3		-3	-3	-2	-3	-3		-3	-3	-3	-3	-3	-6	-6	-3	-3	-3	-3	-3	-3	-3	-3	-3	-3	-3
RLUS-2108F																		-1	-2											
RLUS-2169E																		-3	-3					-1						
RLUS-2213E																														

Supplementary Fig. 2 (Part III) Secondary screening of 120 hit extracts in dilution series against the target panel. Legend: white = no change in Log_2MIC compared to wildtype; red = increase in Log_2MIC compared to wildtype by *n* (resistance); blue = decrease in Log_2MIC compared to wildtype by *n* (collateral sensitivity). Mutant *E. coli* strains are categorized by their resistance mechanism (top). MDR = multidrug resistance; RNA P. = RNA polymerase; Plasm. = plasmid-borne multi-drug resistance. Source data are provided as a Source Data file.

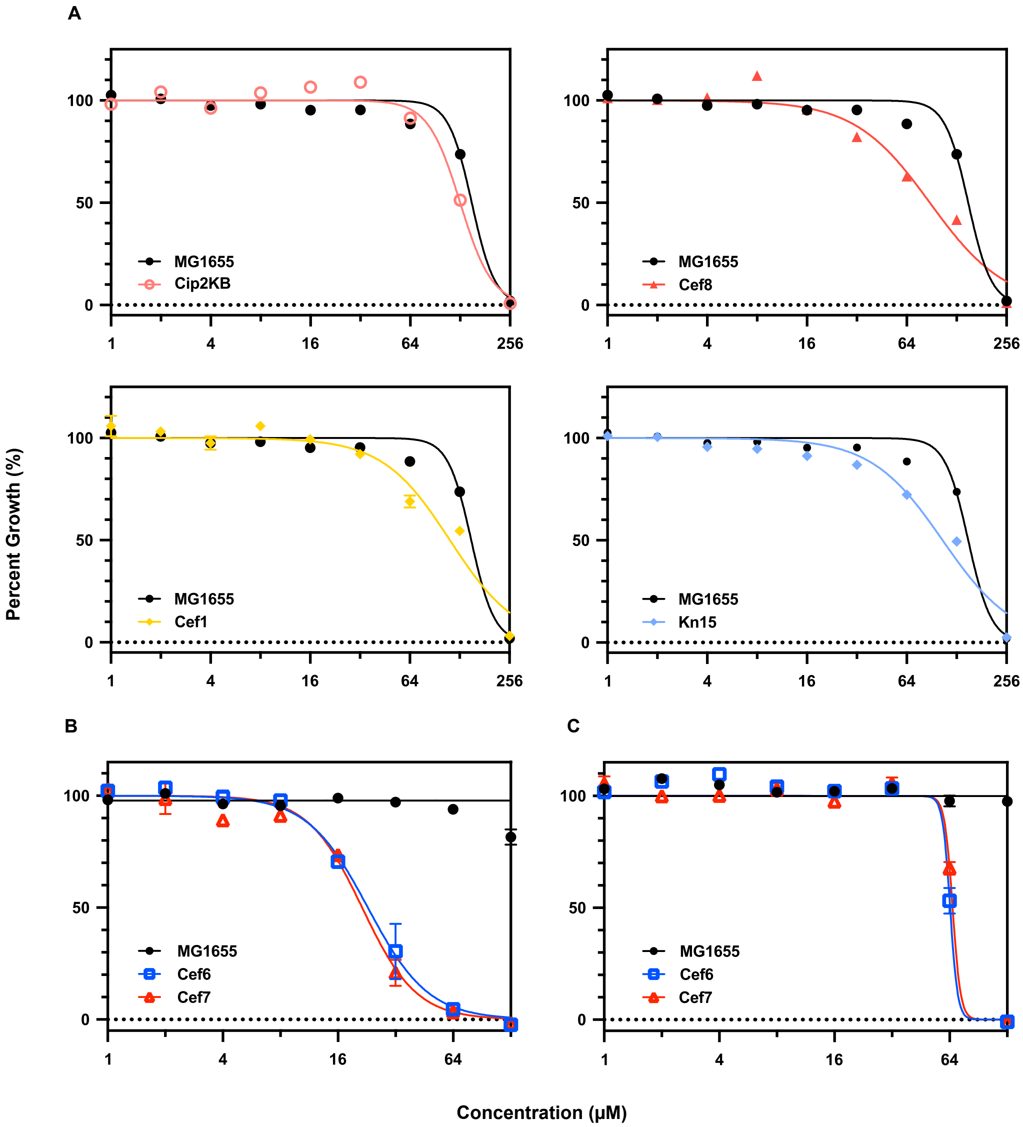

Supplementary Fig. 3 CS activity profiles of borrelidins A (**1**), F (**2**), and H (**3**). (**A**) Dose response curves of WT *E. coli*, Cip2KB (fluoroquinolone-resistant), Cef8 and Cef1 (cephalosporin-resistant), and Kn15 (aminoglycoside-resistant) strains treated with **1**. (**B**) Dose response curves of WT *E. coli*, Cef6, and Cef7 strains treated with **2**. (**C**) Dose response curves of WT *E. coli*, Cef6, and Cef7 strains treated with **3**. Data shows the average of three independent experiments (n = 3); error bars denote the standard error of the mean (SEM). Source data are provided as a Source Data file.

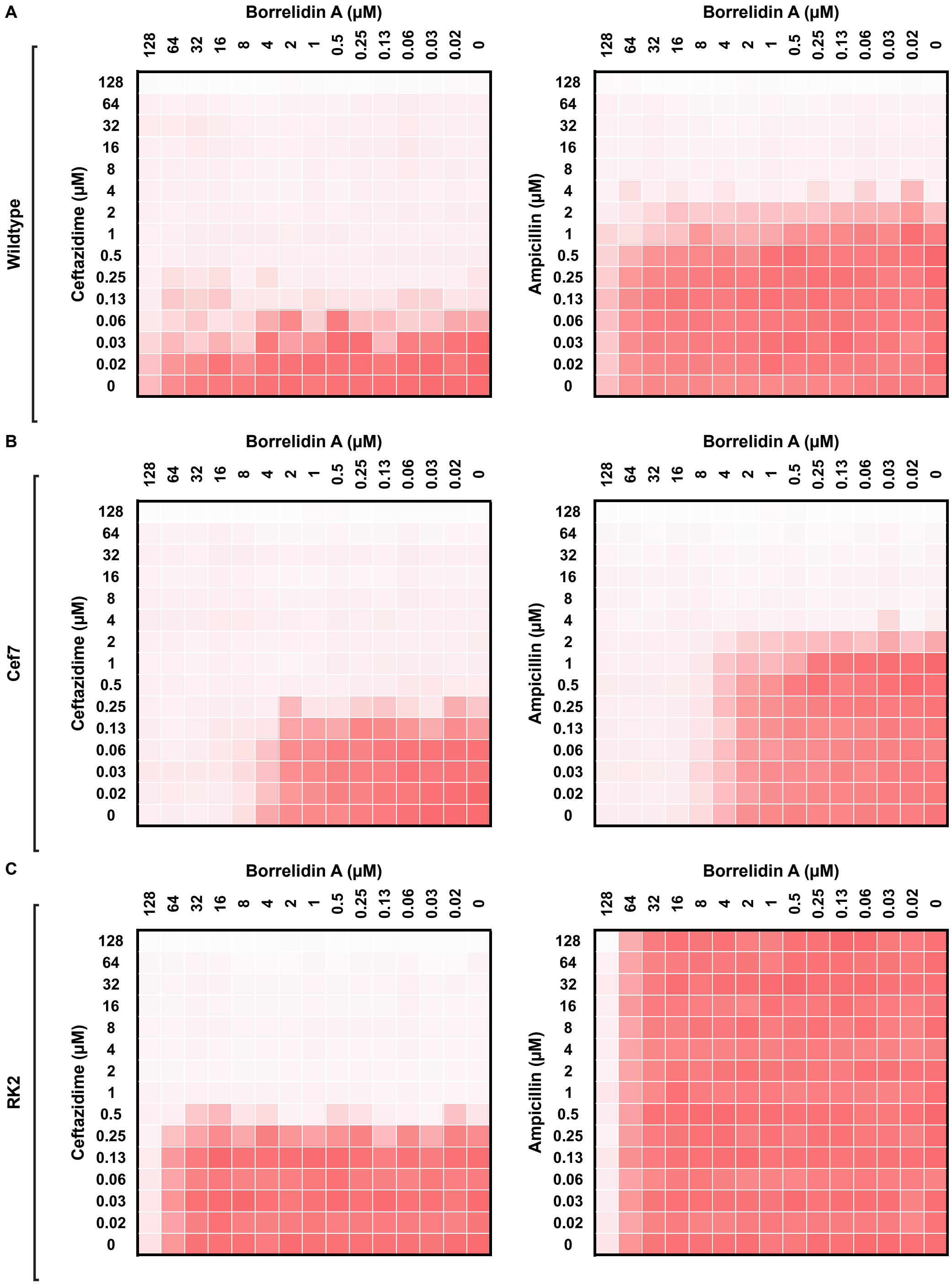

Supplementary Fig. 4 Borrelidin A (**1**) is not synergistic with ceftazidime. Two-dimensional checkerboard assays were performed between **1** and ceftazidime or with ampicillin control for wildtype *E. coli* (**A**), cephalosporin-resistant strain Cef7 (**B**), and plasmid-borne multidrug-resistant strain RK2 (**C**). Each square represents a heatmap of percent growth values assessed at various combinations of compound concentrations; white = no growth, red = growth. Data represents the average of three independent experiments (n = 3). Source data are provided as a Source Data file.

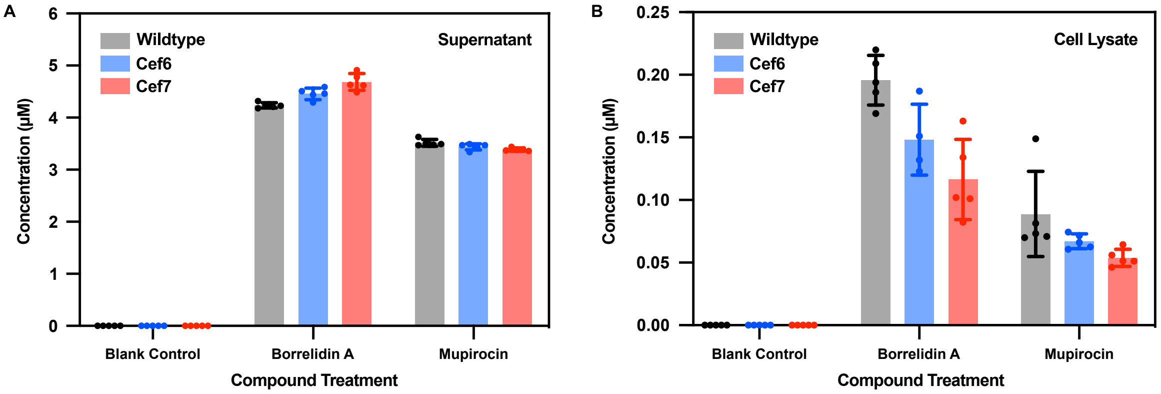

Supplementary Fig. 5 Intracellular concentrations of **1** is not affected by cephalosporin-resistant cell wall biosynthesis mutations in strains Cef6 and Cef7. Targeted quantification by HPLC-HRMS using MRM was performed to determine the intracellular concentrations of **1** in both the Cef6 and Cef7 mutant strains post-incubation, compared to wildtype (WT). The isoleucyl-tRNA synthetase inhibitor mupirocin is used as a control for overall protein synthesis inhibition. (**A**) Average concentrations of mupirocin or **1** in the supernatant fraction post-incubation. (**B**) Average concentrations of mupirocin or **1** in the cell lysate fraction post-incubation. Blank controls: WT, Cef6, and Cef7 strains were incubated in the absence of **1** or mupirocin. Bar charts display the average of five independent experiments; error bars denote standard deviation. Source data are provided as a Source Data file.

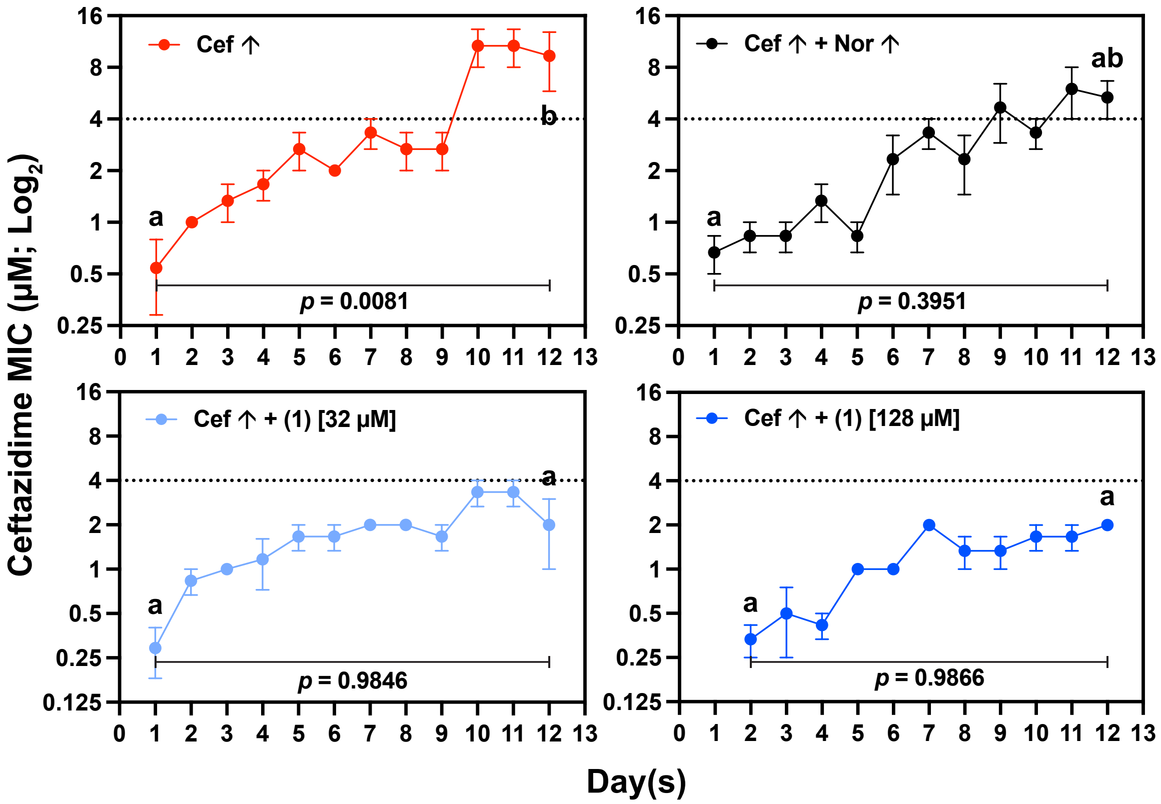

Supplementary Fig. 6 Ceftazidime MIC values as determined through 12 days of serial passaging wildtype *E. coli* under different drug conditions. Cef = ceftazidime; Nor = norfloxacin, (**1**) = borrelidin A. Dotted line represents the EUCAST breakpoint for ceftazidime resistance in *E. coli*. ↑ represents 2-fold increases in drug concentration during passaging; ceftazidime starting at 0.063 μM, norfloxacin starting at 0.016 μM. For each replicate of each treatment, drug-resistant bacteria that reached 50% growth under the highest concentration of ceftazidime were used to inoculate the following day's cultures. Data shows the mean of three independent passaging experiments for each treatment condition; error bars denote the standard error of the mean. The difference between day 1 and day 12 MIC values for each passaging condition was analyzed by a one-way ANOVA with Tukey's HSD test. Values with different letters are significantly different ($p < 0.05$), shared letters are not ($p > 0.05$). Source data are provided as a Source Data file.

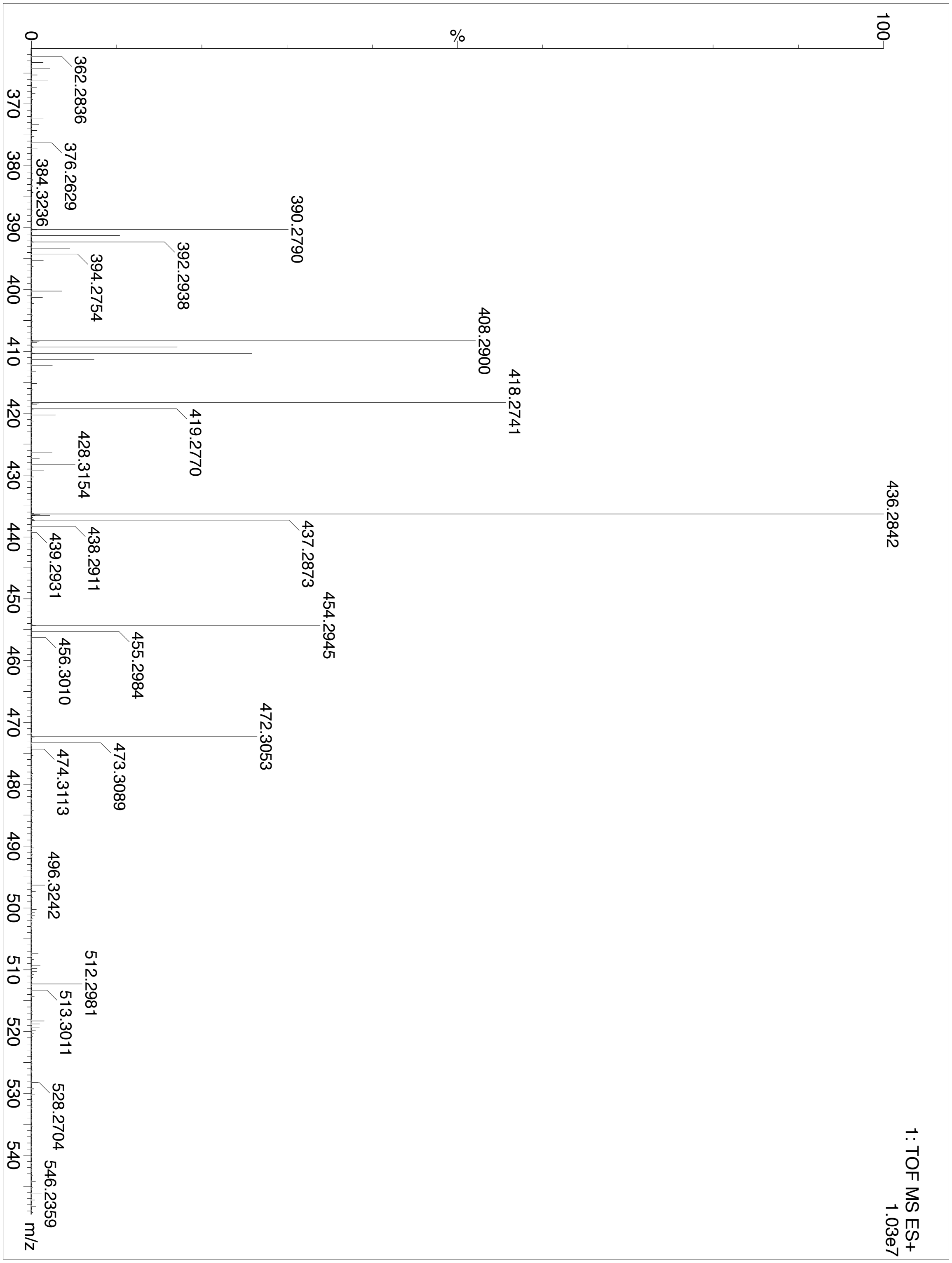

Supplementary Fig. 7 HRMS of borrelidin A (**1**).

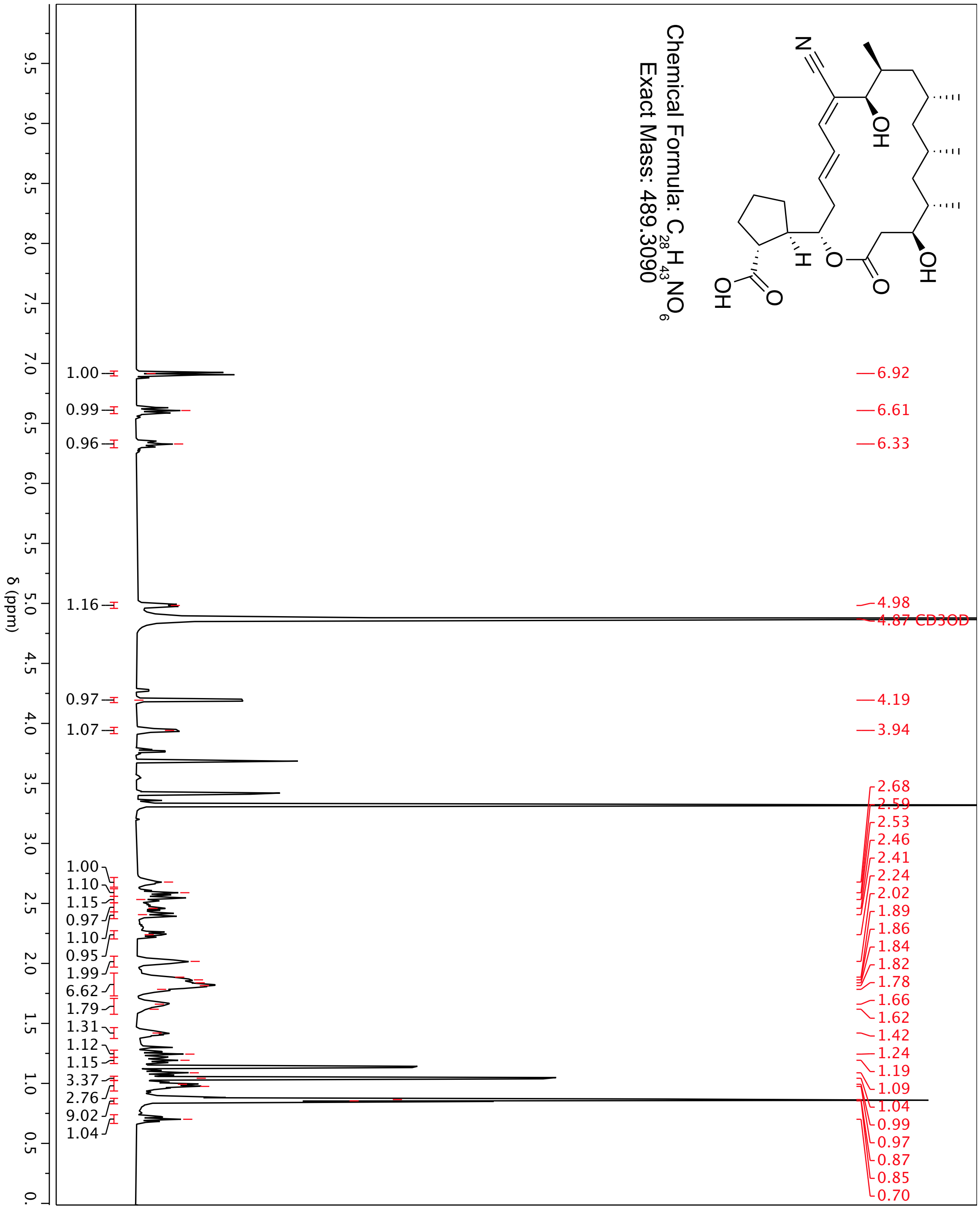

Supplementary Fig. 8 ^{1}H-NMR spectrum of borrelidin A (**1**) at 600 MHz in CD_3OD

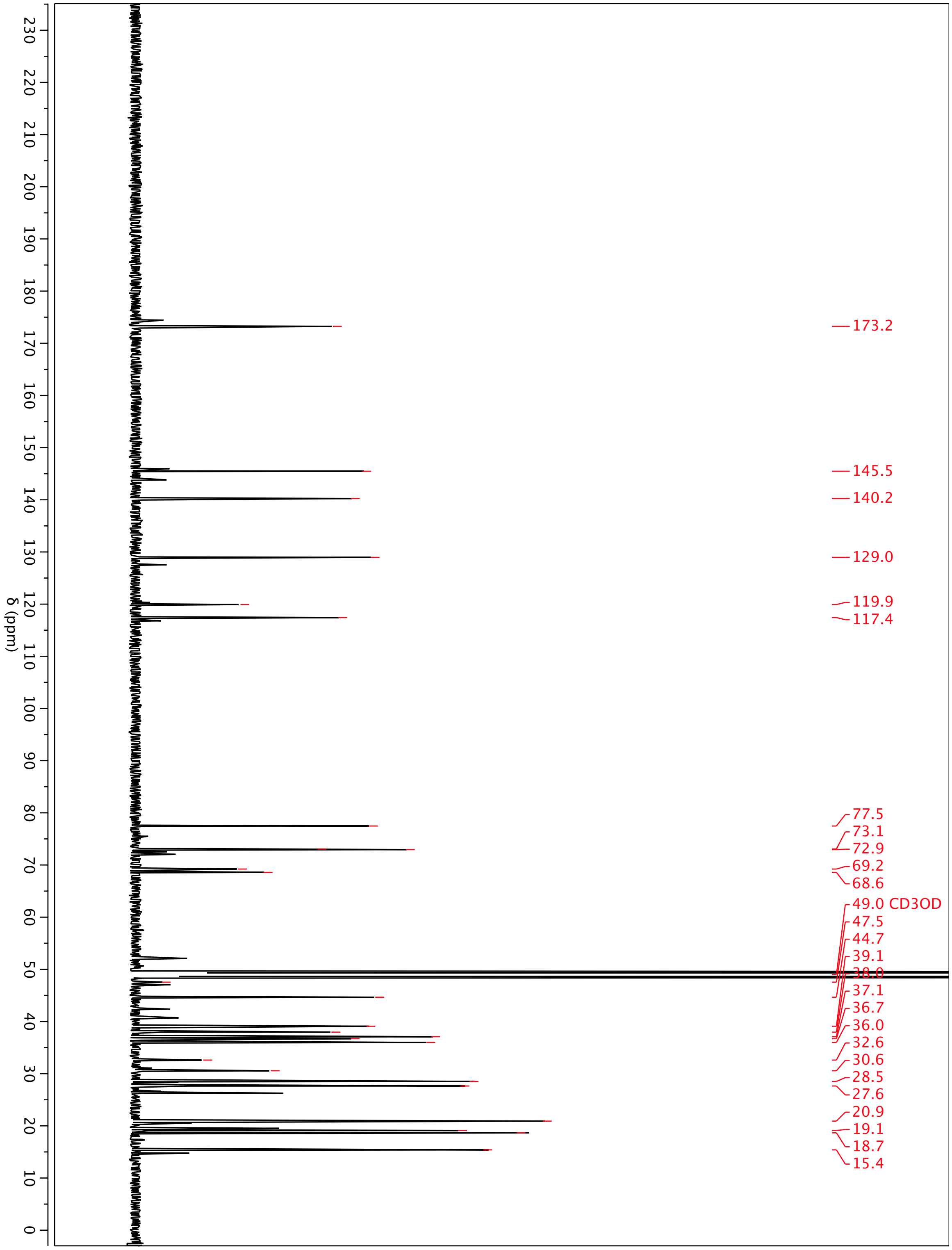

Supplementary Fig. 9 ^{13}C-NMR spectrum of borrelidin A (**1**) at 150 MHz in CD_3OD

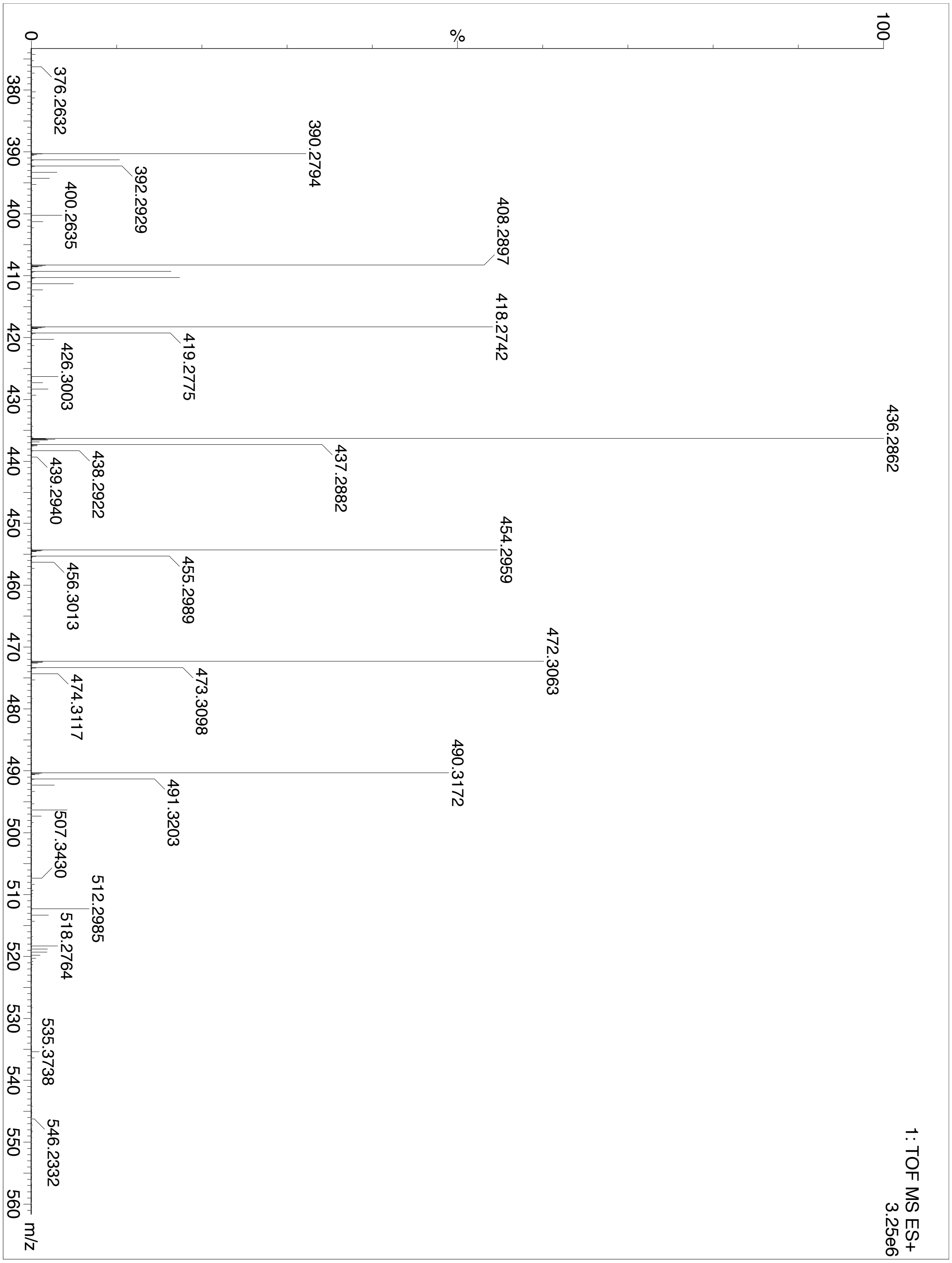

Supplementary Fig. 10 HRMS of borrelidin F (**2**).

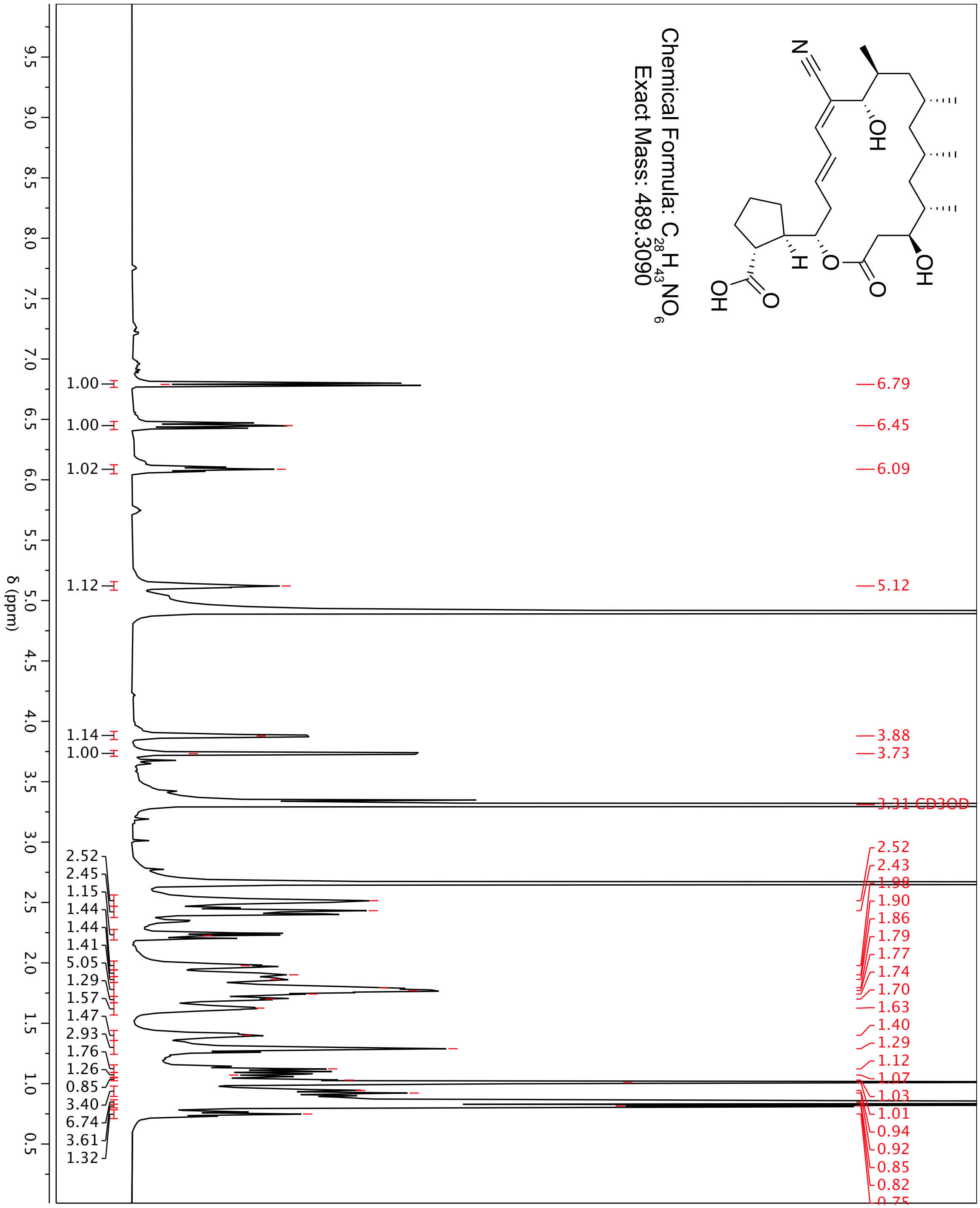

Supplementary Fig. 11 ^{1}H-NMR spectrum of borrelidin F (**2**) at 600 MHz in CD_3OD

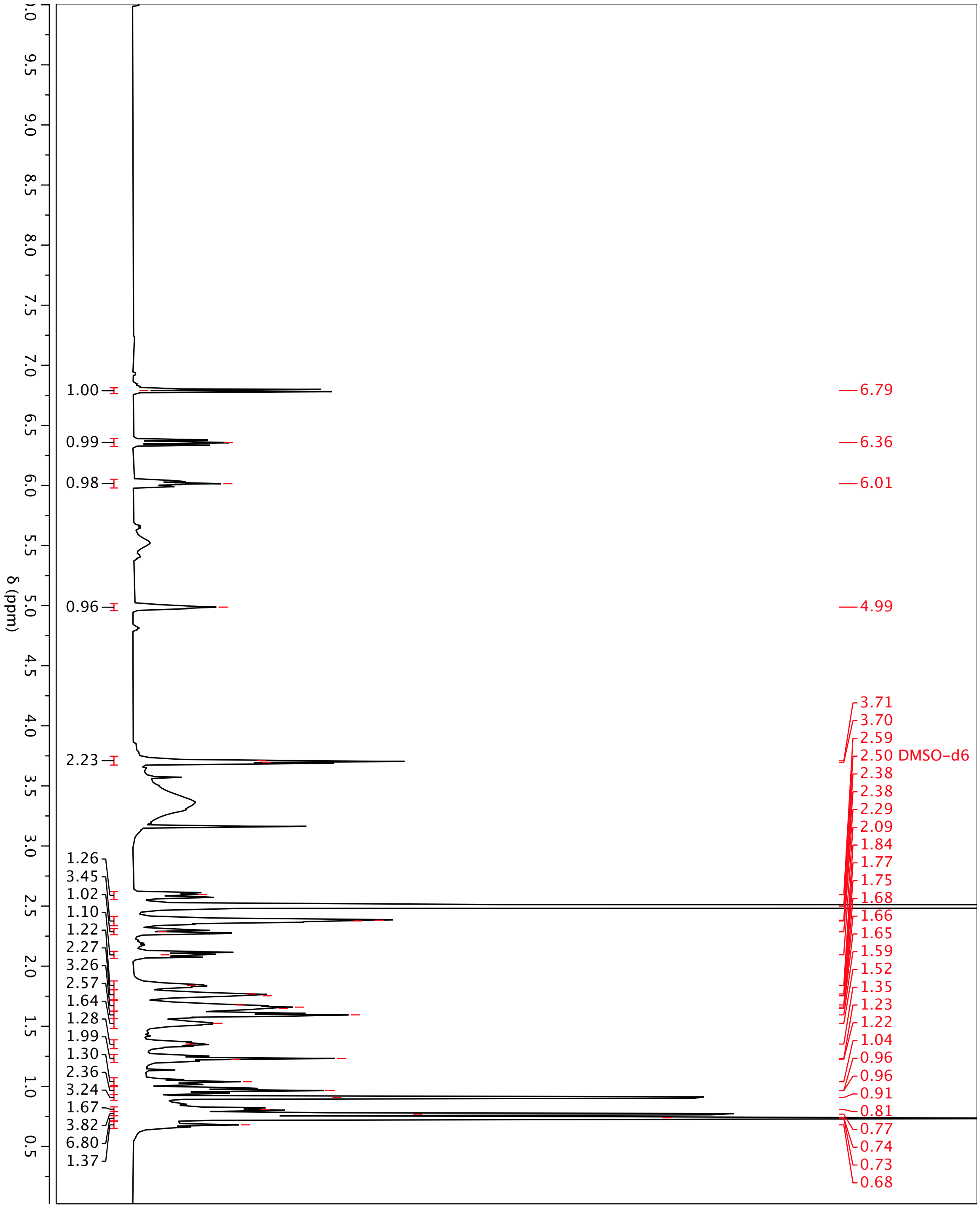

Supplementary Fig. 12 ^{1}H-NMR spectrum of borrelidin F (**2**) at 600 MHz in DMSO-d_6

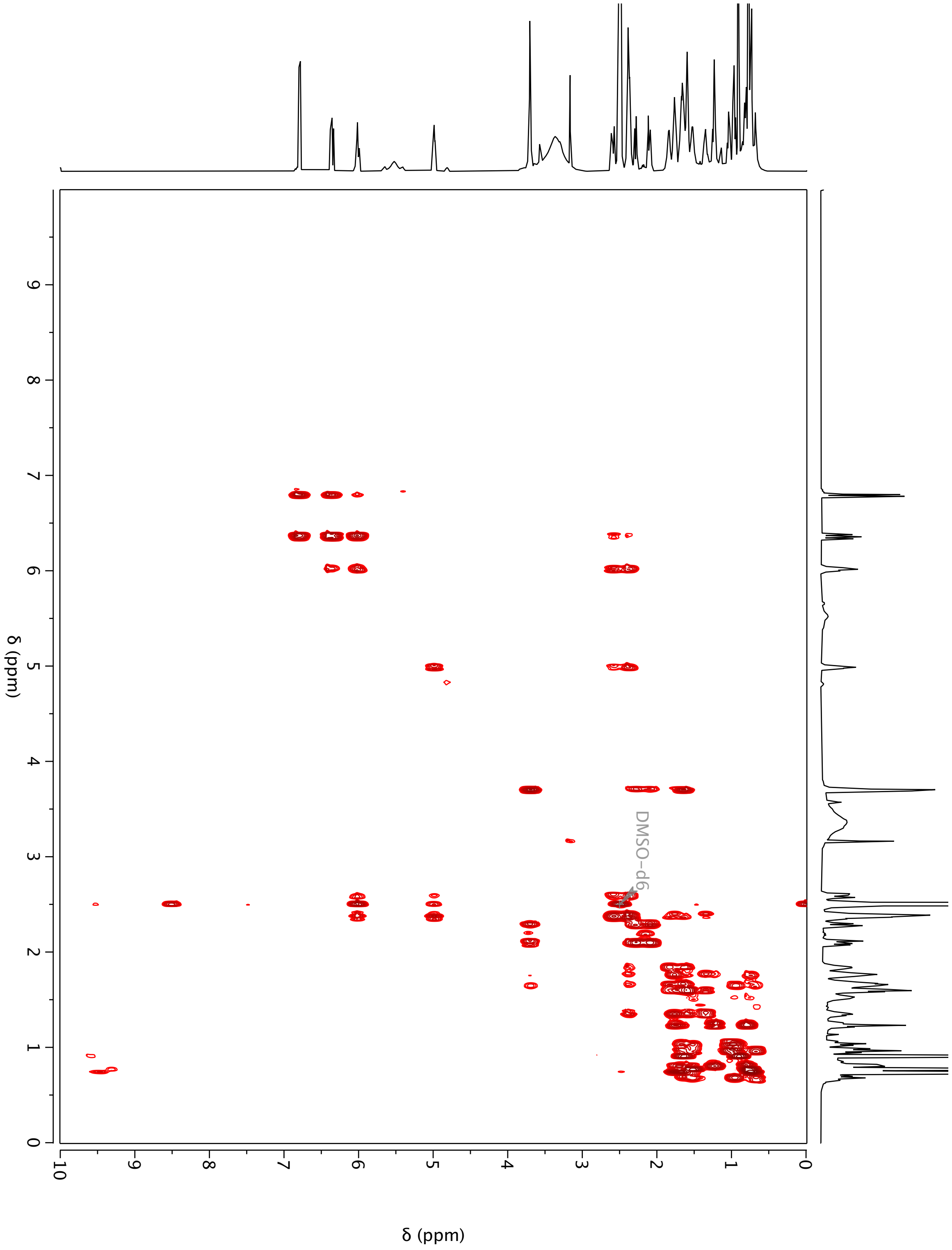

Supplementary Fig. 13 gCOSY spectrum of borrelidin F (**2**) at 600 MHz in DMSO-d_6

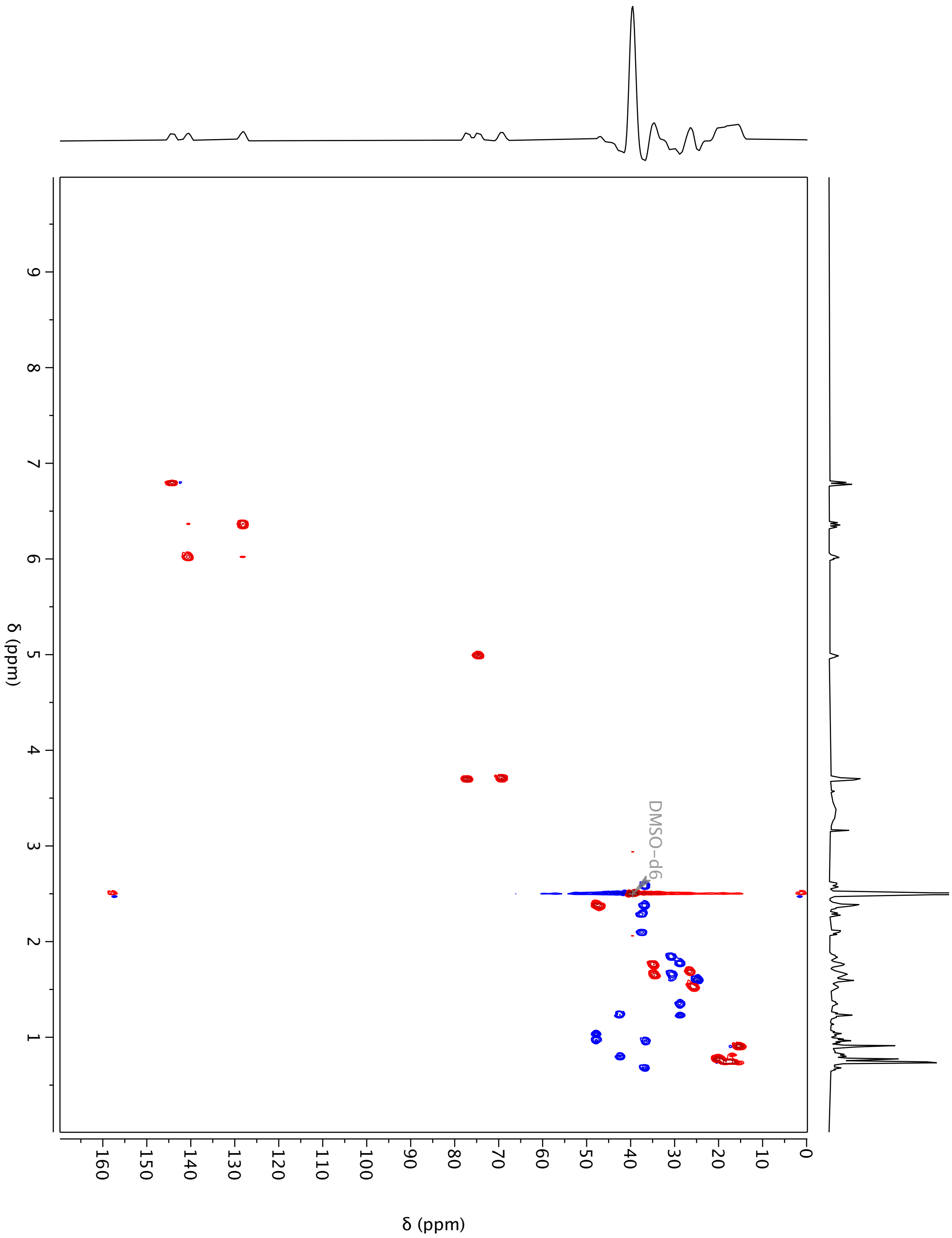

Supplementary Fig. 14 gHSQC spectrum of borrelidin F (**2**) at 600 MHz in DMSO-d_6

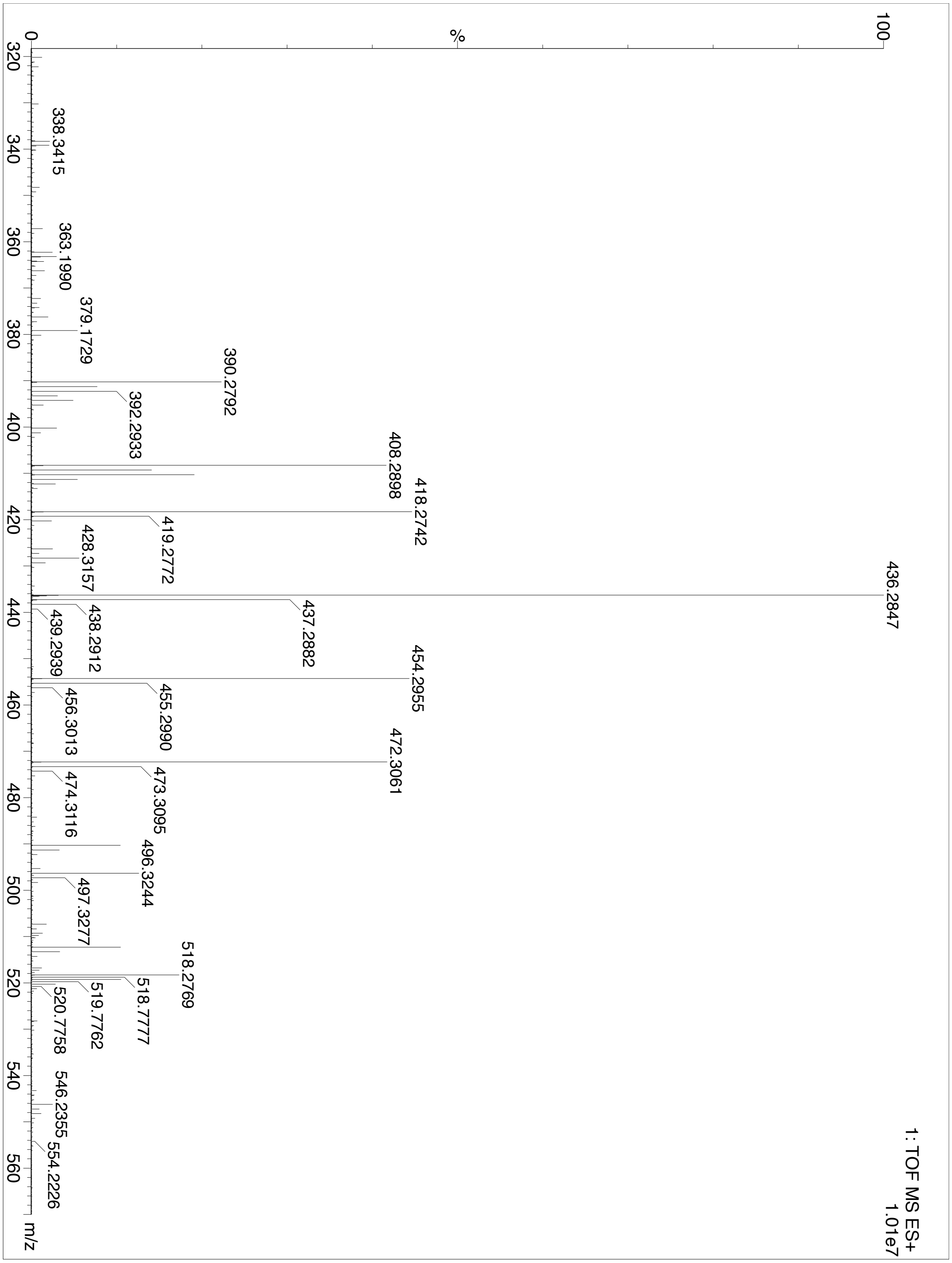

Supplementary Fig. 15 HRMS of borrelidin H (**3**).

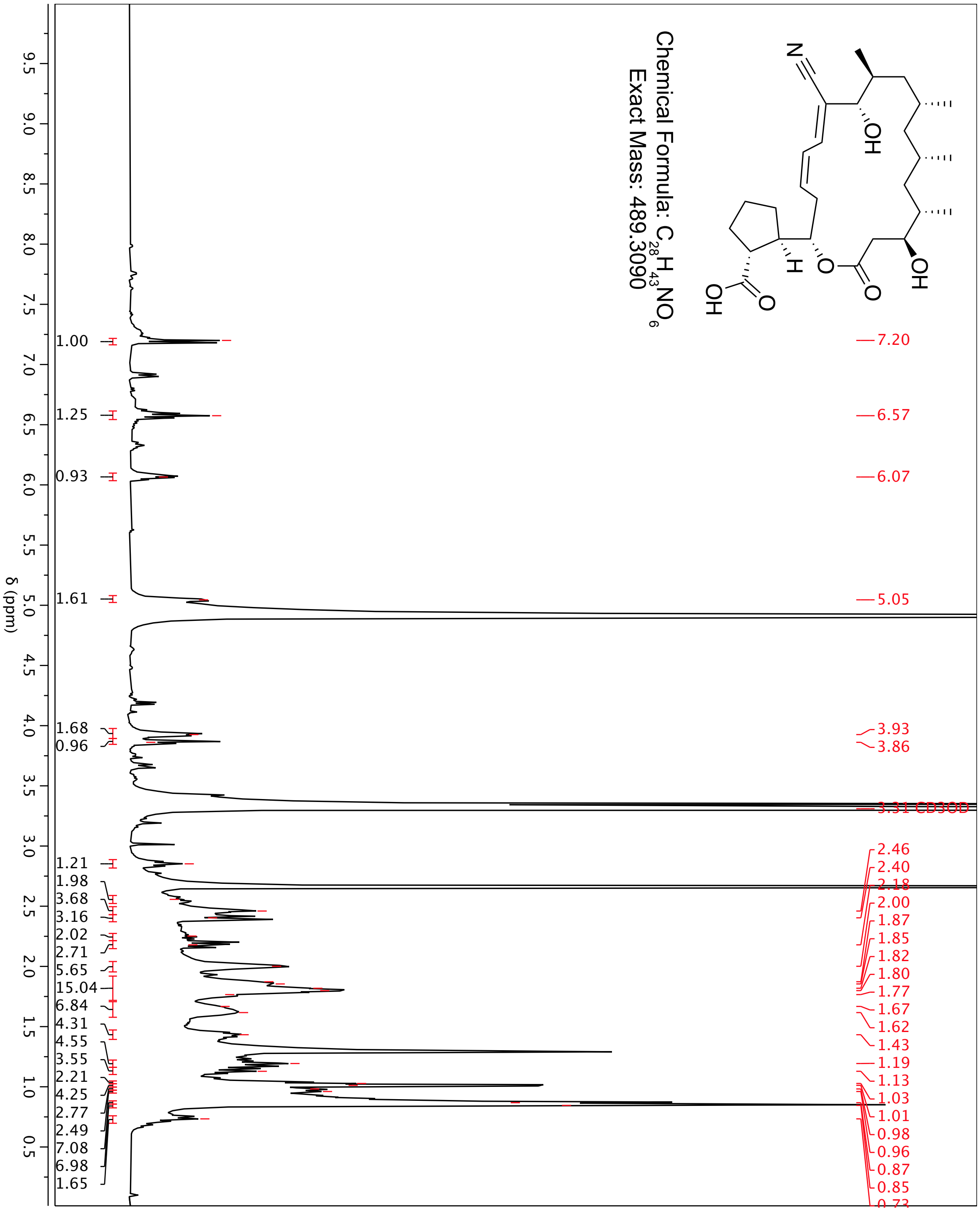

Supplementary Fig. 16 ^{1}H-NMR spectrum of borrelidin H (**3**) at 600 MHz in CD_3OD

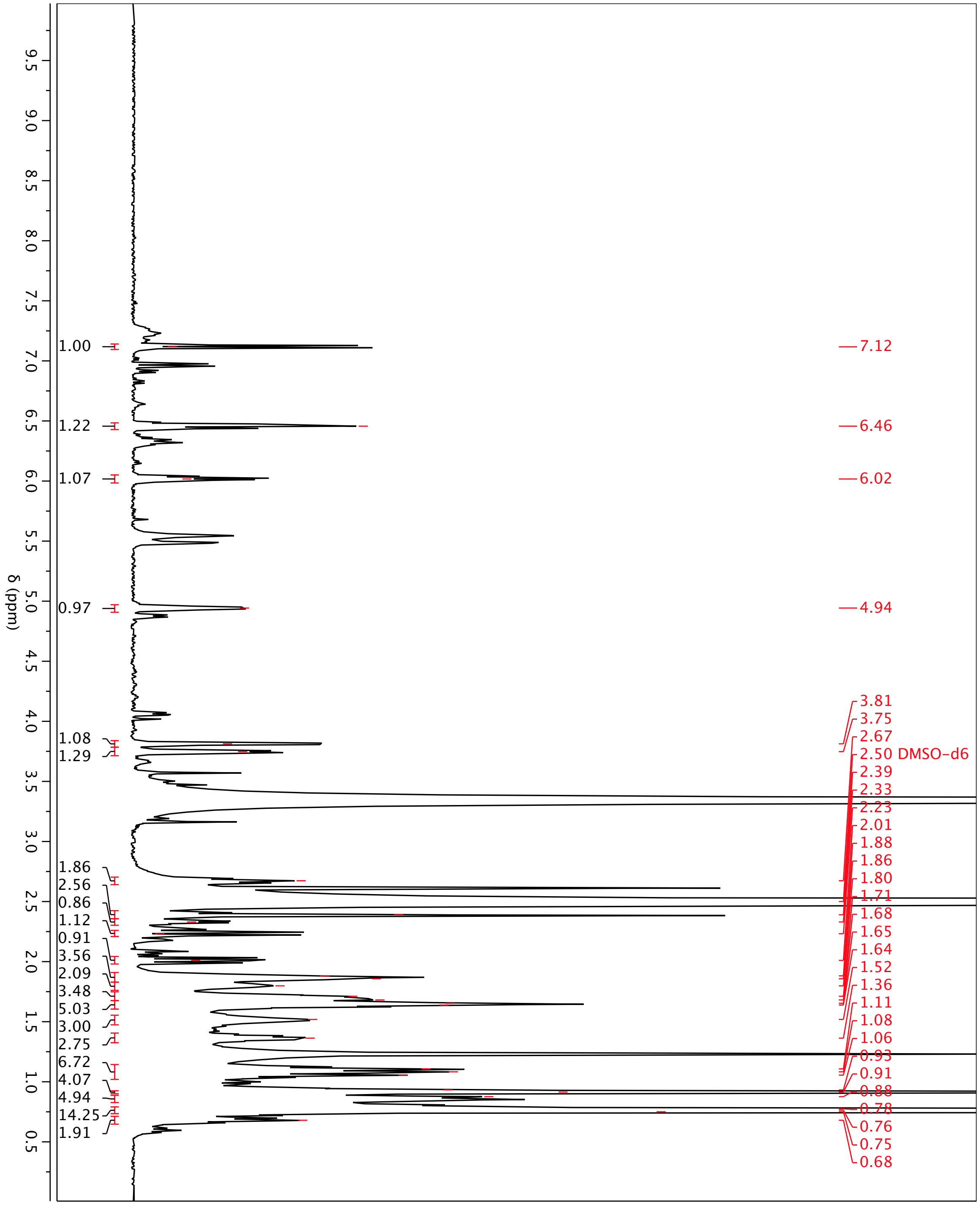

Supplementary Fig. 17 ^{1}H-NMR spectrum of borrelidin H (**3**) at 600 MHz in DMSO-d_6

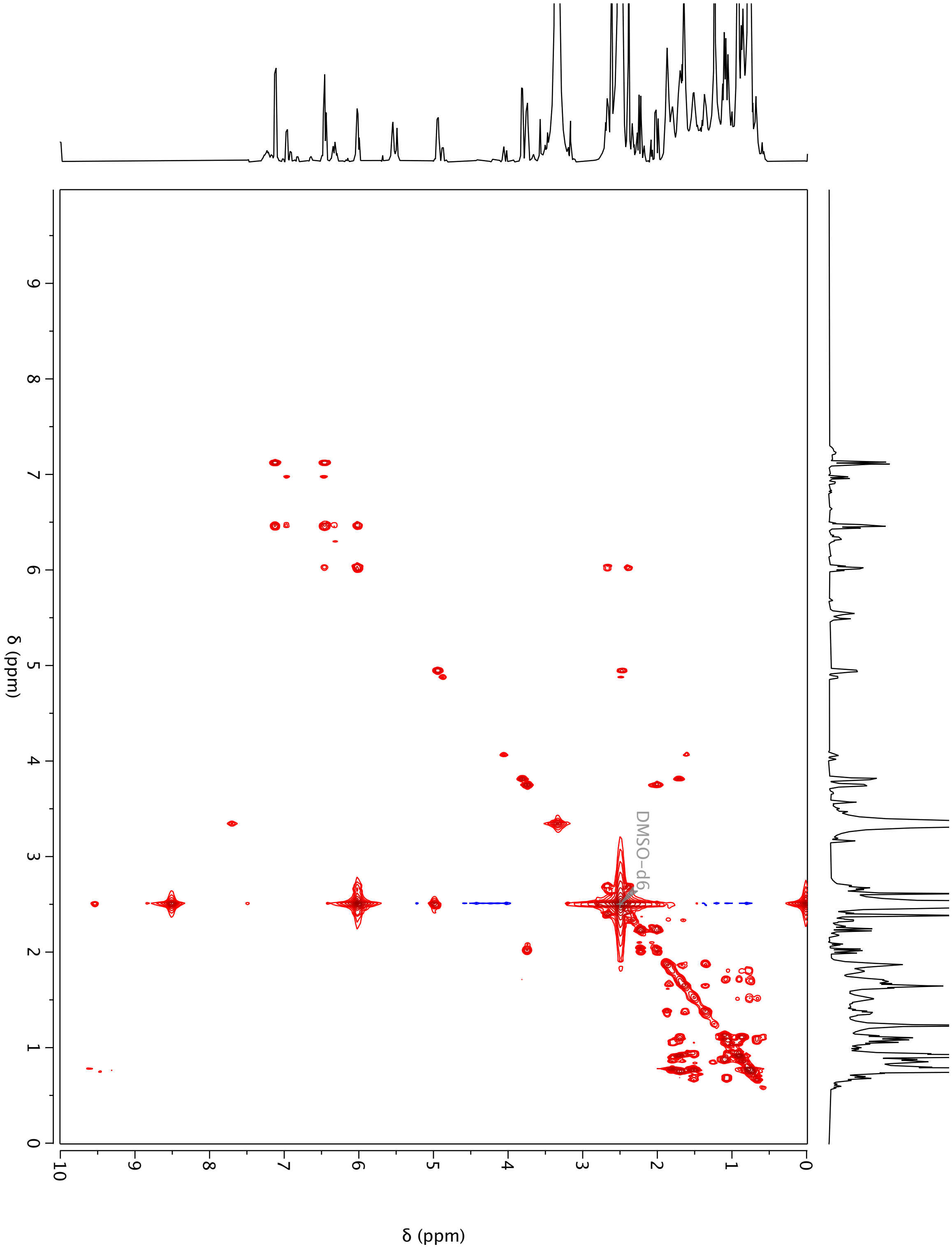

Supplementary Fig. 18 gCOSY spectrum of borrelidin H (**3**) at 600 MHz in DMSO-d$_6$

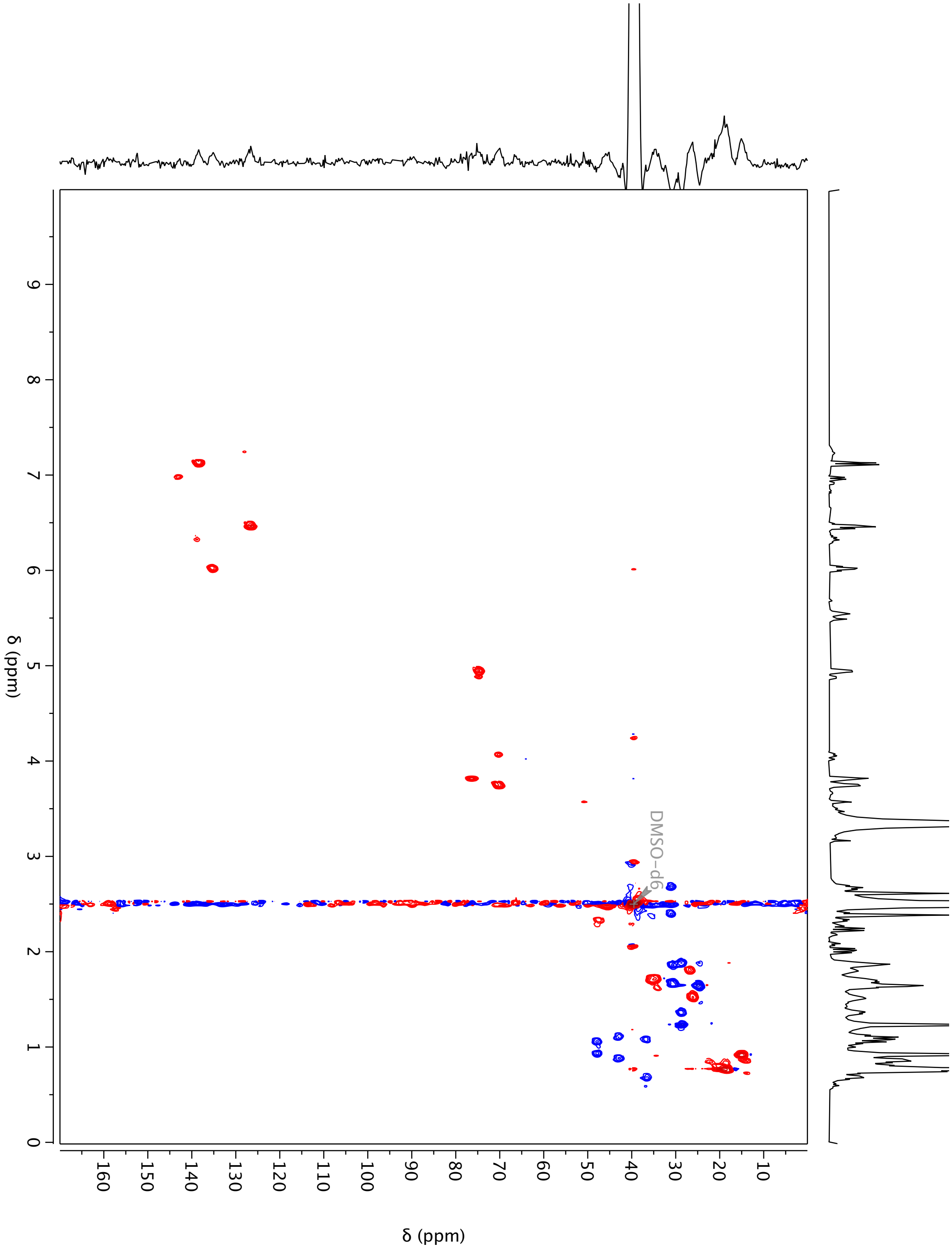

Supplementary Fig. 19 gHSQC spectrum of borrelidin H (**3**) at 600 MHz in DMSO-d$_6$

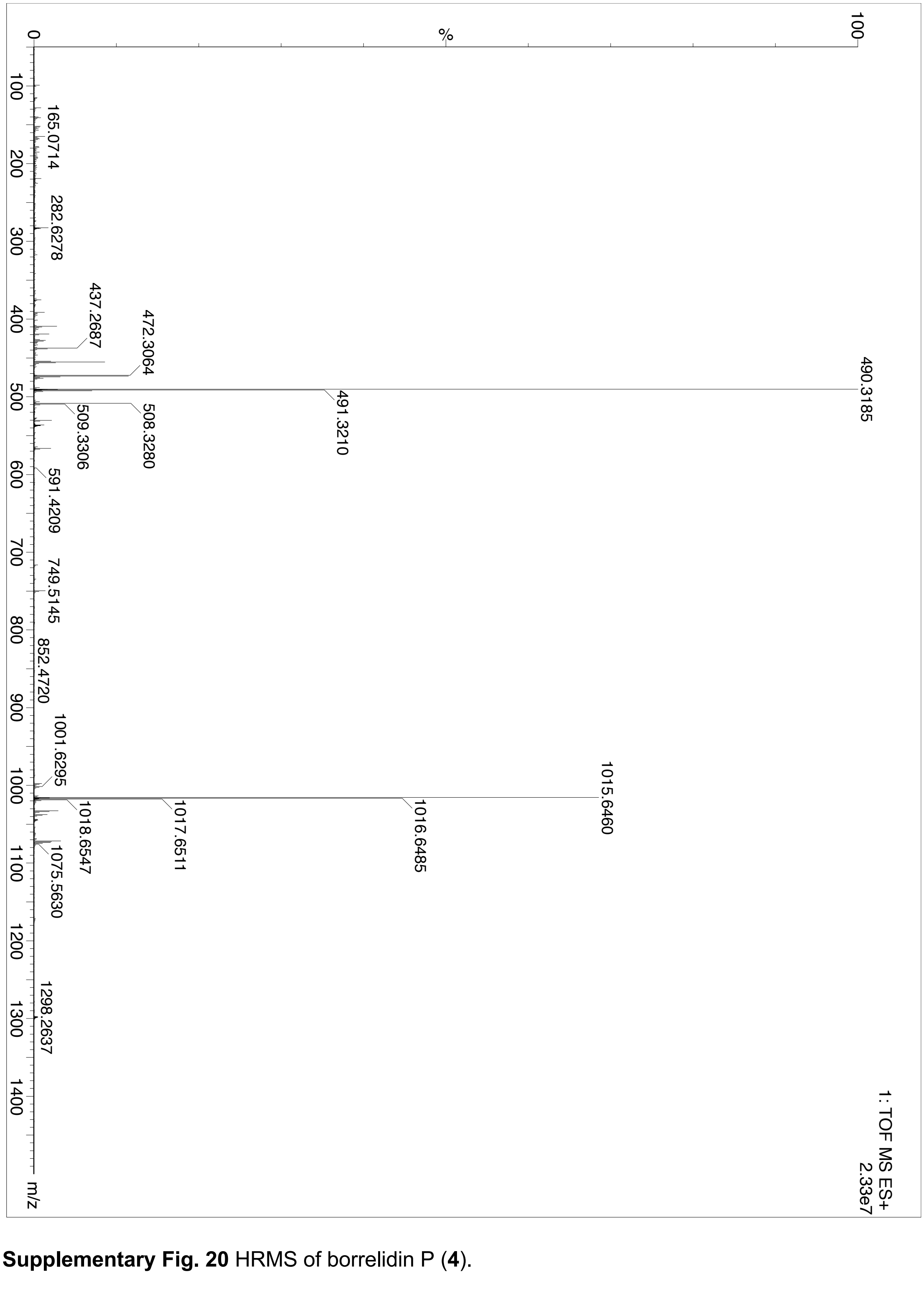

Supplementary Fig. 20 HRMS of borrelidin P (**4**).

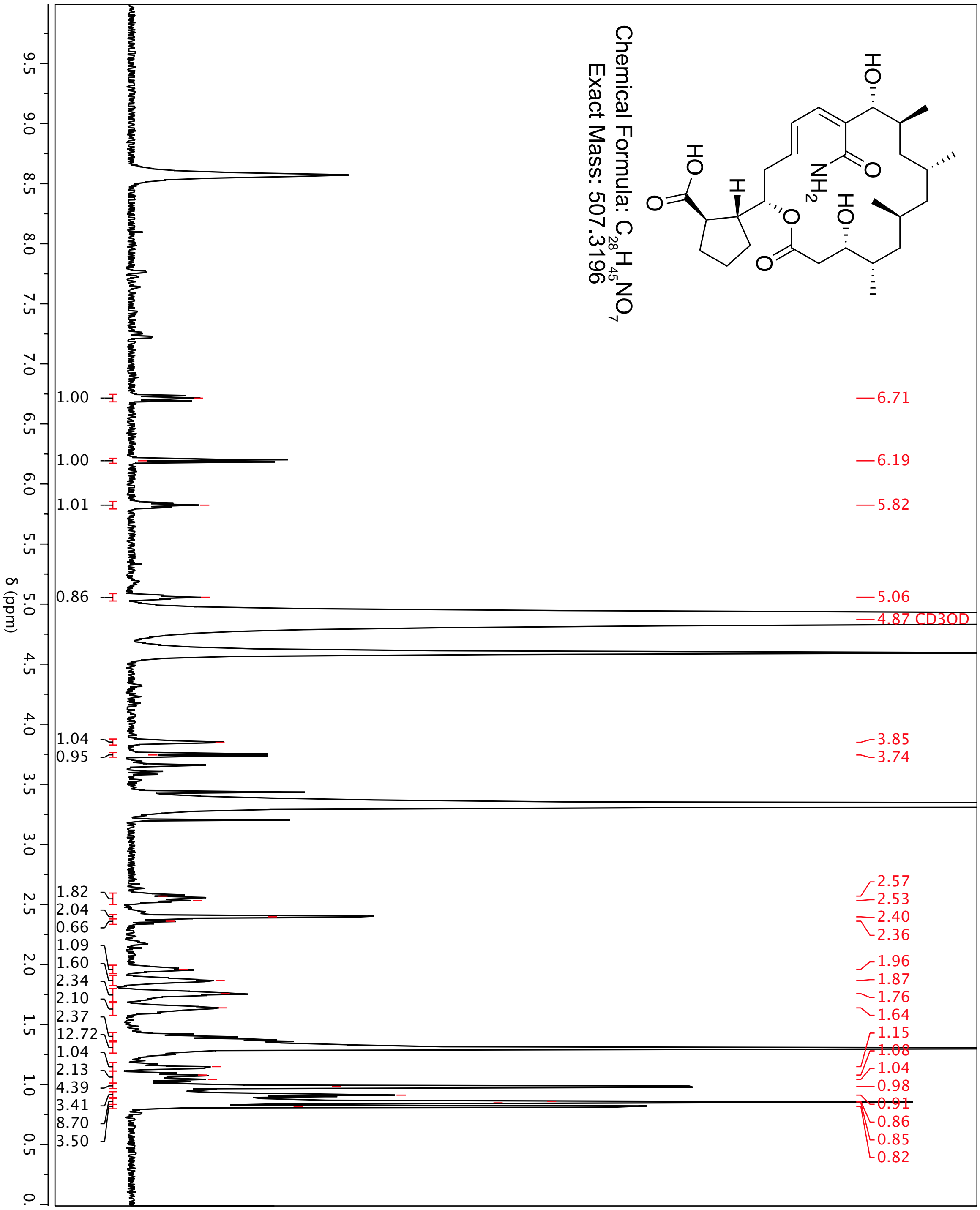

Supplementary Fig. 21 ^{1}H-NMR spectrum of borrelidin P (**4**) at 600 MHz in CD_3OD

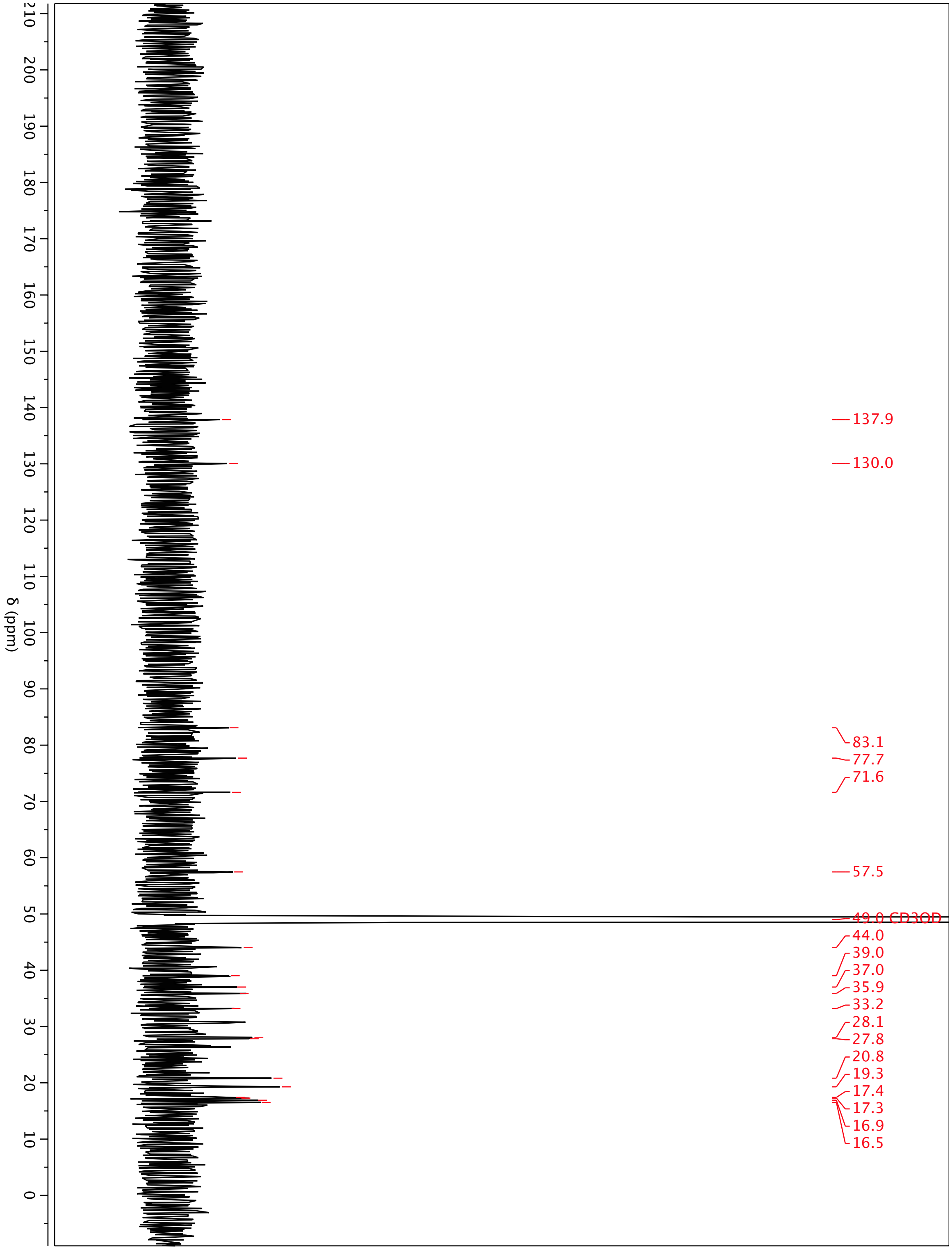

Supplementary Fig. 22 ^{13}C-NMR spectrum of borrelidin P (**4**) at 150 MHz in CD_3OD

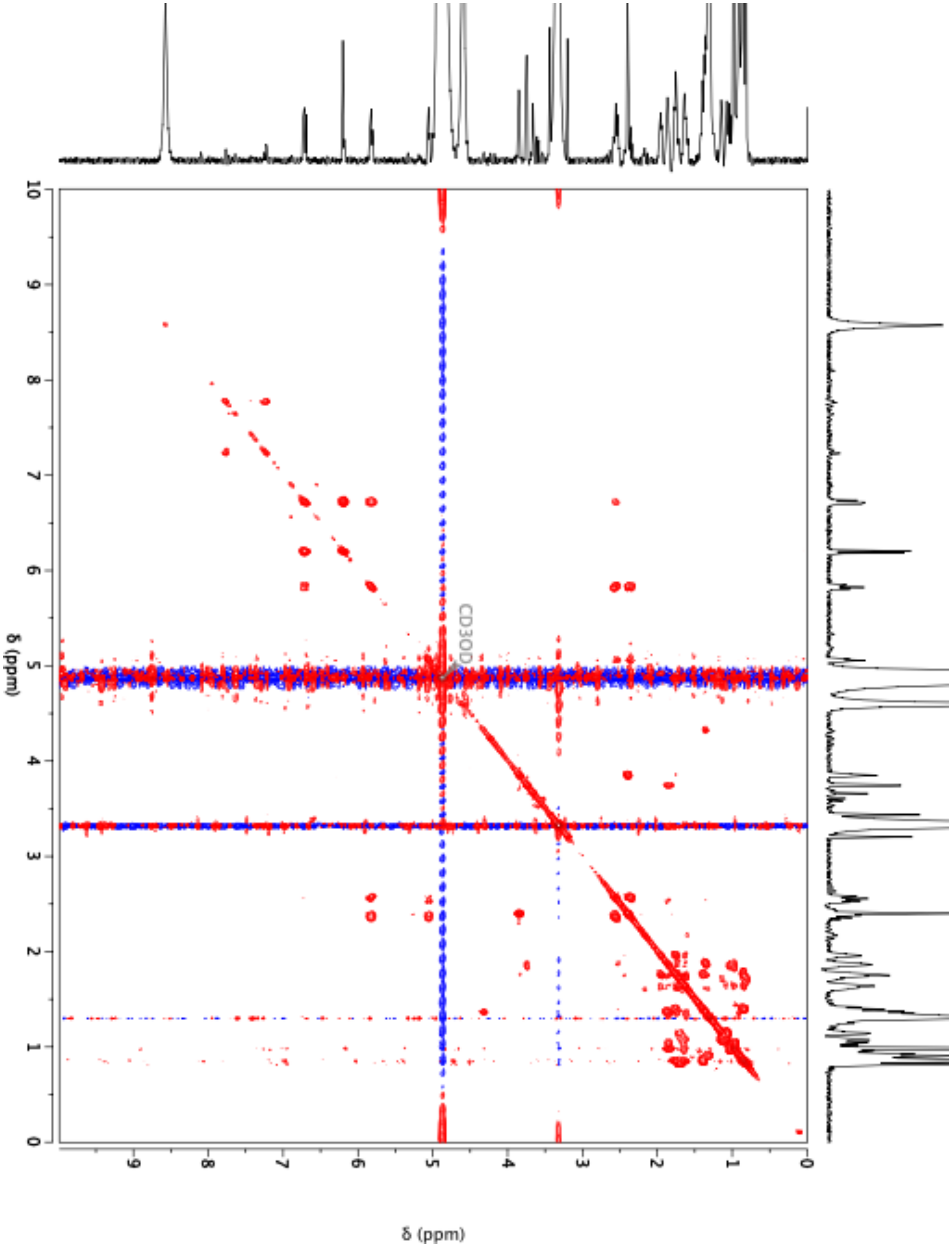

Supplementary Fig. 23 gCOSY spectrum of borrelidin P (**4**) at 600 MHz in CD3OD

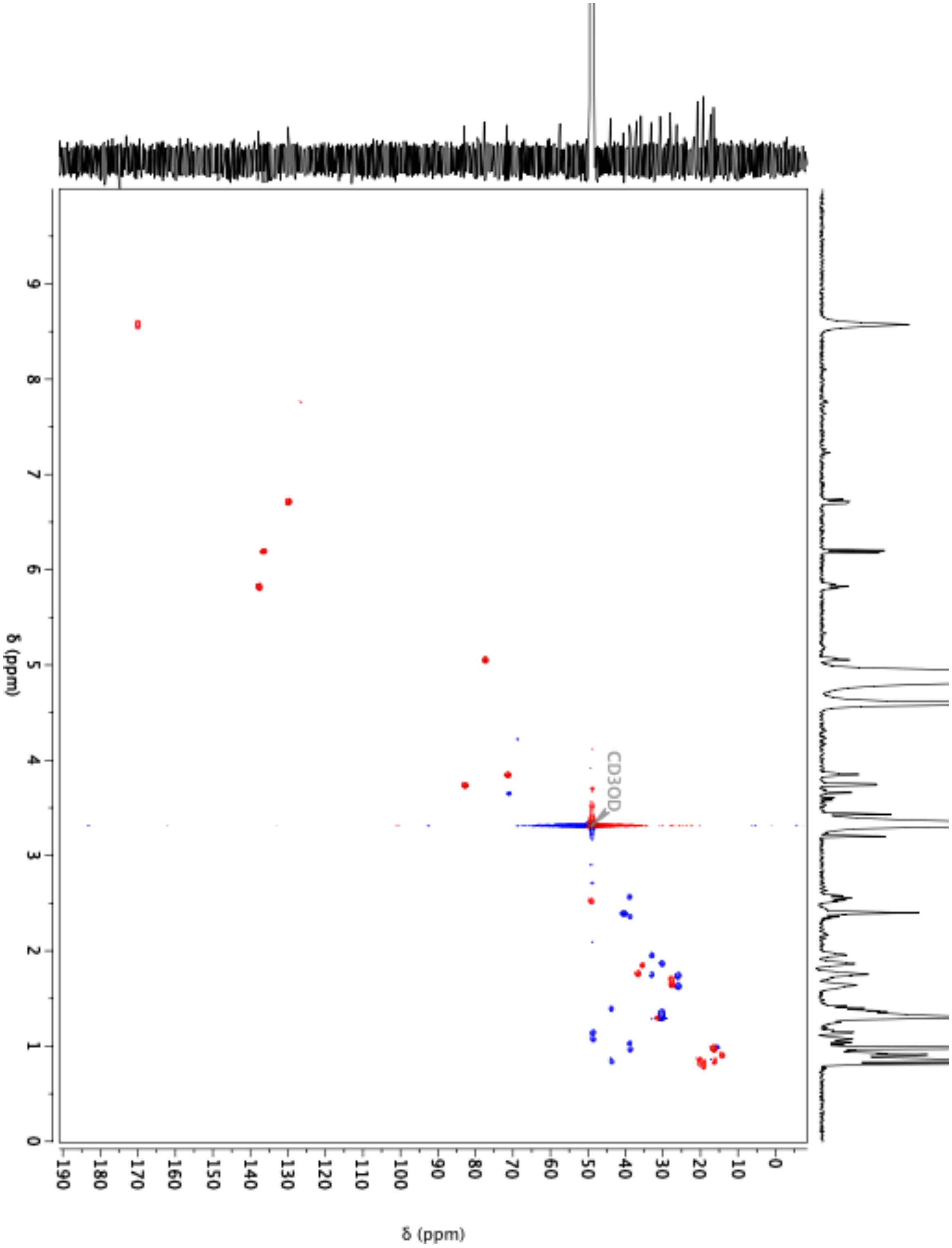

Supplementary Fig. 24 gHSQC spectrum of borrelidin P (**4**) at 600 MHz in CD_3OD

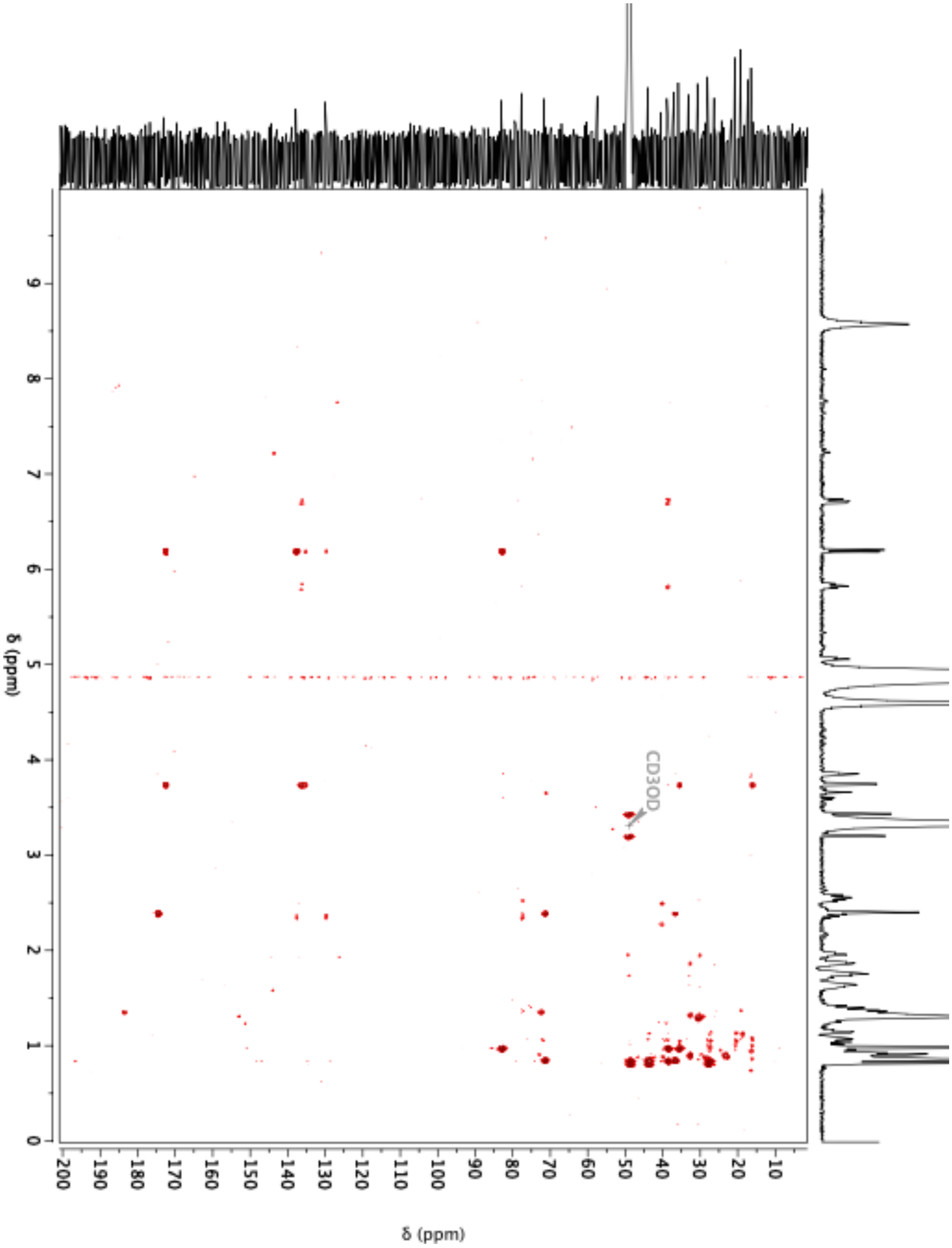

Supplementary Fig. 25 gHMBC spectrum of borrelidin P (**4**) at 600 MHz in CD_3OD

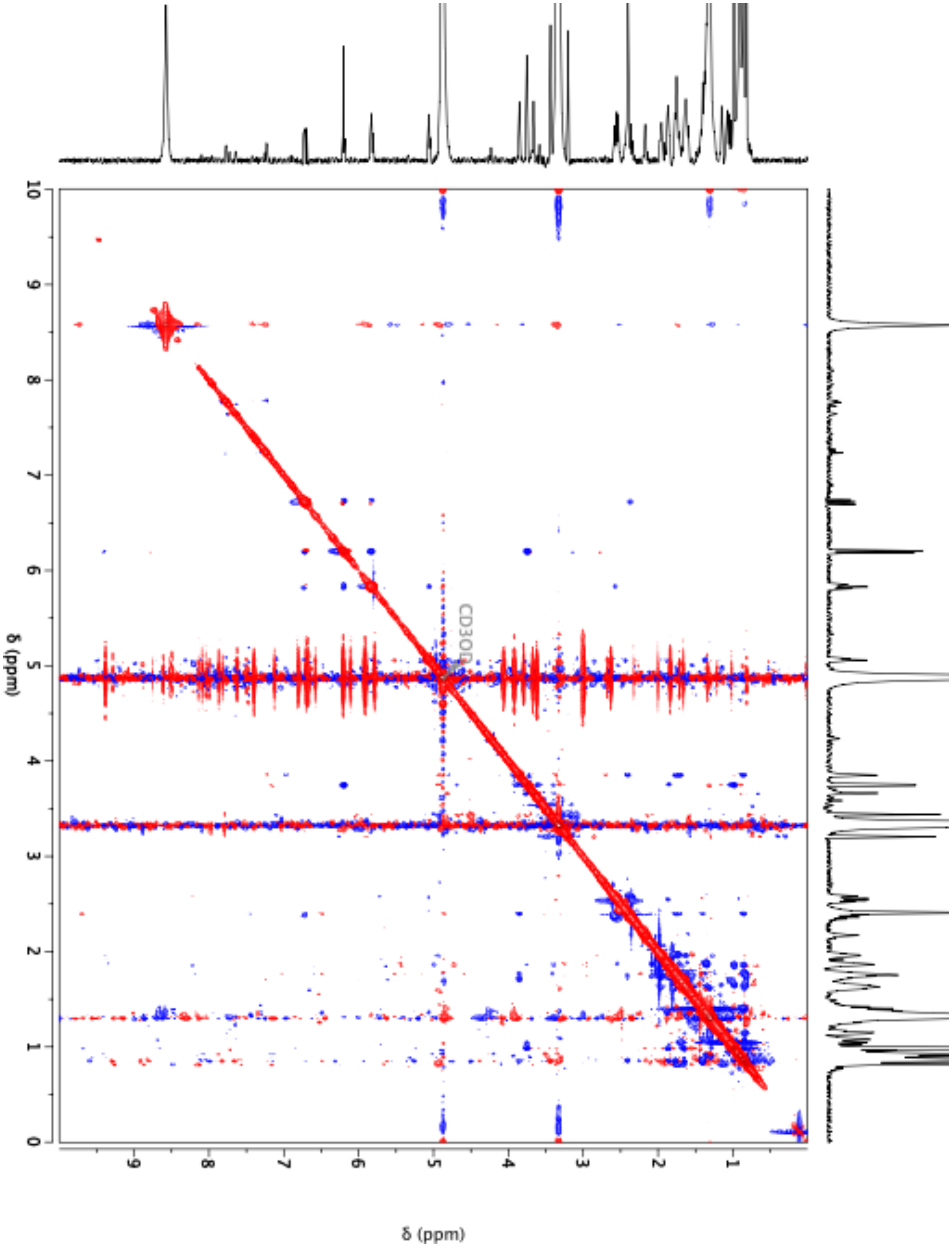

Supplementary Fig. 26 NOESY spectrum of borrelidin P (**4**) at 600 MHz in CD_3OD

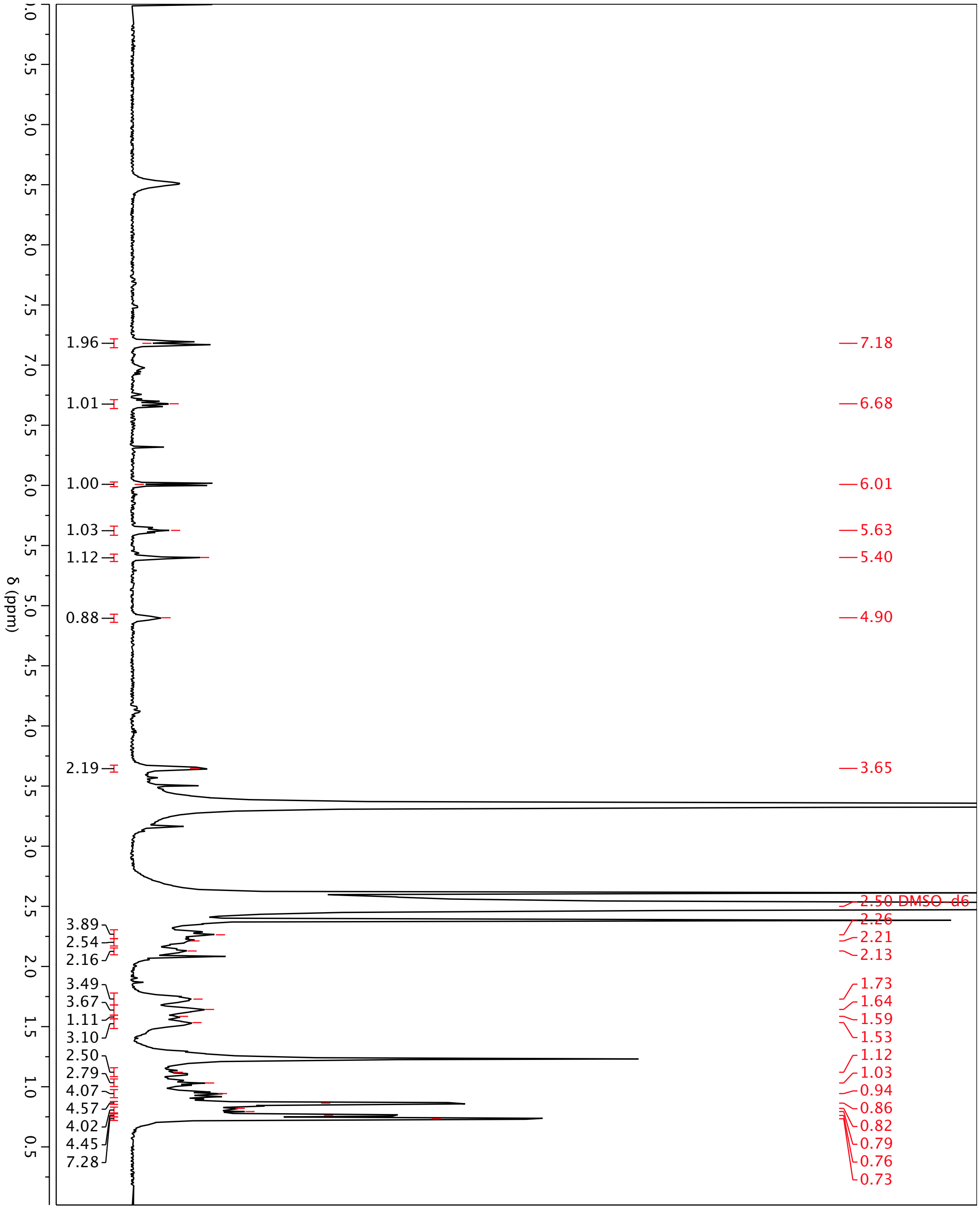

Supplementary Fig. 27 ^{1}H-NMR spectrum of borrelidin P (**4**) at 600 MHz in DMSO-d_6

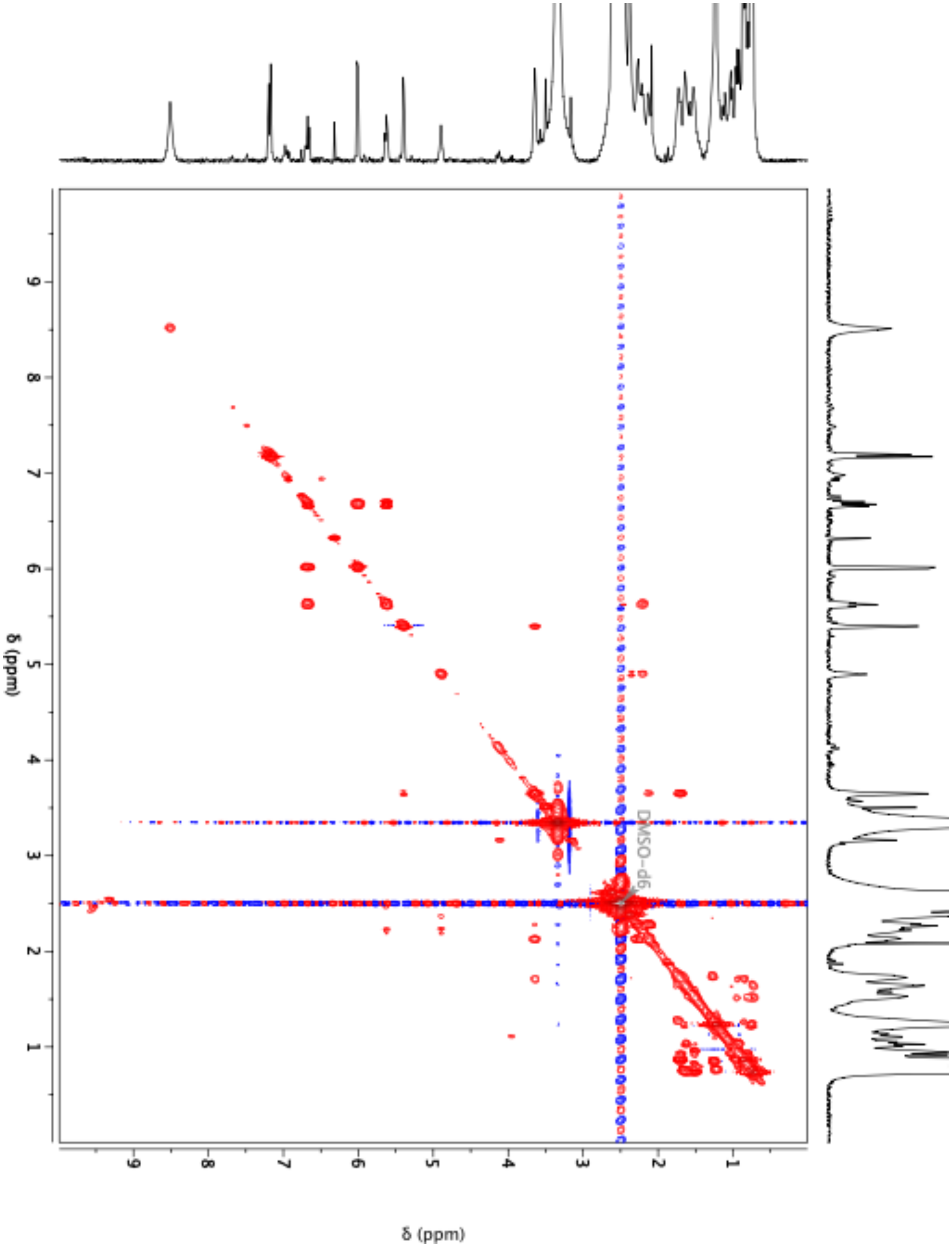

Supplementary Fig. 28 gCOSY spectrum of borrelidin P (**4**) at 600 MHz in DMSO-d_6

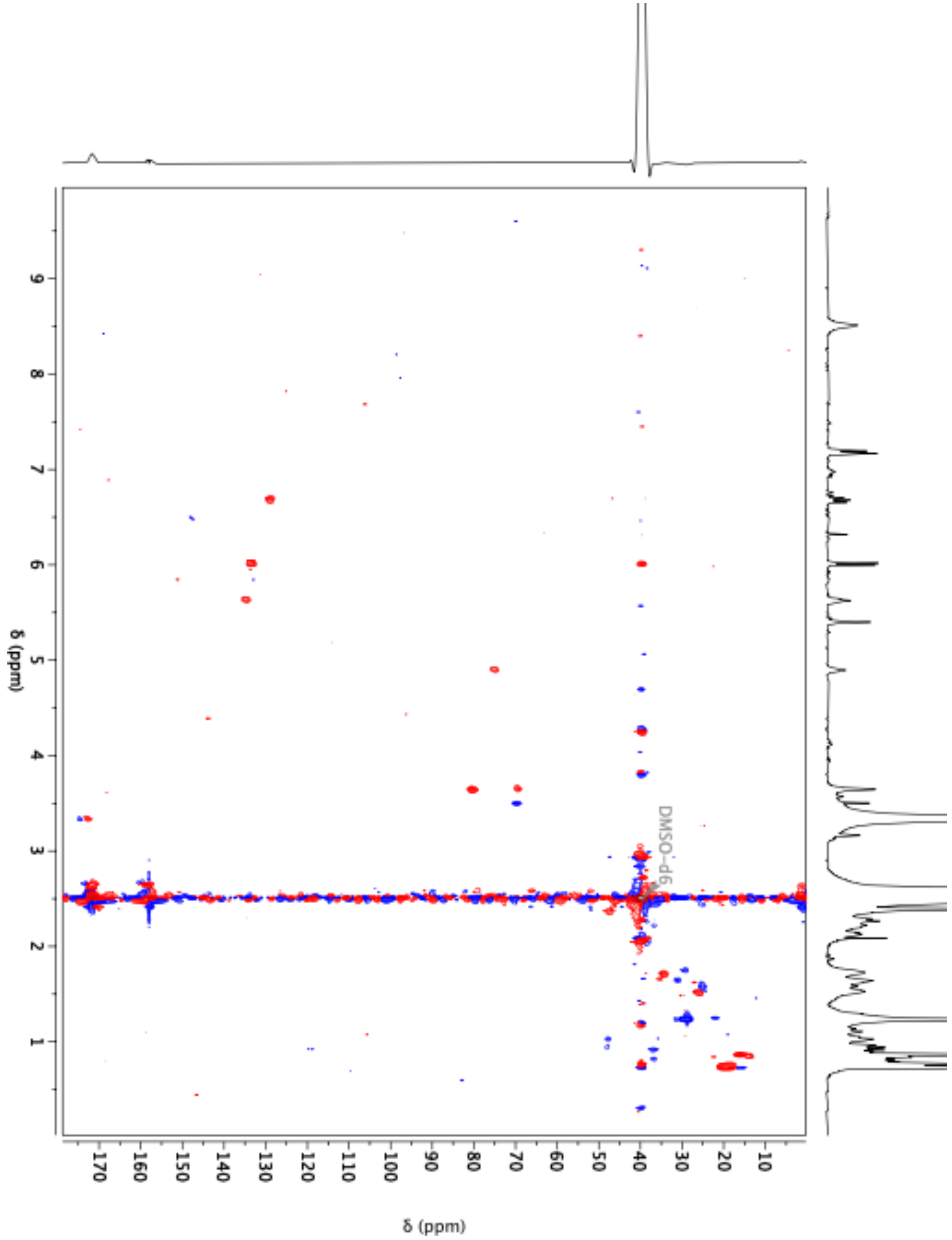

Supplementary Fig. 29 gHSQC spectrum of borrelidin P (**4**) at 600 MHz in DMSO-d_6

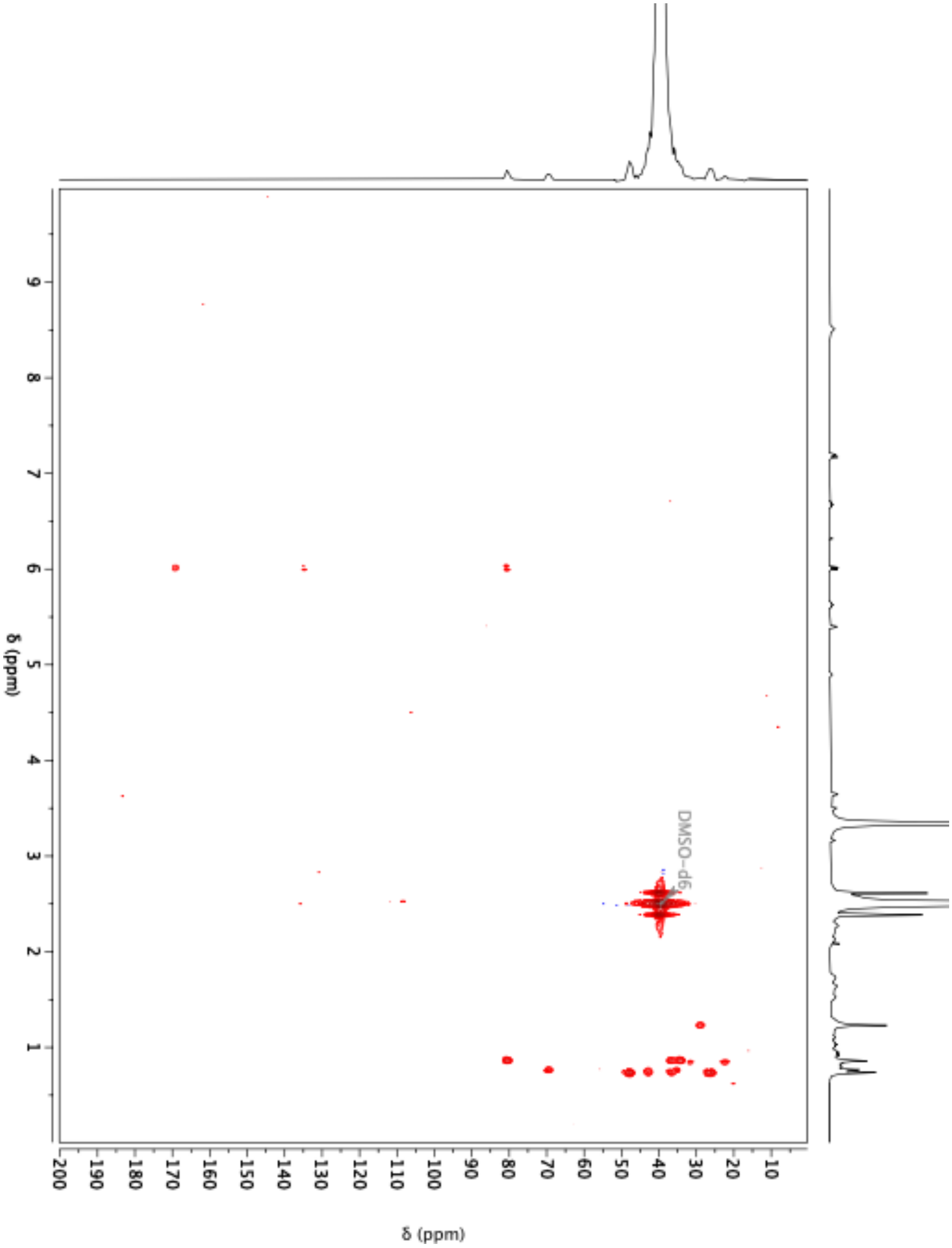

Supplementary Fig. 30 gHMBC spectrum of borrelidin P (**4**) at 600 MHz in DMSO-d_6

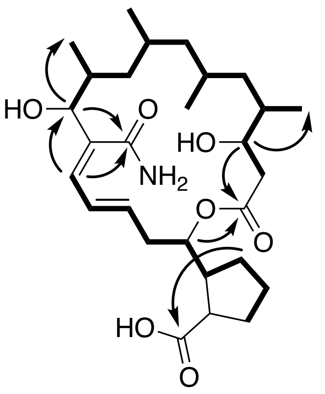

(**4**)

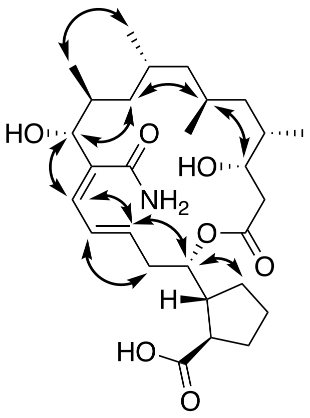

(**4**)

Supplementary Fig. 31 Key 2D-NMR correlations of compound **4** in methanol-d4 at 600 MHz and 150 MHz for ^{1}H and ^{13}C, respectively. COSY (bold), select HMBC (one-headed arrows), and select NOESY (two-headed arrows) correlations.

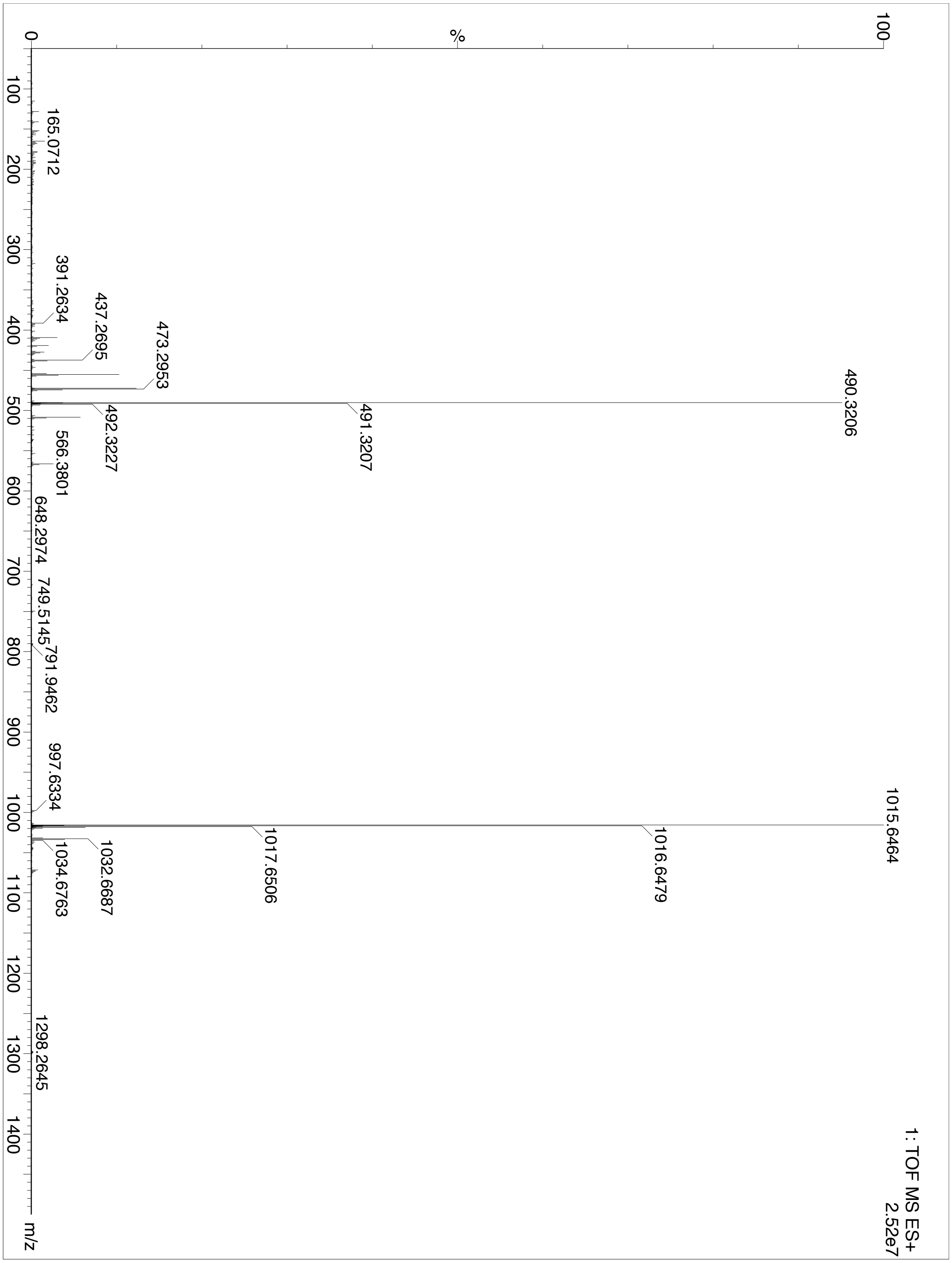

Supplementary Fig. 32 HRMS of 12-desnitrile-12-carbamoyl-borrelidin A (**5**)

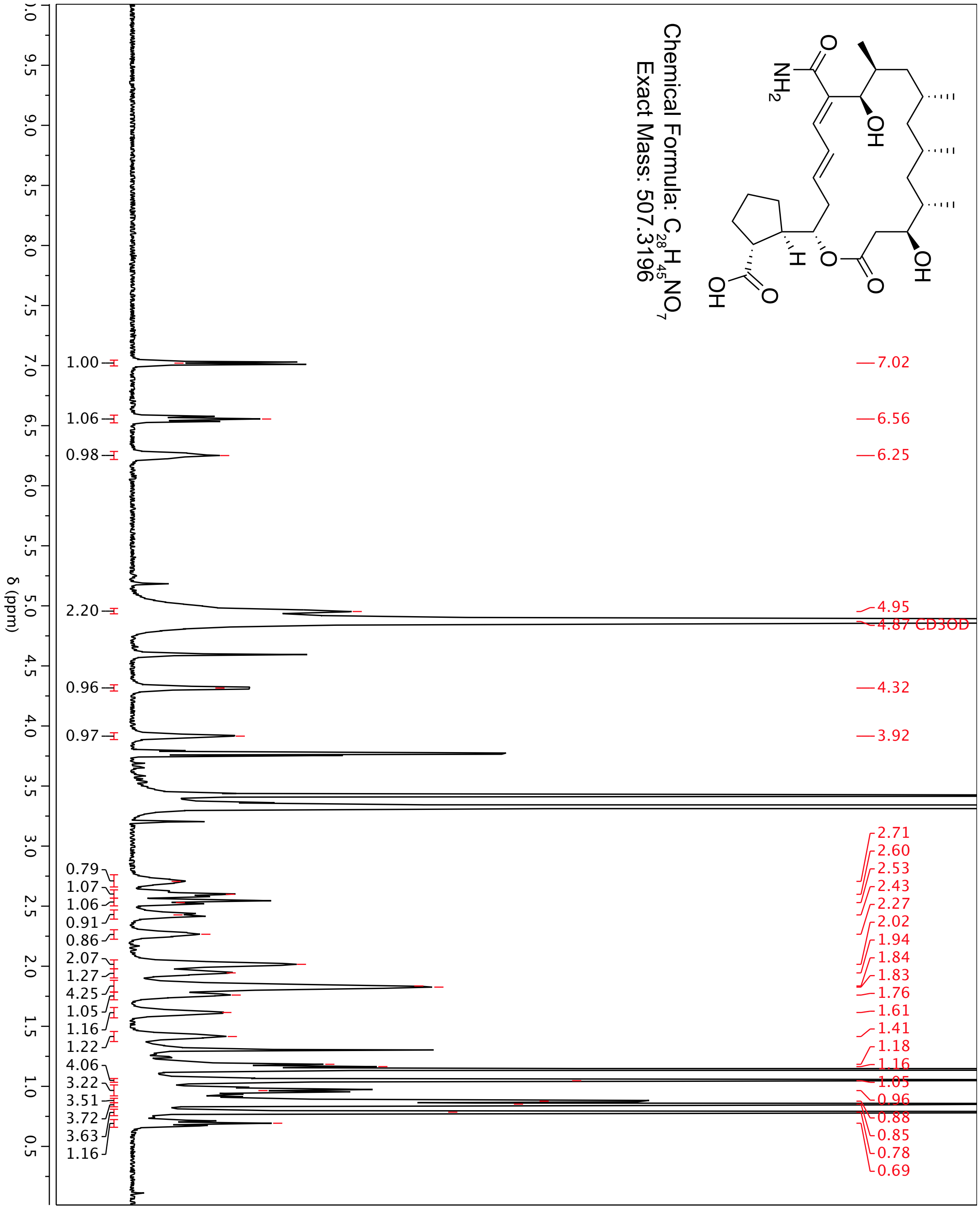

Supplementary Fig. 33 ^{1}H-NMR spectrum of 12-desnitrile-12-carbamoyl-borrelidin A (**5**) at 600 MHz in CD_3OD

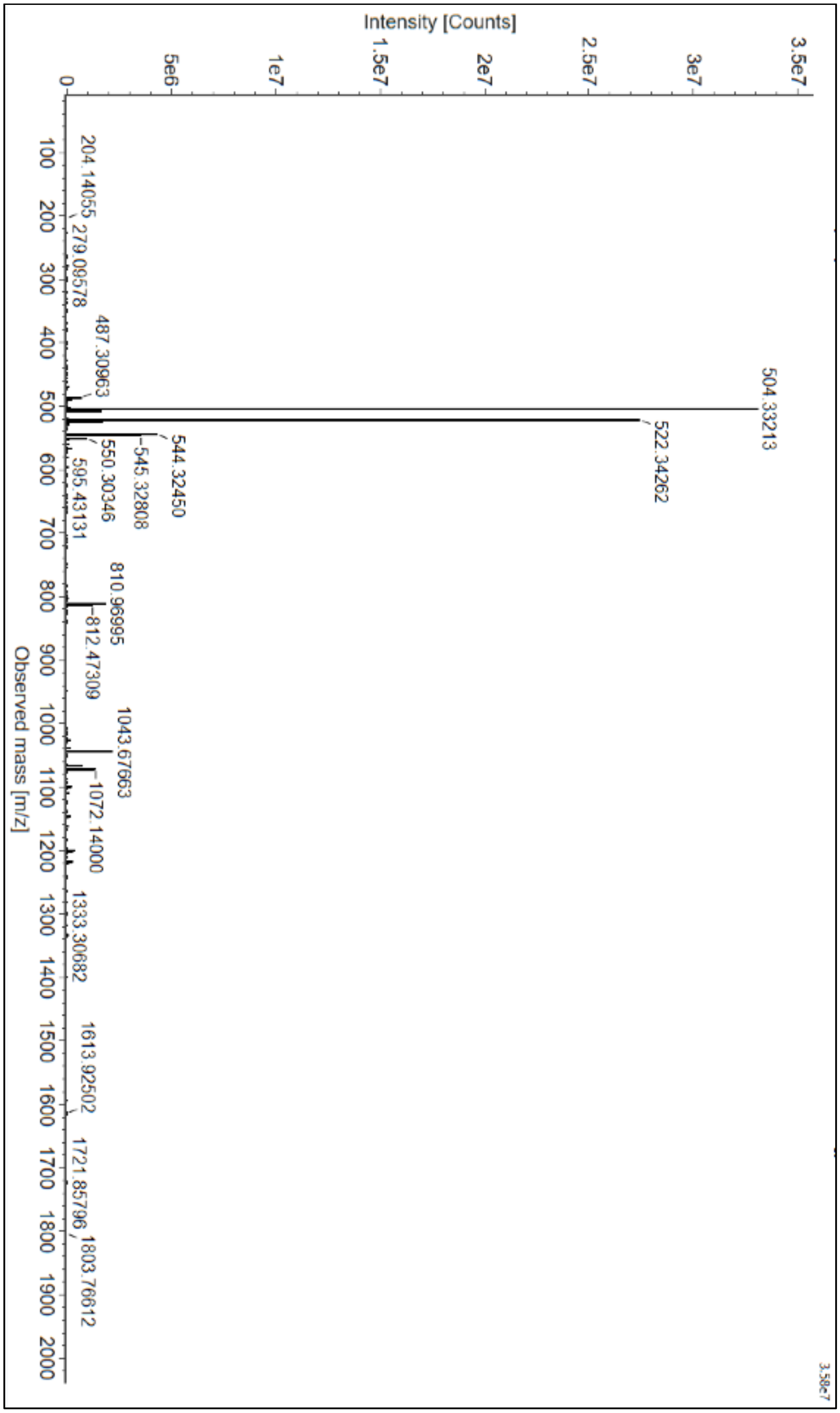

Supplementary Fig. 34 HRMS of borrelidin P methyl ester (**6**)

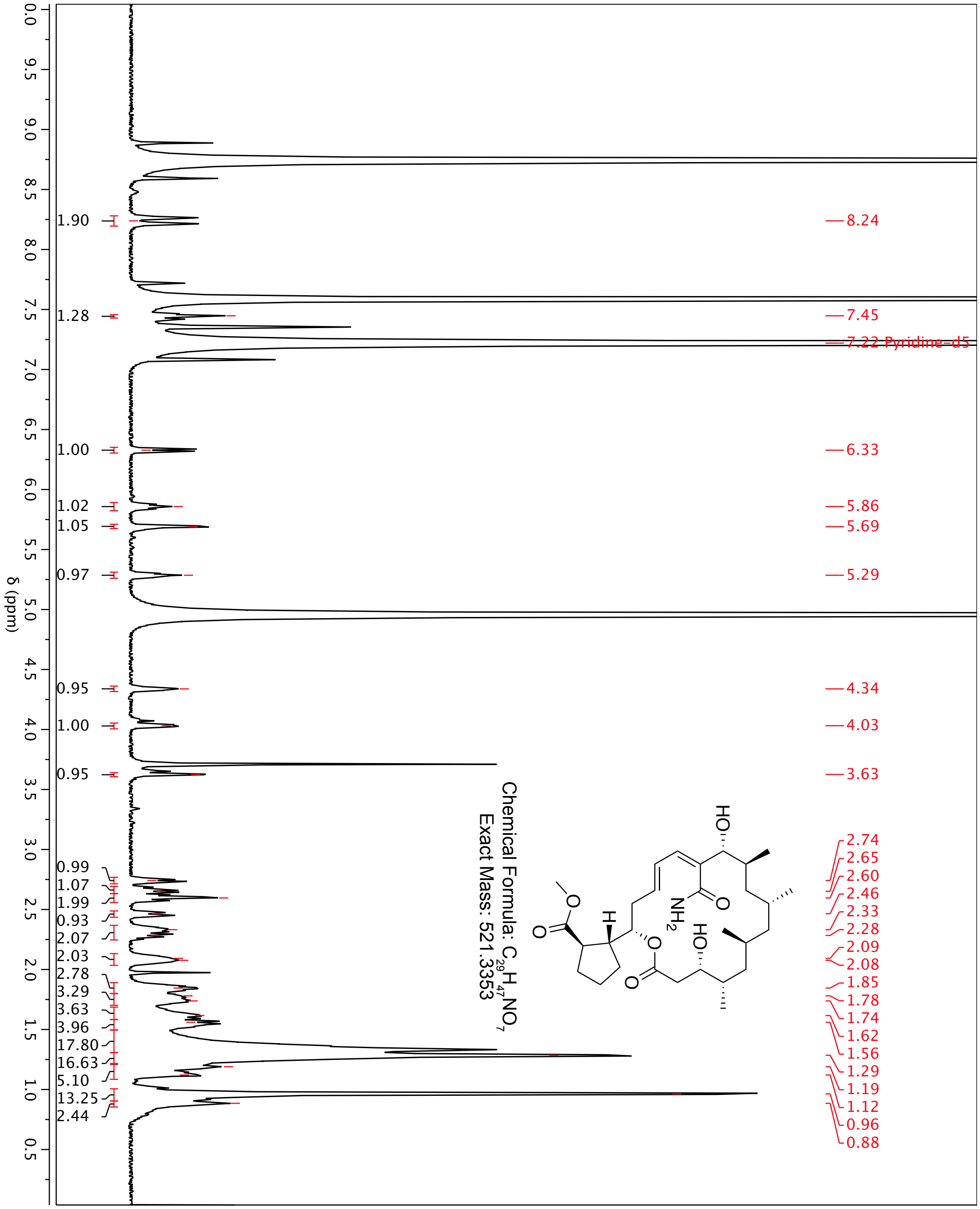

Supplementary Fig. 35 ^{1}H-NMR spectrum of borrelidin P methyl ester (**6**) at 600 MHz in pyridine-d_5

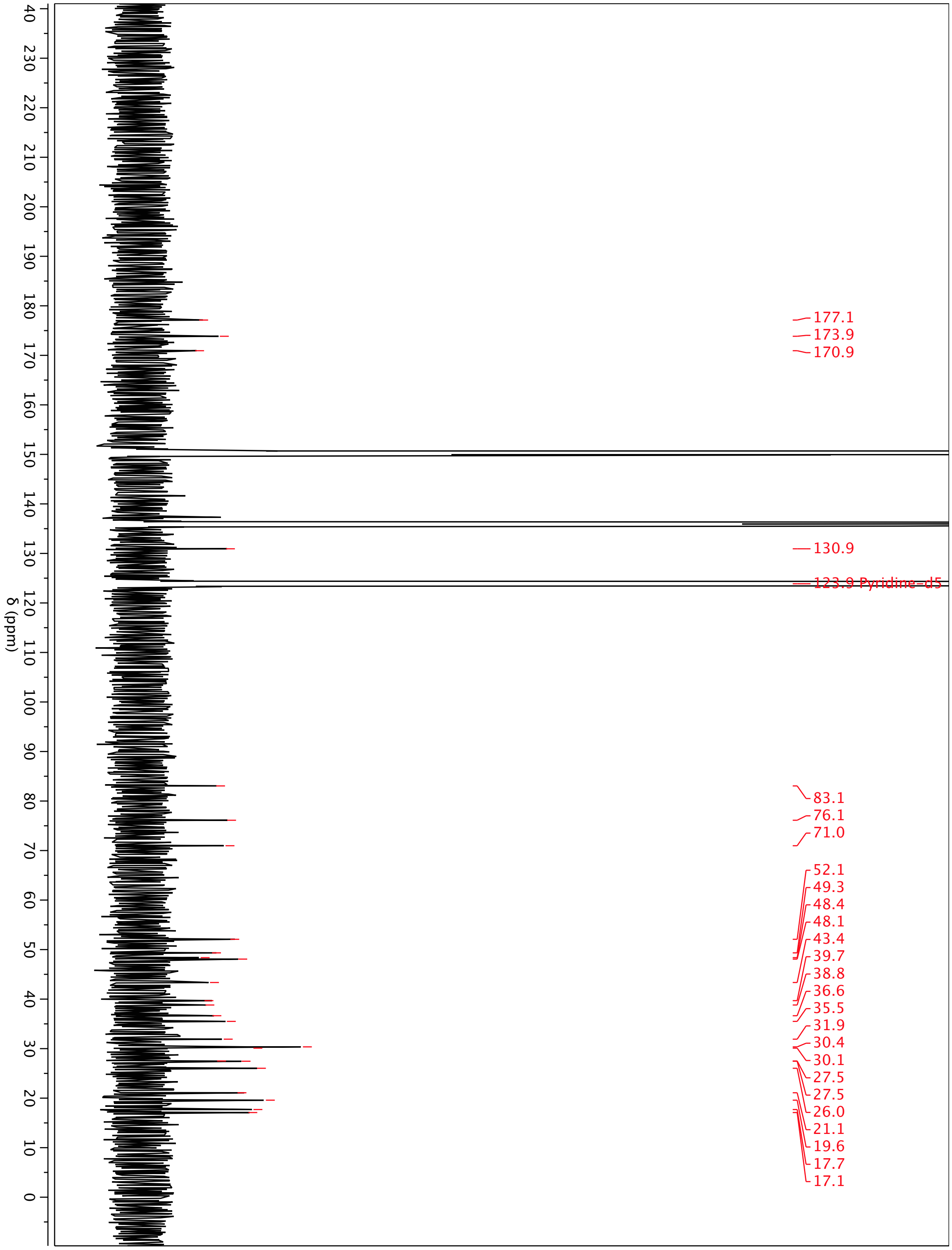

Supplementary Fig. 36 ^{13}C-NMR spectrum of borrelidin P methyl ester (**6**) at 150 MHz in pyridine-d_5

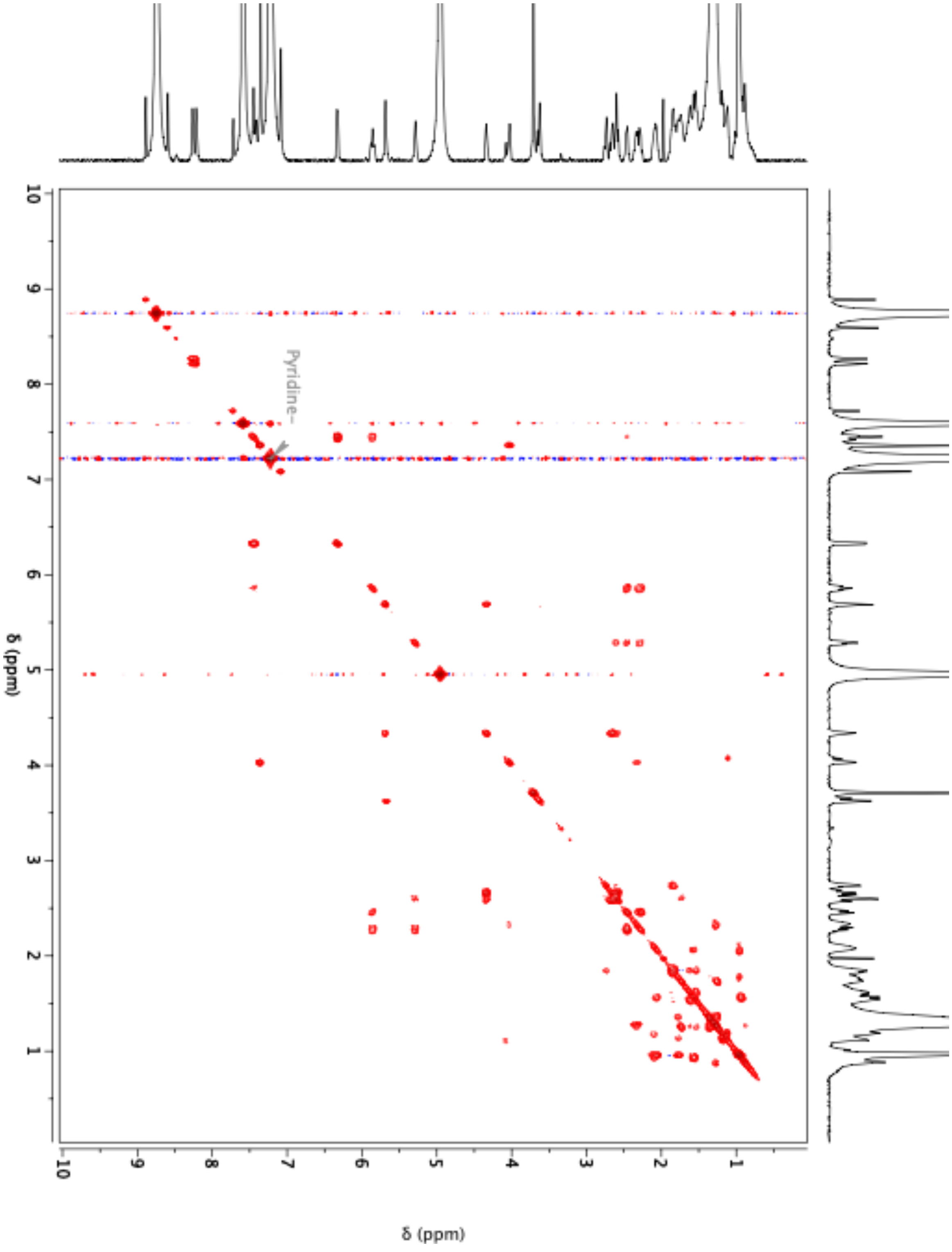

Supplementary Fig. 37 gCOSY spectrum of borrelidin P methyl ester (**6**) at 600 MHz in pyridine-d_5

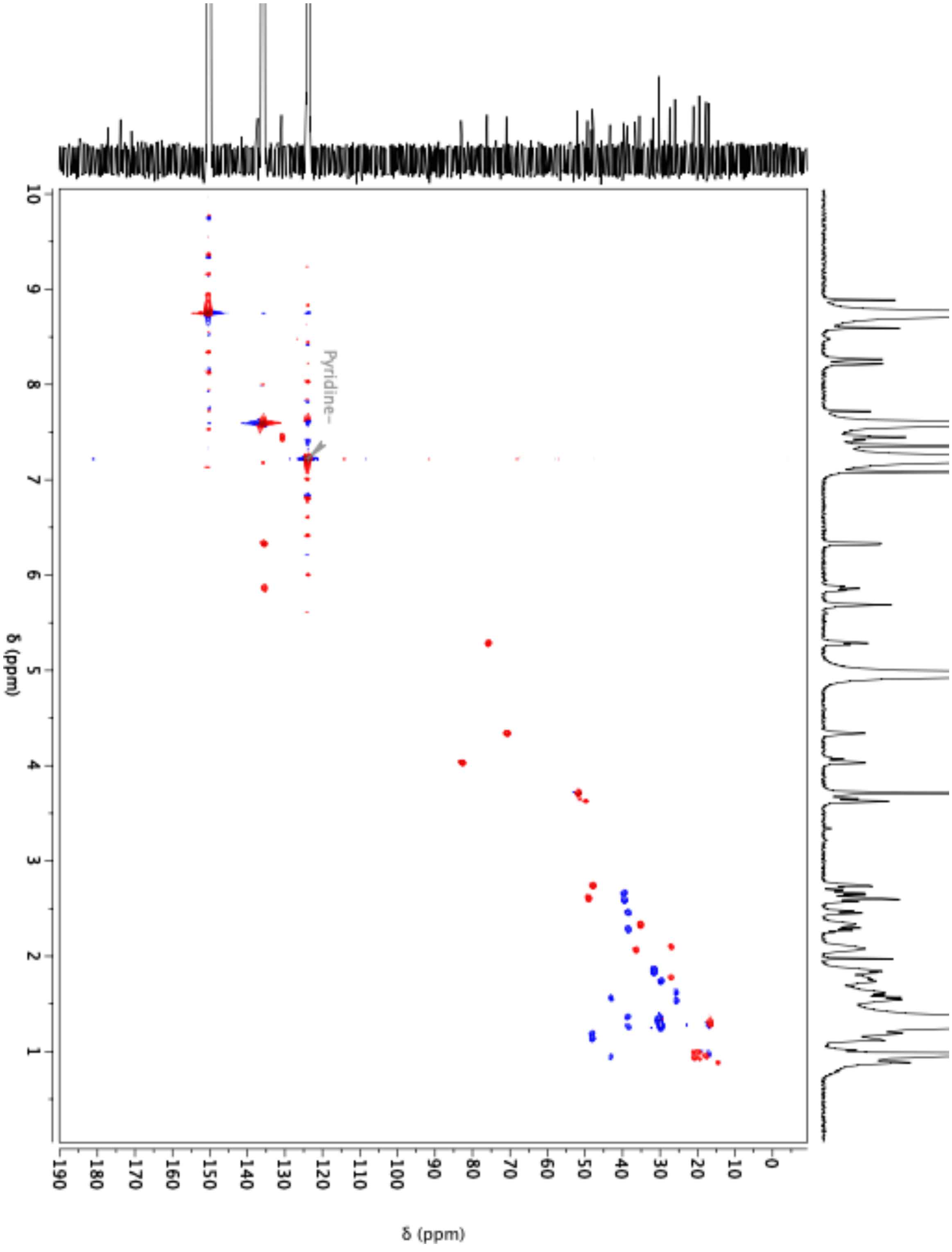

Supplementary Fig. 38 HSQC spectrum of borrelidin P methyl ester (**6**) at 600 MHz in pyridine-d_5

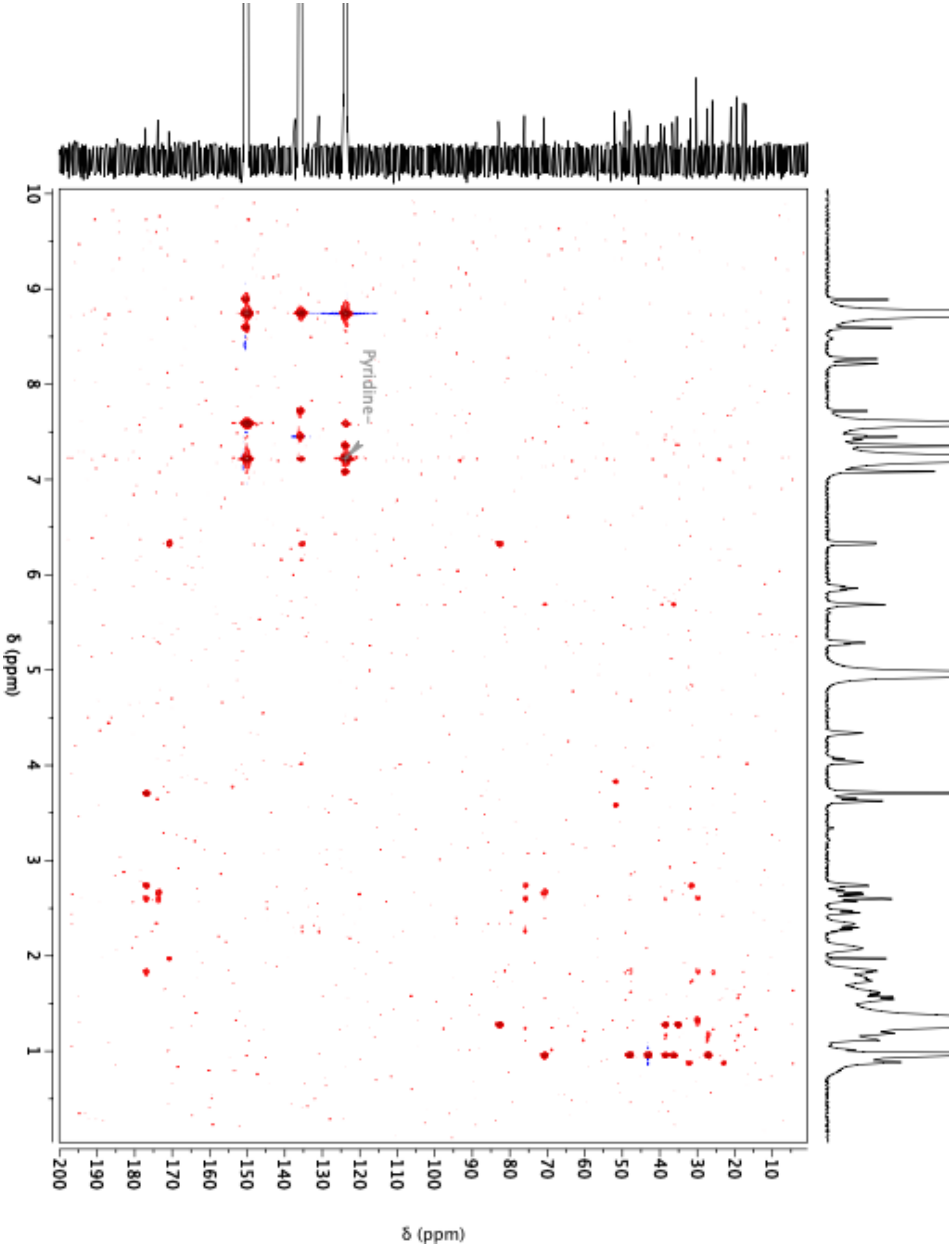

Supplementary Fig. 39 HMBC spectrum of borrelidin P methyl ester (**6**) at 600 MHz in pyridine-d_5

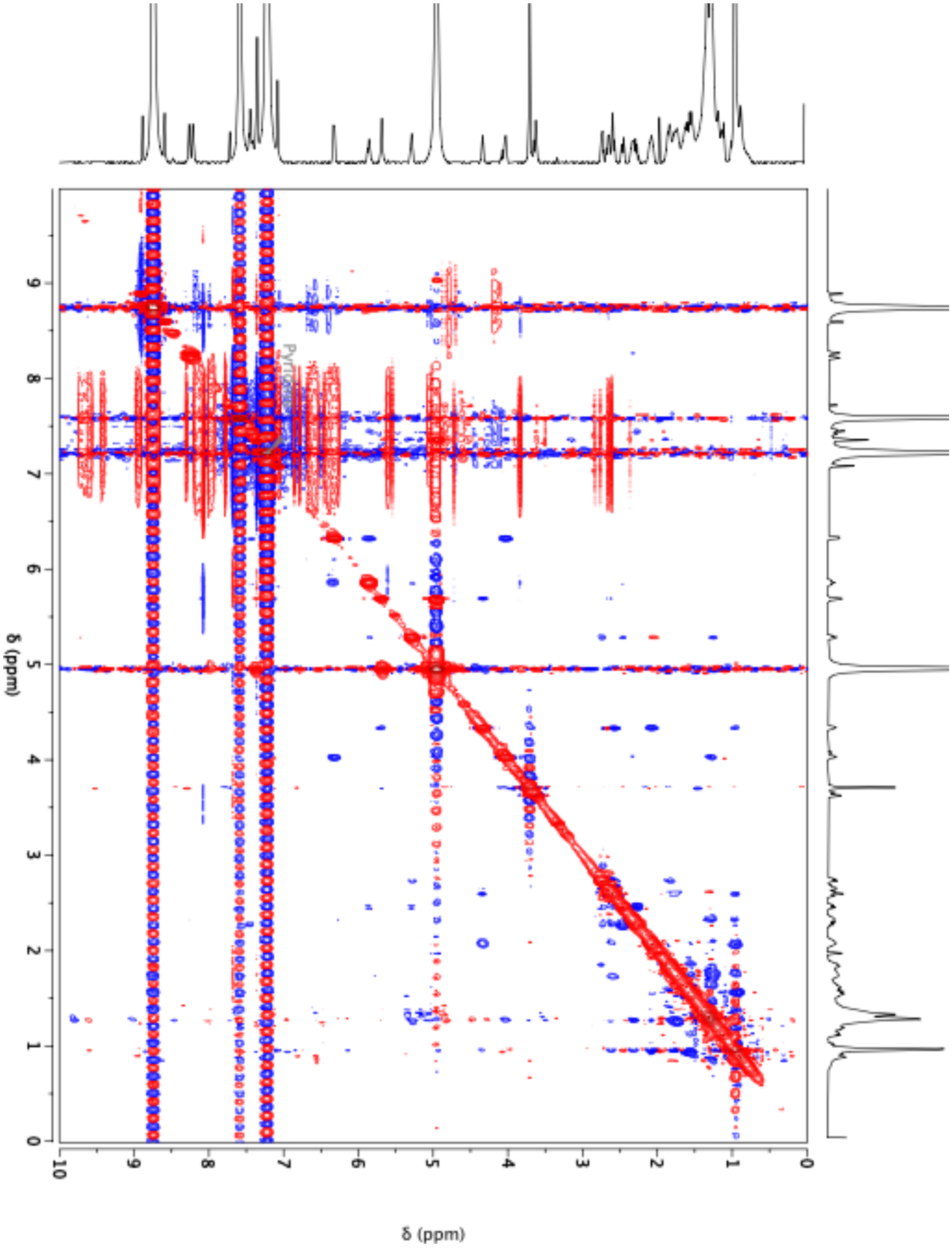

Supplementary Fig. 40 NOESY spectrum of borrelidin P methyl ester (**6**) at 600 MHz in pyridine-d_5

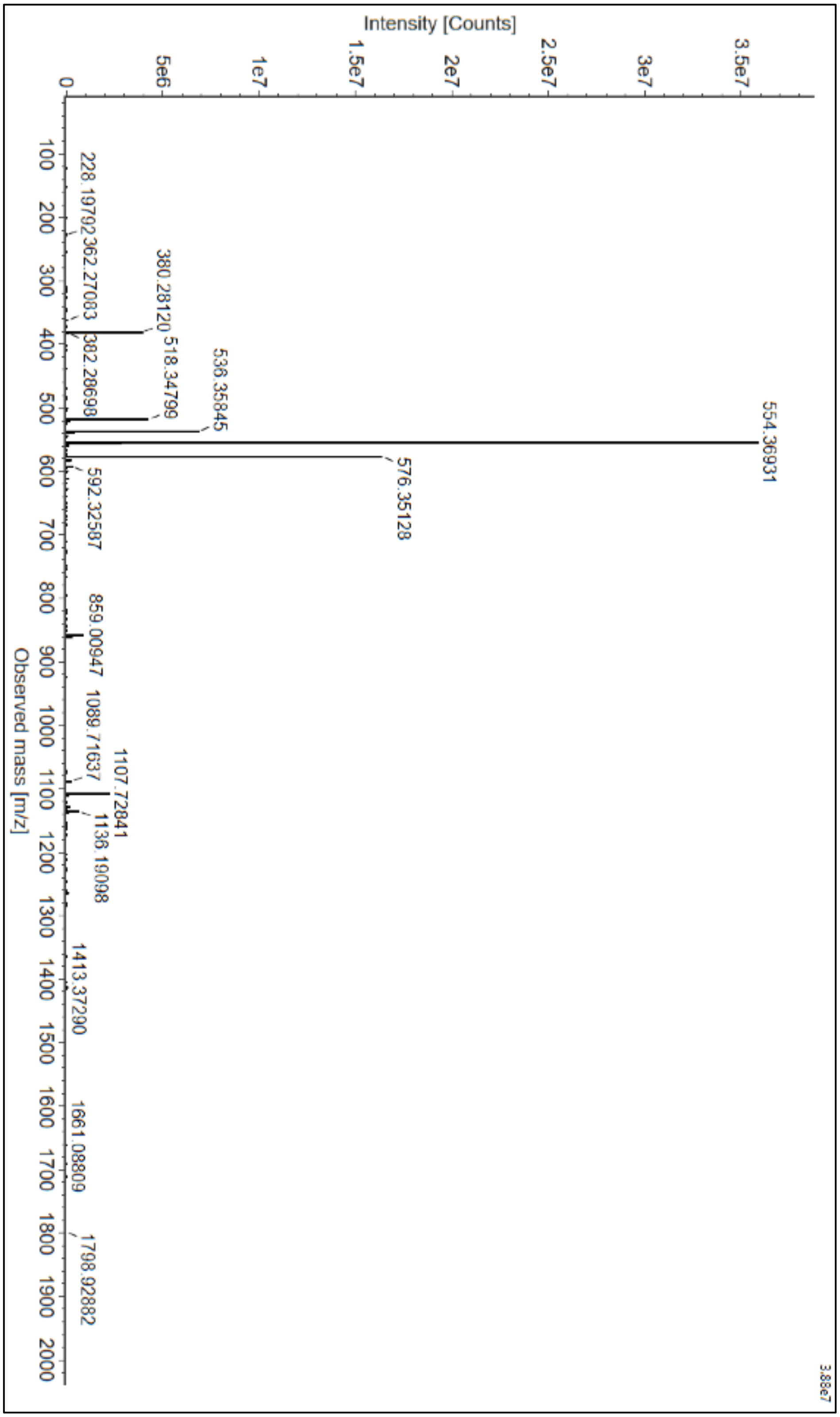

Supplementary Fig. 41 HRMS of linearized borrelidin P methyl diester (**7**)

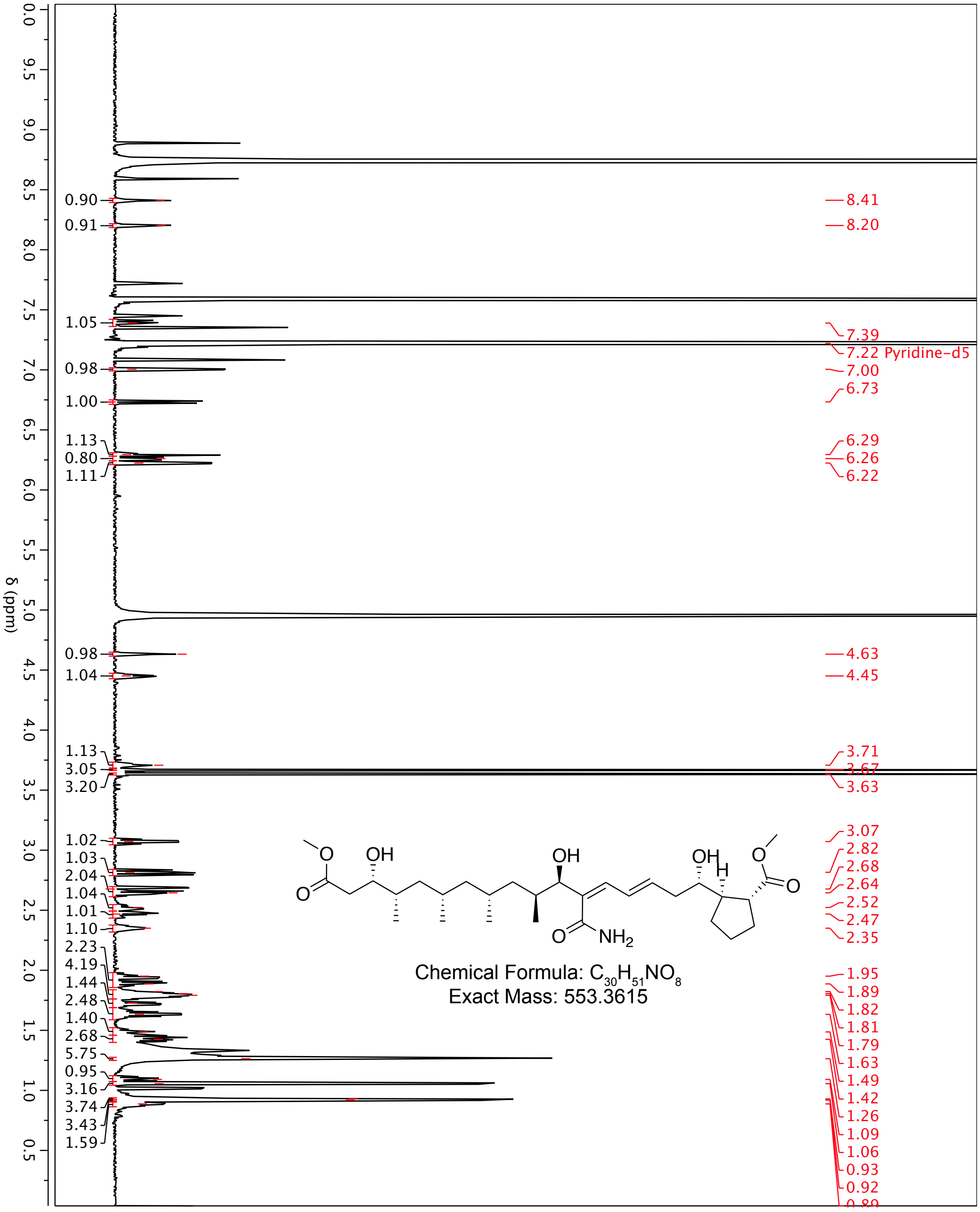

Supplementary Fig. 42 ^{1}H-NMR s spectrum of linearized borrelidin P methyl diester (**7**) at 600 MHz in pyridine-d_5

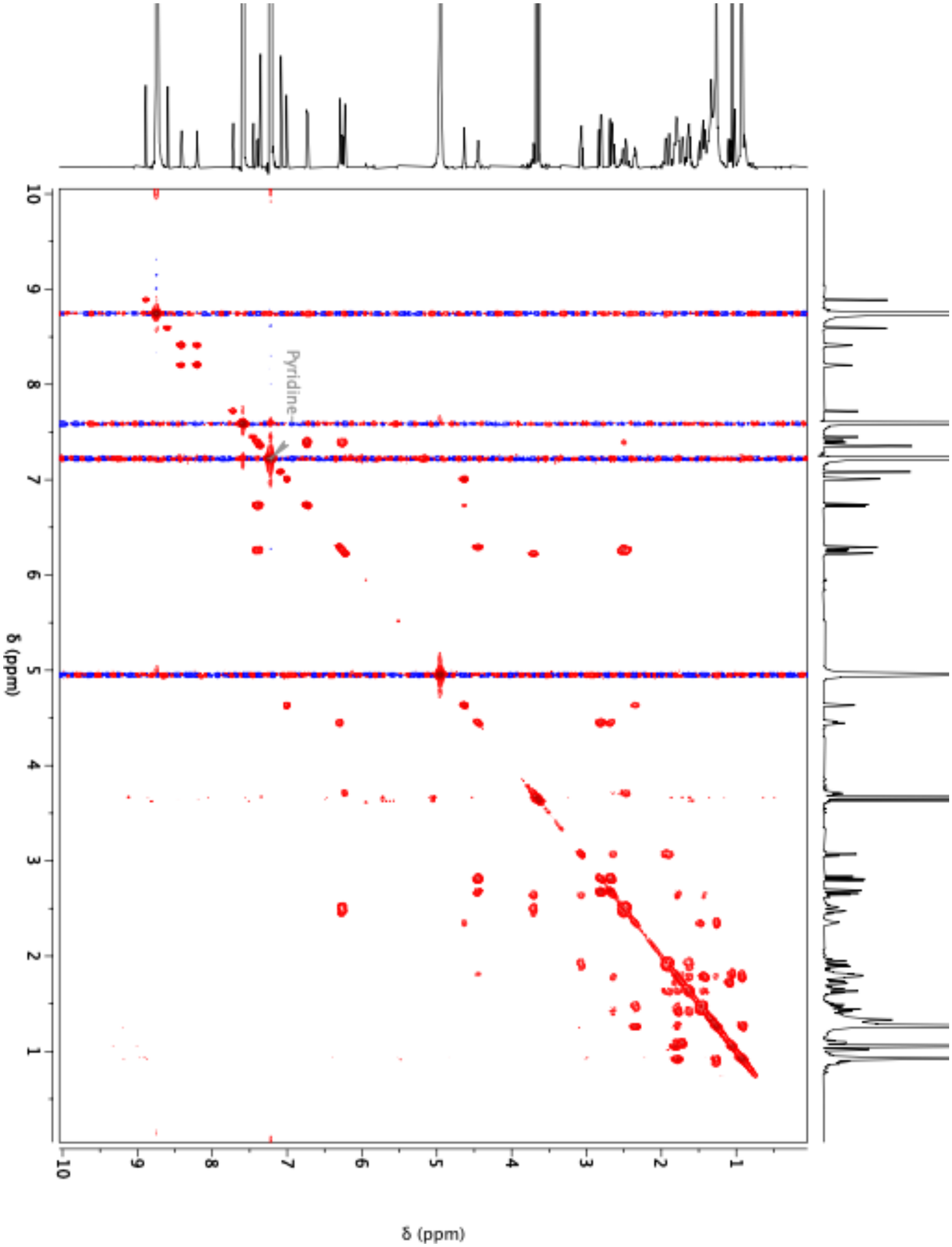

Supplementary Fig. 43 gCOSY spectrum of linearized borrelidin P methyl diester (**7**) at 600 MHz in pyridine-d_5

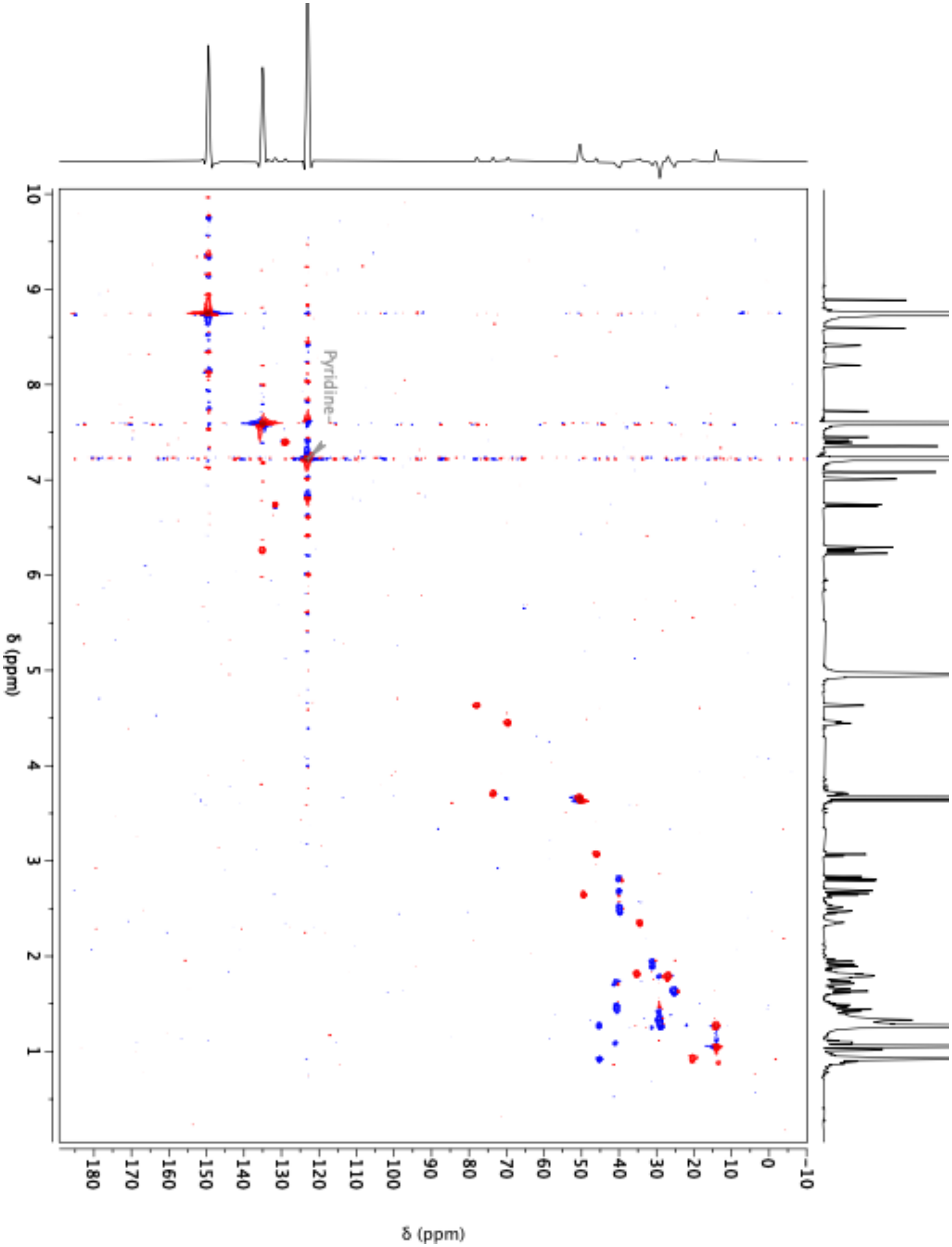

Supplementary Fig. 44 gHSQC spectrum of linearized borrelidin P methyl diester (**7**) at 600 MHz in pyridine-d_5

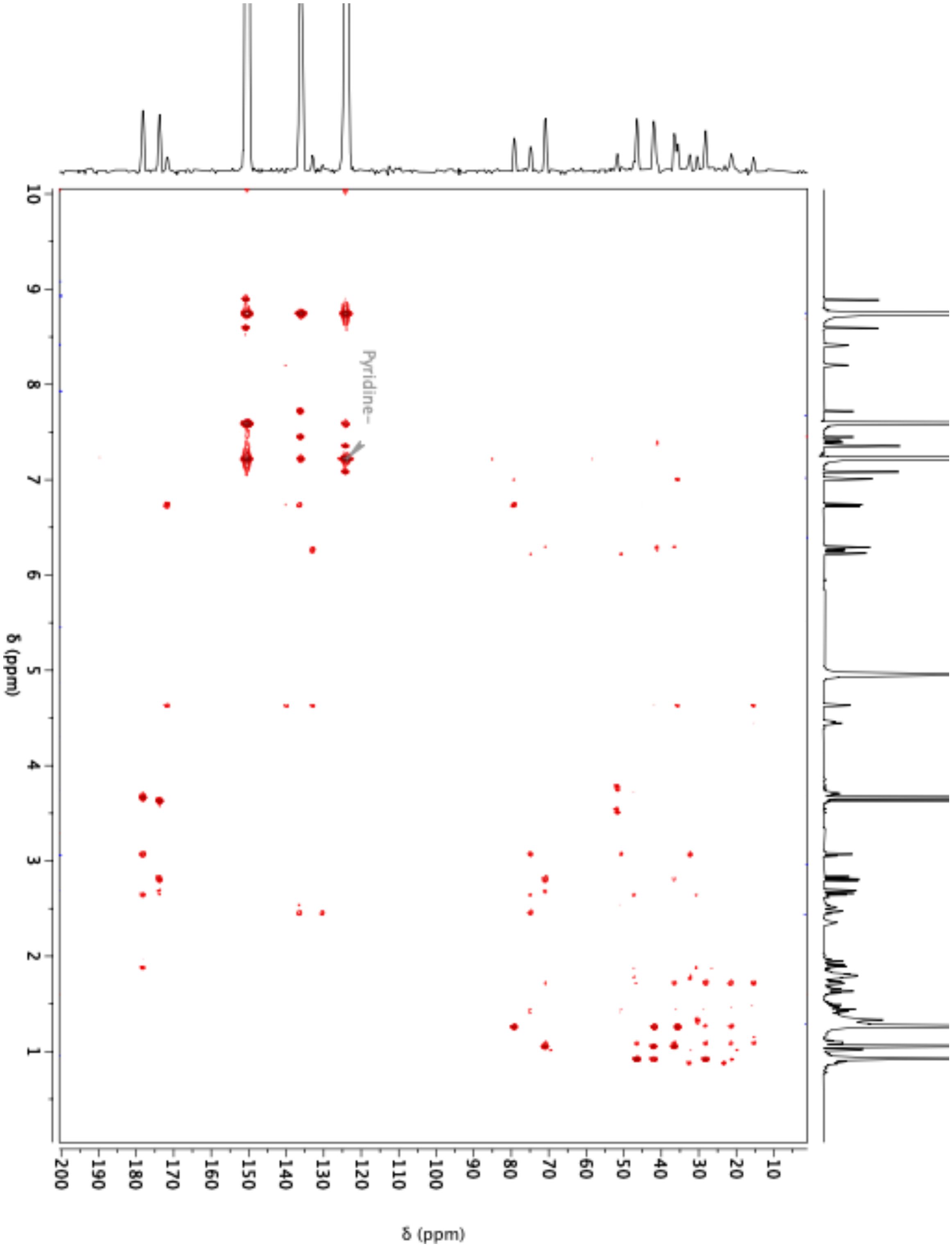

Supplementary Fig. 45 gHMBC spectrum of linearized borrelidin P methyl diester (**7**) at 600 MHz in pyridine-d_5

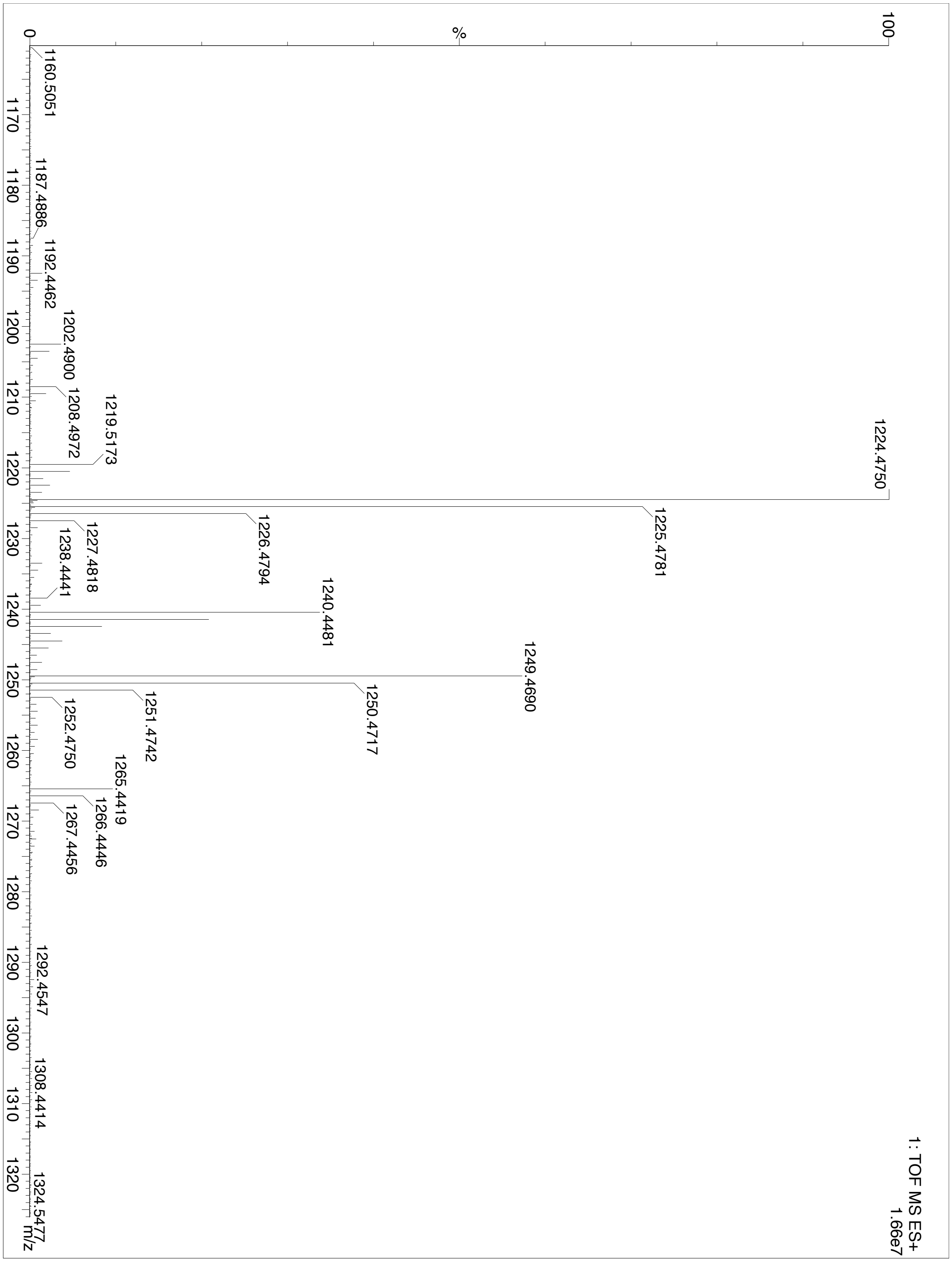

Supplementary Fig. 46 HRMS of linearized borrelidin P methyl diester (*S*)-MTPA (**8**)

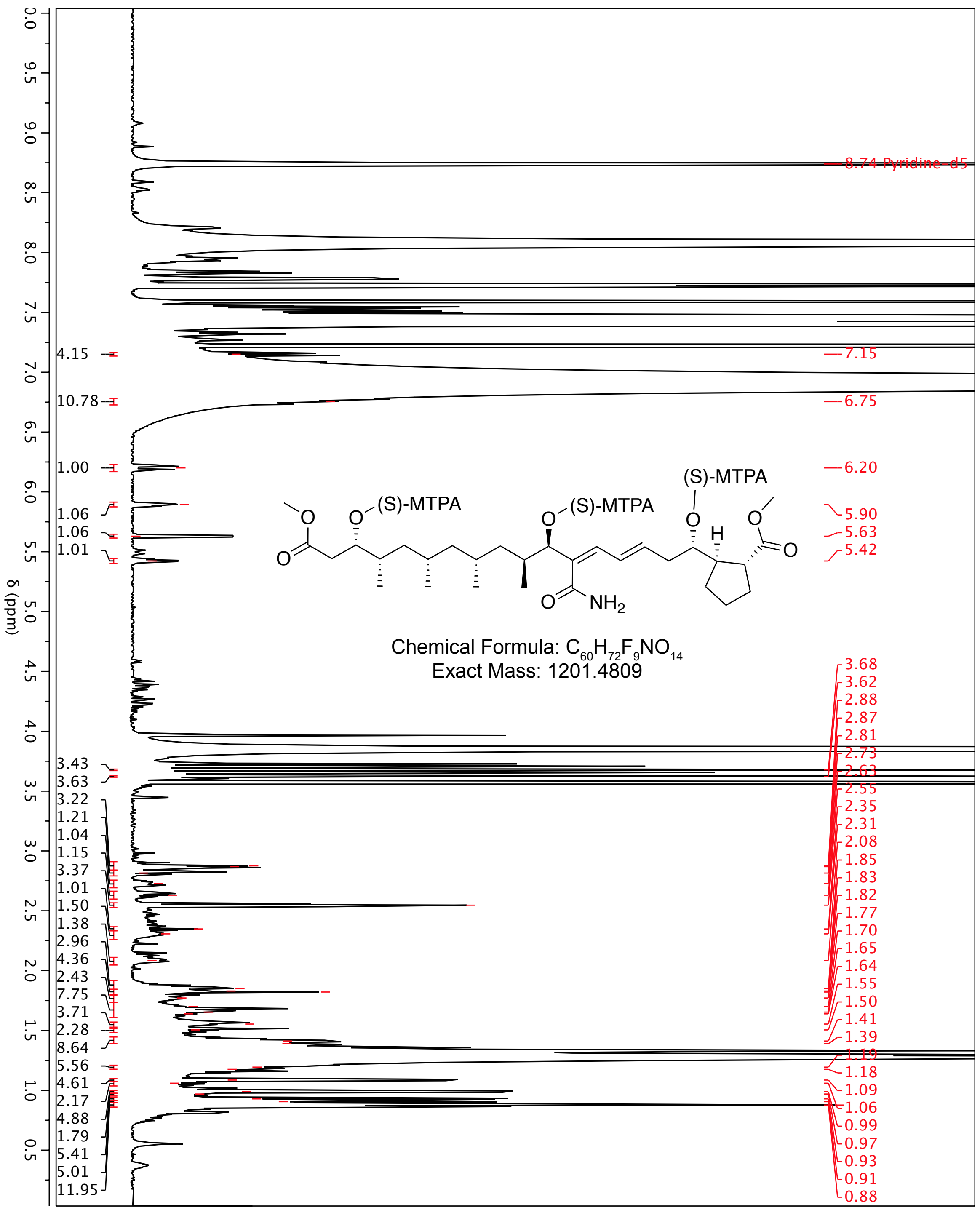

Supplementary Fig. 47 ^{1}H-NMR spectrum of linearized borrelidin P methyl diester (*S*)-MTPA (**8**) at 25 ºC, 600 MHz in pyridine-d_5

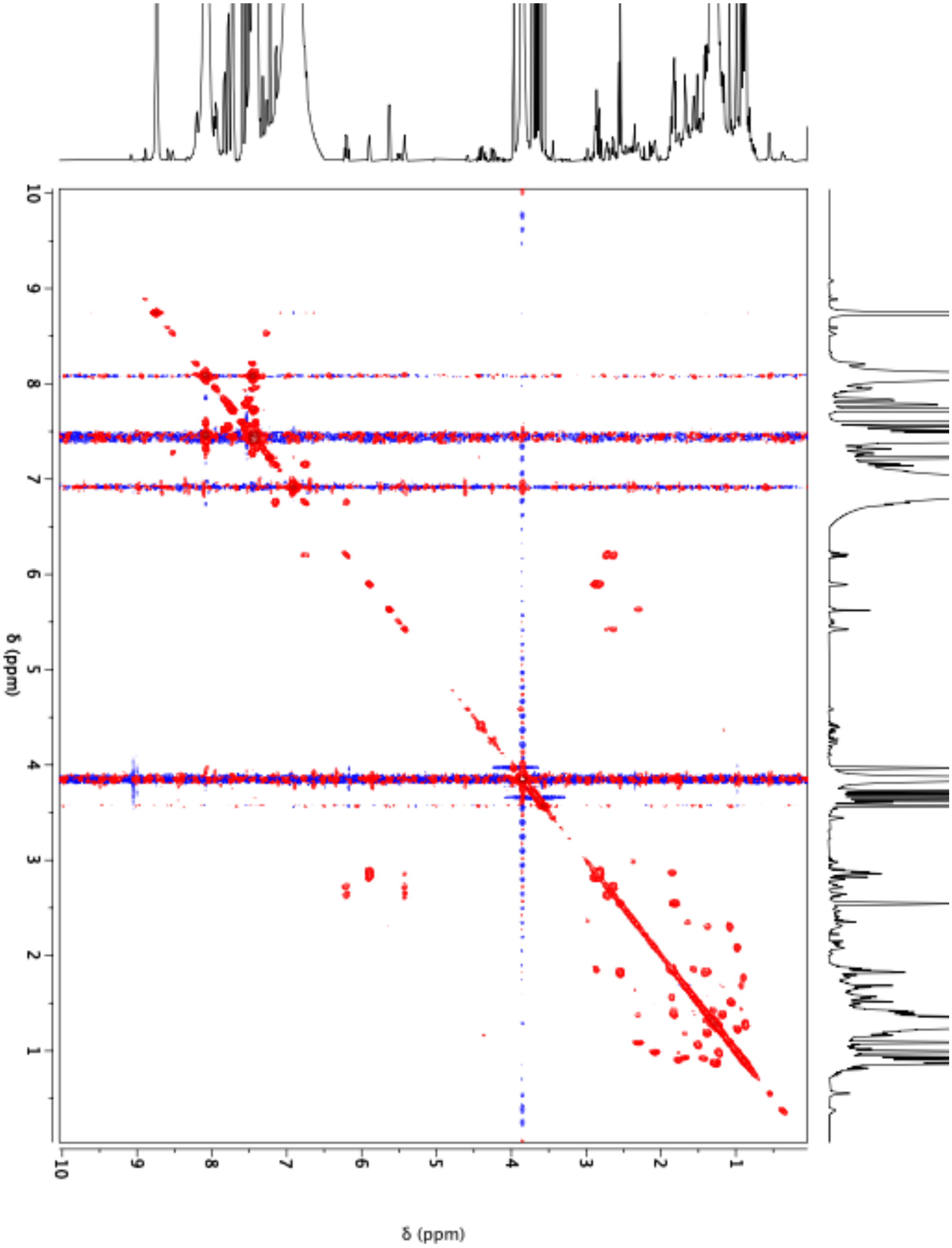

Supplementary Fig. 48 gCOSY spectrum of linearized borrelidin P methyl diester (*S*)-MTPA (**8**) at 25 ºC, 600 MHz in pyridine-d_5

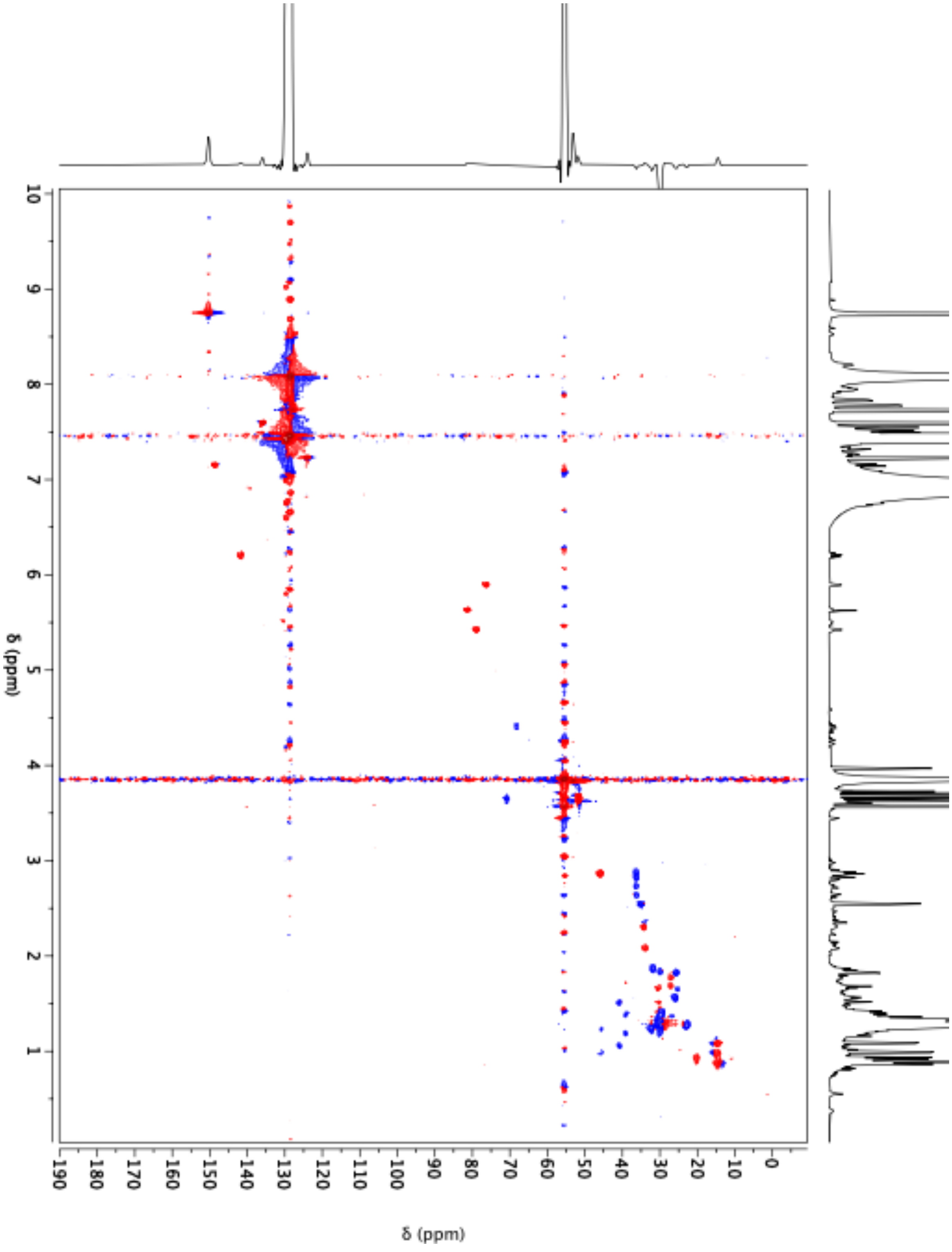

Supplementary Fig. 49 gHSQC spectrum of linearized borrelidin P methyl diester (*S*)-MTPA (**8**) at 25 ºC, 600 MHz in pyridine-d_5

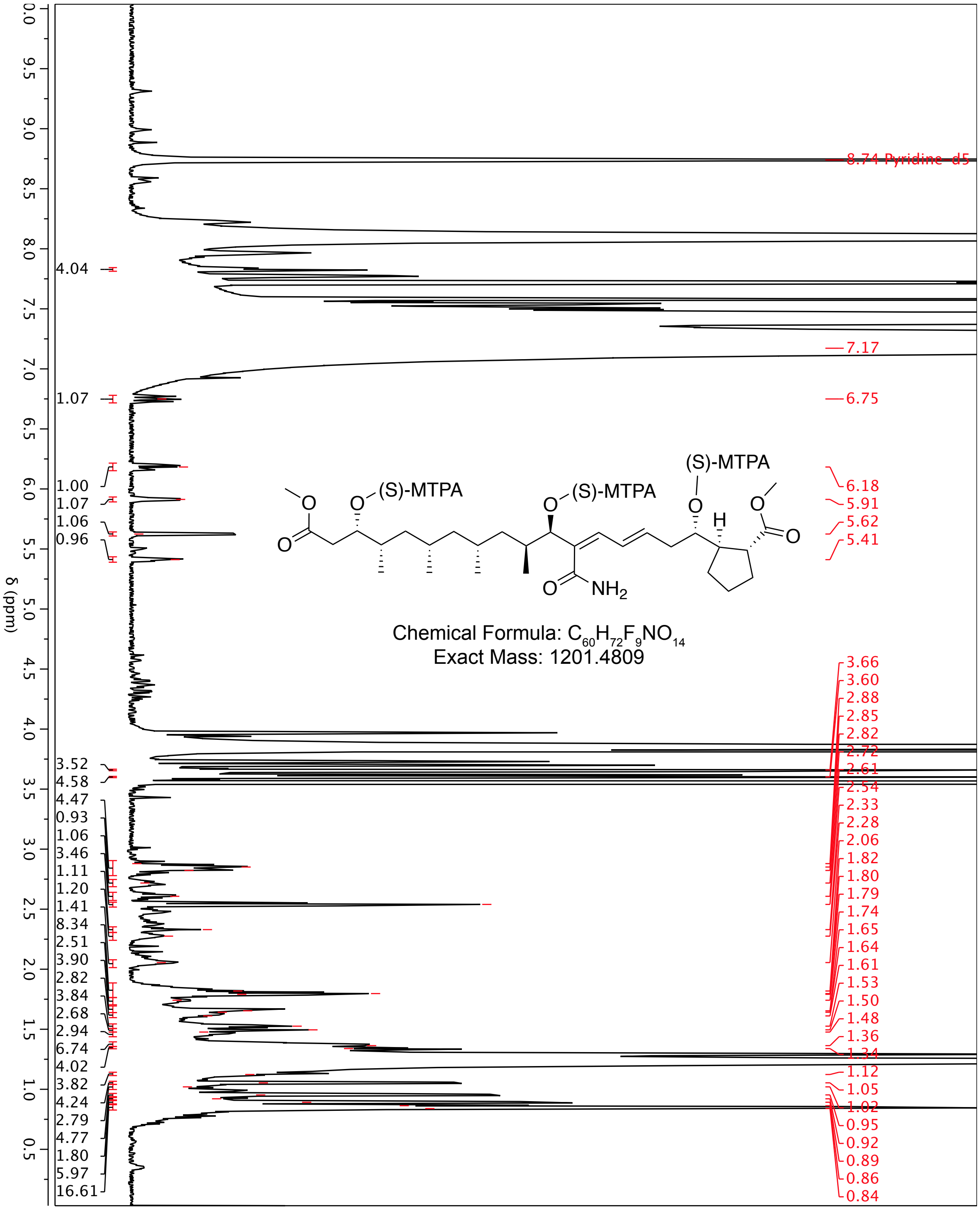

Supplementary Fig. 50 ^{1}H-NMR spectrum of linearized borrelidin P methyl diester (*S*)-MTPA (**8**) at 5 ºC, 600 MHz in pyridine-d5

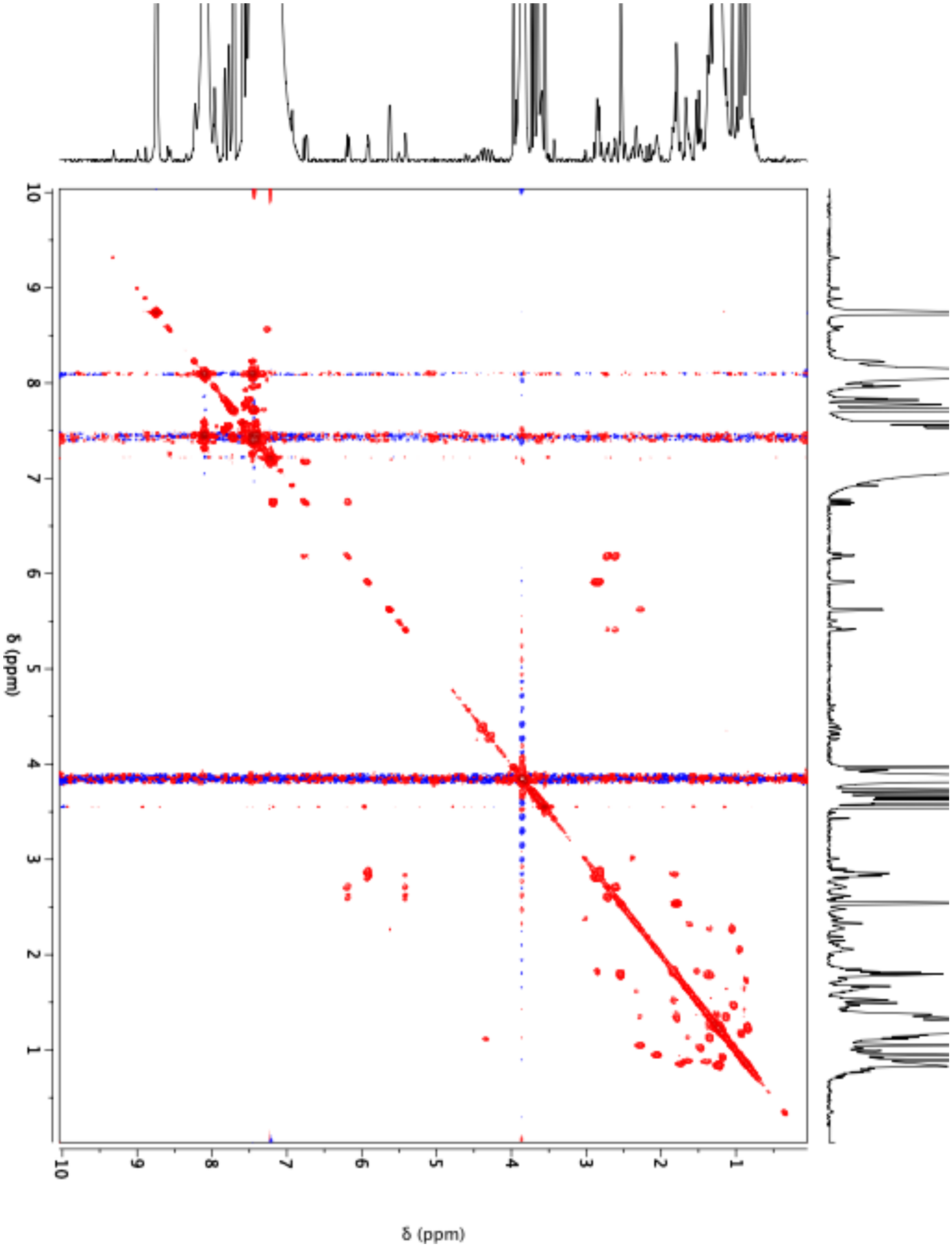

Supplementary Fig. 51 gCOSY spectrum of linearized borrelidin P methyl diester (*S*)-MTPA (**8**) at 5 ºC, 600 MHz in pyridine-d5

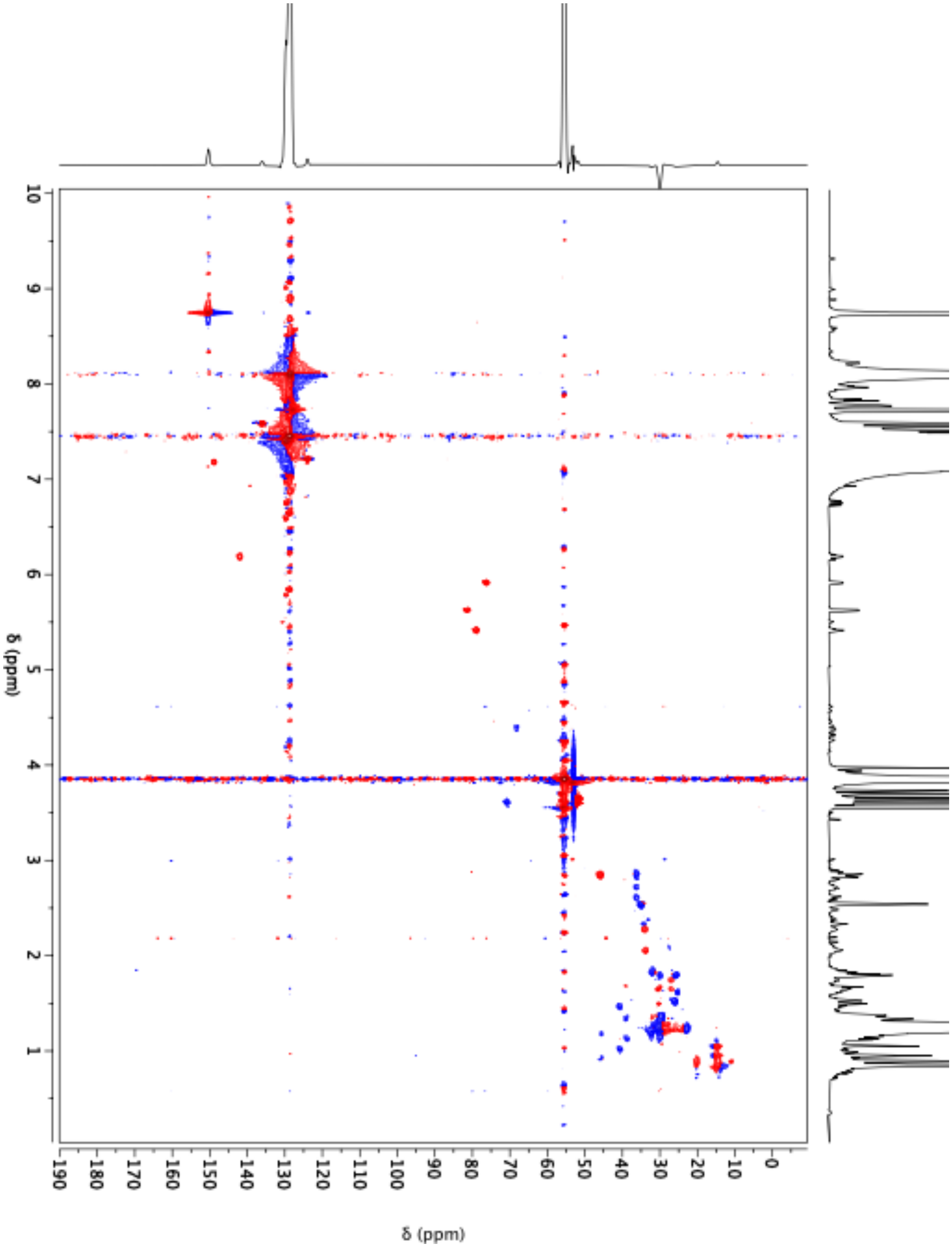

Supplementary Fig. 52 gHSQC spectrum of linearized borrelidin P methyl diester (*S*)-MTPA (**8**) at 5 ºC, 600 MHz in pyridine-d_5

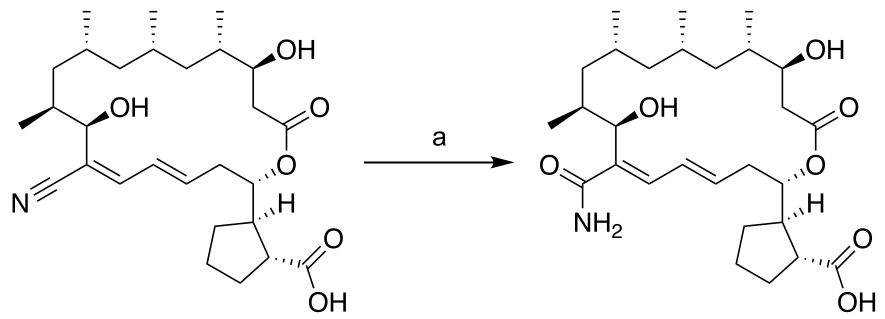

(**1**) (**5**)

Supplementary Fig. 53 Derivatization of 12-desnitrile-12-carbamoyl-borrelidin A (**5**) from borrelidin A (**1**). Reagents and conditions: (a) $CH_3CH{=}NOH$, CuO, MeOH/H_2O (1:1), 150 ºC, 60 h, 19%.

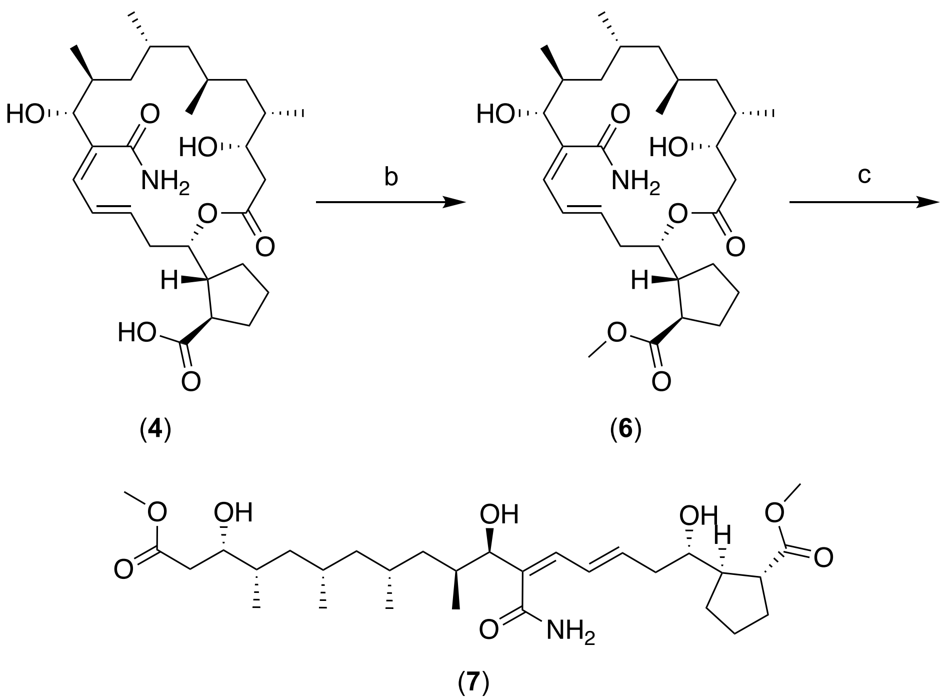

Supplementary Fig. 54 Borrelidin P esterification (**6**) and base-catalyzed ring opening (**7**). Reagents and conditions: (b) TMS(CHN_2), DCM/MeOH (1:1), 4 h, 97%; (c) 0.5 M NaOMe, MeOH, 2 h, 61%.

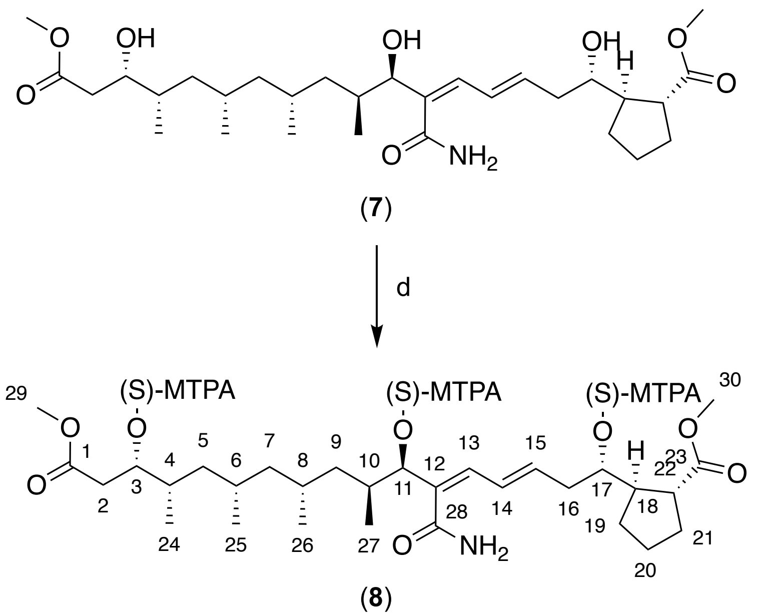

Supplementary Fig. 55 Linearized borrelidin P methyl ester (**7**) modified Mosher's ester conjugation (**8**). Reagents and conditions: (d) (*R*)-MTPA-Cl, Pyr-d_5, 15 min, ~ 99%.

Supplementary Table 1. Collateral Sensitivity Profiling target panel consists of 29 unique drug-resistant *E. coli* strains.

STRAIN	GENE	MUTATION	PROTEIN	SELECTION ANTIBIOTIC	DRUG MECH.
S83L	gyrA	S83L	DNA gyrase subunit A	Ciprofloxacin	DNA gyrase
Cip1		S83A			
Cip3		D87Y			
Cip5		D87G			
Cip2KB	gyrB	L509G	DNA gyrase subunit B		
Cip15KB		S464Y			
NA3		D426N		Nalidixic Acid	
Cip8	marR	R77H	Multiple antibiotic resistance protein	Ciprofloxacin	Multidrug resistance
Tet8		H120fs		Tetracycline	
Cm2	acrR	E118*	HTH-type transcriptional regulator AcrR	Chloramphenicol	
Cm3		A151fs			
Rif1	rpoB	I572L	DNA-directed RNA polymerase subunit beta	Rifampicin	RNA polymerase
Rif7		I572S			
Rif11		D516N			
Cef1	envZ	T402M	Sensor histidine kinase EnvZ	Ceftazidime	Cell wall biosynthesis
Cef8		P248S			
Cef6	rfaH	W4*	Transcription antitermination protein RfaH		
Cef7	rfaG	E289fs	Lipopolysaccharide core biosynthesis protein RfaG		
Gn12	cyoA	I127fs	Cytochrome bo(3) ubiquinol oxidase subunit 2	Gentamicin	Protein synthesis
Kn14		W82*		Kanamycin	
Kn6	ubiB	Y176*	Probable protein kinase UbiB		
Gn14	ubiF	D342H	3-demethoxyubiquinol 3-hydroxylase	Gentamicin	
Kn15		Q120*		Kanamycin	
Str1	rpsL	K43R	30S ribosomal protein S12	Streptomycin	
Str3		K43N			
Str4		P91Q			
RK2	N/A	N/A	N/A	Kanamycin	Plasmid-borne multidrug resistance
AC29-1	N/A	N/A	N/A	Ampicillin	
AC30-1	N/A	N/A	N/A		

Supplementary Table 2. 80-member antimicrobial control library.

#	NAME	MANUFACTURER	CATALOG #	STRUCTURAL CLASS	DRUG TARGET
1	Penicillin G	Fisher BioReagents	BP914-100	Penicillin	Cell Wall Synthesis
2	Amoxicillin	MP Biomedicals	190145		
3	Piperacillin Na^+	MP Biomedicals	R25559-1GM		
4	Ampicillin	Fisher BioReagents	BP1760-5		
5	Cloxacillin Na^+ H_2O	Sigma-Aldrich	C9393-16		
6	Carbenicillin $2Na^+$	Research Products International	46000-5.0		
7	Cephadroxil	Sigma-Aldrich	C7020-16	Cephalosporin	
8	Cefaclor	Santa Cruz Biotech	SC-292532		
9	Ceftazidime H_2O	Sigma-Aldrich	C3809-16		
10	Vancomycin HCl	Alfa-Aesar	J62790	Glycopeptide	
11	Polymixin B H_2SO_4	Research Products International	P40160-5.0	Oligopeptide	
12	Bacitracin Zn^{2+}	Sigma-Aldrich	B5150-250KU		
13	Alafosfalin	Sigma-Aldrich	05260-250MG	Phosphonodipeptide	
14	D-Cycloserine	Sigma-Aldrich	C6880-16	Peptide	
15	Bafilomycin B1	Sigma-Aldrich	11707-1MG	Macrolide	Ionophore
16	Amphotericin B	Sigma-Aldrich	A2411-250MG		
17	Nystatin	Cayman Chemical Company	475914-5GM		
18	Nonactin	Sigma-Aldrich	N2286-5MG		
19	Monensin Na^+	Sigma-Aldrich	M5273-16	Polyether	
20	Salinomycin	Sigma-Aldrich	S4526-5MG		
21	Daptomycin	Tocris Bioscience	3917	Lipopeptide	
22	Tyrothricin	MP Biomedicals	100560	Oligopeptide	
23	Gramicidin	Sigma-Aldrich	55002-16		
24	Valinomycin	MP Biomedicals	105010	Depsipeptide	
25	Nalidixic Acid	Fisher BioReagents	FL-05-0106	Quinolone	DNA Replication
26	Levofloxacin	Sigma-Aldrich	28266-16-F	Fluoroquinolone	
27	Ciprofloxacin	Sigma-Aldrich	17850-56-F		
28	Norfloxacin	MP Biomedicals	155949		
29	Sparfloxacin	Sigma-Aldrich	56968-16-F		
30	Doxorubicin HCl	MP Biomedicals	159101	Anthracycline	
31	Epirubicin HCl	MP Biomedicals	195984		
32	Idarubicin HCl	Sigma-Aldrich	I1656-10MG		
33	Mithramycin	Sigma-Aldrich	M6891-1MG		
34	Actinomycin D	Sigma-Aldrich	A1410-10MG	Depsipeptide	
35	Novobiocin Na^+	Promega	C946A	Aminocoumarin	
36	Streptonigrin	Sigma-Aldrich	S1014-1MG	Aminoquinone	
37	Netropsin 2HCl	Sigma-Aldrich	N9653-5MG	Polyamide	
38	Nitrofurantoin	Sigma-Aldrich	N7878-10G	Nitrofuran	

39	Furazolidone	MP Biomedicals	193465		
40	Ornidazole	MP Biomedicals	155999	Nitroimidazole	
41	Sulfamethazine	Sigma-Aldrich	S6*-25G	Sulfonamide	DNA/RNA Synthesis
42	Sulfapyridine	Sigma-Aldrich	S6252-25G		
43	Sulfamethoxazole	Wako Chemicals	199-10451		
44	Sulfadiazine	MP Biomedicals	156706		
45	Sulfamerazine	Sigma-Aldrich	S8876-506		
46	Rifampicin	Fisher BioReagents	BP2679250	Rifamycin	RNA Synthesis
47	Rifabutin	Cayman Chemical Co.	16468		
48	Rifaximin	Sigma-Aldrich	33999		
49	Resistomycin	Toronto Research Chemicals	R144683	Resistomycin	
50	Resistoflavine	Toronto Research Chemicals	R144688		
51	Oxytetracycline HCl	Sigma-Aldrich	O5575-10G	Tetracycline	Protein Synthesis
52	Tetracycline HCl	Sigma-Aldrich	T7660-5G		
53	Doxycycline HCl	Fisher BioReagents	BP26535		
54	Demeclocycline HCl	Sigma-Aldrich	PHR 1735-16		
55	Minocycline HCl	Sigma-Aldrich	M9511-100MG		
56	Tobramycin	Sigma-Aldrich	PHR 1079-1G	Aminoglycoside	
57	Gentamicin H_2SO_4	Sigma-Aldrich	G1264-1G		
58	Kanamycin H_2SO_4	Fisher BioReagents	DP906-5		
59	Amikacin	MP Biomedicals	150342		
60	Streptomycin H_2SO_4	Sigma-Aldrich	S-9137		
61	Spectinomycin H_2SO_4	Sigma-Aldrich	PHR 1441-1G		
62	Erythromycin	Sigma-Aldrich	856193-5G	Macrolide	
63	Clarithromycin	Sigma-Aldrich	PHR 1038-500MG		
64	Midecamycin	Alfa-Aesar	J66046		
65	Azithromycin $2H_2O$	Tokyo Chemical Industry	A2076		
66	Roxithromycin	Sigma-Aldrich	R4393-1G		
67	Spiramycin	Sigma-Aldrich	S9132-1G		
68	Thiamphenicol	Spectrum Chemical	T3077	Amphenicol	
69	Chloramphenicol	CalBioChem	220551		
70	Florfenicol	Santa Cruz Biotech	SC-205696		
71	Clindamycin PO_4	MP Biomedicals	158892	Lincomycin	
72	Lincomycin HCl	MP Biomedicals	158948		
73	Cycloheximide	Sigma-Aldrich	C7698-5G		
74	Thiostrepton	EMD Millipore	598226-1GM	Thiopeptide	
75	Puromycin 2HCl	Sigma-Aldrich	P7255-25MG	Aminonucleoside	
76	Tiamulin $C_4H_4O_4$	Sigma-Aldrich	46959-100MG-F	Pleuromutilin	
77	Staurosporine	Cayman Chemical Co.	81590-1	Bis-indole	Protein Kinase

78	Antimycin A	Sigma-Aldrich	A8674-25MG	Macrolide	Electron Transport Chain
79	Tunicamycin	Sigma-Aldrich	T7765-10MG	Nucleoside	Glycoprotein Synthesis
80	Cyclosporin A	Santa Cruz Biotech	SC-3503	Oligopeptide	Cytokine Transcription

Supplementary Table 3. Z'-factor and Z-factor values for CSP screening of the antimicrobial library and the natural product library, respectively.

STRAIN	ANTIMICROBIAL SCREEN (Z')	NATURAL PRODUCTS SCREEN (Z)
MG1655 (WT)	0.758	0.748
S83L	0.804	0.752
Cip1	0.770	0.743
Cip3	0.765	0.728
Cip5	0.761	0.734
Cip2KB	0.717	0.614
Cip15KB	0.710	0.658
NA3	0.811	0.595
Cip8	0.750	0.743
Tet8	0.794	0.649
Cm2	0.762	0.599
Cm3	0.785	0.635
Rif1	0.479	0.389
Rif7	0.790	0.679
Rif11	0.730	0.619
Cef1	0.375	0.378
Cef8	0.453	0.526
Cef6	0.686	0.513
Cef7	0.611	0.391
Gn12	0.740	0.364
Kn14	0.792	0.342
Kn6	0.671	0.493
Gn14	0.737	0.334
Kn15	0.773	0.351
Str1	0.739	0.482
Str3	0.648	0.446
Str4	0.635	0.453
RK2	0.676	0.504
AC29-1	0.756	0.548
AC30-1	0.776	0.540
AVERAGE	0.709	0.552

$$\text{Z}'\text{-factor} = 1 - \frac{3 \times \sigma_{pos} + 3 \times \sigma_{neg}}{\left|\mu_{pos} - \mu_{neg}\right|}$$

$$\text{Z-factor} = 1 - \frac{3 \times \sigma_{pos} + 3 \times \sigma_{all}}{\left|\mu_{pos} - \mu_{all}\right|}$$

Where σ_{pos} is the standard deviation of the positive control, σ_{neg} is the standard deviation of the negative control, μ_{pos} is the average of the positive control, μ_{neg} is the average of the negative control, σ_{all} is the standard deviation of the entire library, and μ_{all} is the average of the entire library.

Supplementary Table 4. Tabulated NMR data for (**1**) and (**4**) in methanol-d_4 at 600 MHz and 150 MHz for ^{1}H- and ^{13}C-NMR, respectively.

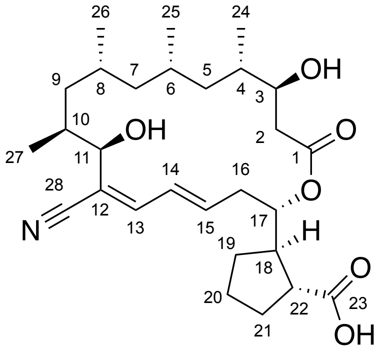

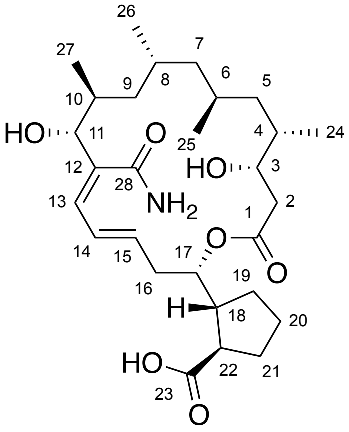

Position	(**1**) δ_H (*J* in Hz)	(**1**) δ_C	(**4**) δ_H (*J* in Hz)	(**4**) δ_C
1		173.3		174.7
2	2.24, dd (16.0, 10.2)<br>2.40, d (14.8)	37.9	2.40, d (6.0)	40.6
3	3.94, d (8.8)	73.1	3.84, td (6.0, 3.1)	71.6
4	1.86, overlap	37.1	1.76, overlap	37.0
5	0.99, overlap<br>1.19, t (11.8)	44.6	0.85, 1.39, overlap	44.0
6	1.88, overlap	28.5	1.71, overlap	28.1
7	0.97, overlap<br>1.09, ddd (14.0, 11.4, 2.8)	49.4	1.07, m<br>1.14, ddd (13.03, 9.8, 3.9)	49.0
8	1.63, overlap	27.6	1.64, overlap	27.8
9	0.70, t (12.2)<br>1.24, t (12.4)	39.1	0.95, 1.03, overlap	38.8
10	1.81, overlap	35.9	1.85, overlap	35.9
11	4.19, d (9.8)	72.9	3.74, d (8.6)	83.0
12		117.4		135.5
13	6.92, d (11.3)	145.5	6.19, d (11.0)	136.4
14	6.61, dd (14.2, 11.8)	128.9	6.72, dd (14.8, 11.5)	130.0
15	6.33, ddd (14.8, 10.4, 4.6)	140.3	5.82, ddd (14.7, 10.0, 4.2)	137.9
16	2.53, d (15.0),<br>2.59, ddd (14.6, 10.5, 4.1)	36.7	2.36, 2.57, overlap	39.1
17	4.98, d (10.2)	77.5	5.06, t (8.6)	77.7
18	2.67, dt (17.2, 9.0)	47.5	2.53, overlap	49.5
19	1.42, m<br>2.01, overlap	30.5	1.37, 1.87, overlap	30.6
20	1.78, overlap	26.2	1.63, 1.74, overlap	26.3
21	1.82, 2.02, overlap	32.6	1.74, 1.96, overlap	33.1
22	*	*	*	*
23		*		183.6

Position	(**1**) δ_H (*J* in Hz)	(**1**) δ_C	(**4**) δ_H (*J* in Hz)	(**4**) δ_C
24	0.87, d (6.1)	18.7	0.86, d (6.6)	16.7
25	0.87, d (6.1)	19.1	0.82, d (6.8)	19.3
26	0.85, d (6.1)	20.9	0.85, d (6.7)	20.8
27	1.04, d (6.4)	15.4	0.98, d (6.3)	16.5
28		119.9		172.6

* Not Observed

Supplementary Table 5. Tabulated ^{1}H-NMR shifts for linearized Borrelidin P methyl ester (**7**) and (S)-MTPA conjugated product (**8**) under two different temperatures at 600 MHz in pyridine-d_5. The structure of (**8**) is labelled with $\Delta\delta_H^{T1,T2}$ values from the variable temperature modified Mosher's ester method. Shielding from the phenyl ring in the suggested major conformer displayed below causes the affected protons to be shifted upfield. * = not observed.

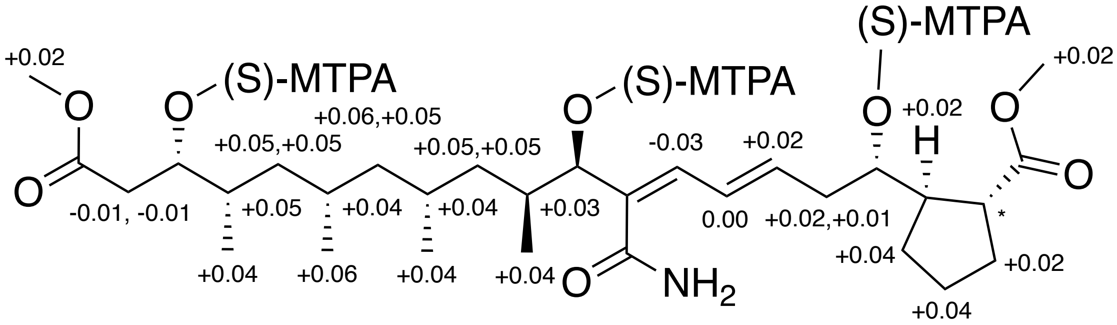

Position	(7) δ_H	(8) δ_H^{T1} (25 °C)	(8) δ_H^{T2} (5 °C)	(8) $\Delta\delta_H^{T1,T2} = \delta_H^{T1} - \delta_H^{T2}$
1	N/A			
2	2.68, 2.82	2.81, 2.88	2.82, 2.89	-0.01, -0.01
3	4.45	C**H**-O-(S)-MTPA		
4	1.81	2.08	2.05	+0.05
5	1.09, 1.71	1.06, 1.50	1.01, 1.45	+0.05, +0.05
6	0.89	1.77	1.75	+0.04
7	0.92, 1.26	0.97, 1.23	0.91, 1.18	+0.06, +0.05
8	1.79	1.69	1.65	+0.04
9	1.45, 1.47	1.17, 1.39	1.12, 1.34	+0.05, +0.05
10	2.35	2.31	2.28	+0.03
11	4.63	C**H**-O-(S)-MTPA		
12	N/A			
13	6.73	7.15	7.18	-0.03
14	7.39	6.75	6.75	0.00
15	6.26	6.20	6.18	+0.02
16	2.47, 2.52	2.63, 2.73	2.61, 2.72	+0.02, +0.01
17	3.71	C**H**-O-(S)-MTPA		
18	2.64	2.87	2.85	+0.02
19	1.42, 1.78	1.87	1.83	+0.04
20	1.63	1.57	1.53	+0.04
21	1.89, 1.95	1.37	1.35	+0.02
22	3.07	*	*	*
23	N/A			
24	1.06	0.99	0.95	+0.04
25	0.93	0.91	0.85	+0.06
26	0.92	0.93	0.89	+0.04
27	1.26	1.09	1.05	+0.04
28	N/A			
29	3.63	3.62	3.60	+0.02
30	3.67	3.68	3.66	+0.02

* not observed.

Supplementary References

1. Latypov, S. K., Seco, J. M., Quinoa, E. & Riguera, R. Are both the (R)- and the (S)-MPA esters really needed for the assignment of the absolute configuration of secondary alcohols by NMR? The use of a single derivative. *J. Am. Chem. Soc.* **120**, 877–882 (1998).
