# Supplementary material for: Collateral sensitivity profiling in drug-resistant Escherichia coli identifies natural products suppressing cephalosporin resistance
Source: Nat Commun. 2023 Apr 8;14:1976. doi: 10.1038/s41467-023-37624-4 (PMC10082850; doi:10.1038/s41467-023-37624-4)
Supplement: Supplementary file 3 — Reporting Summary [file 41467_2023_37624_MOESM3_ESM.pdf]

nature portfolio

Corresponding author(s): Linington, RG

Last updated by author(s): Mar 17, 2023

Reporting Summary

Nature Portfolio wishes to improve the reproducibility of the work that we publish. This form provides structure for consistency and transparency in reporting. For further information on Nature Portfolio policies, see our Editorial Policies and the Editorial Policy Checklist.

Statistics

For all statistical analyses, confirm that the following items are present in the figure legend, table legend, main text, or Methods section.

n/a	Confirmed	
☐	☒	The exact sample size (*n*) for each experimental group/condition, given as a discrete number and unit of measurement
☐	☒	A statement on whether measurements were taken from distinct samples or whether the same sample was measured repeatedly
☐	☒	The statistical test(s) used AND whether they are one- or two-sided<br>*Only common tests should be described solely by name; describe more complex techniques in the Methods section.*
☒	☐	A description of all covariates tested
☐	☒	A description of any assumptions or corrections, such as tests of normality and adjustment for multiple comparisons
☐	☒	A full description of the statistical parameters including central tendency (e.g. means) or other basic estimates (e.g. regression coefficient) AND variation (e.g. standard deviation) or associated estimates of uncertainty (e.g. confidence intervals)
☐	☒	For null hypothesis testing, the test statistic (e.g. *F*, *t*, *r*) with confidence intervals, effect sizes, degrees of freedom and *P* value noted<br>*Give P values as exact values whenever suitable.*
☒	☐	For Bayesian analysis, information on the choice of priors and Markov chain Monte Carlo settings
☒	☐	For hierarchical and complex designs, identification of the appropriate level for tests and full reporting of outcomes
☒	☐	Estimates of effect sizes (e.g. Cohen's *d*, Pearson's *r*), indicating how they were calculated

Our web collection on statistics for biologists contains articles on many of the points above.

Software and code

Policy information about availability of computer code

Data collection	Absorbance values were acquired on either a BioTek Synergy Neo2 plate reader running GEN5 software (v. 3.08) or a Molecular Devices SpectraMaxi3x running Softmax Pro software (v. 7.0.3). In both cases data were exported as flat csv files. NMR data were acquired using Bruker Topspin (v.3.5 PL6). Mass spectrometry data were acquired using Waters MassLynx (v. 4.1). HPLC data were acquired using Agilent ChemStation (v. B.04.02). Quantitative mass spectrometry data were acquired using AB Sciex Analyst (v1.6.2).
Data analysis	Sequence data were trimmed using Trimmomatic (v.0.35), assembled using Bowtie2 (v. 2.5.1) and mutation effects predicted using SnpEff (v. 4.5). Raw absorbance values from each plate were concatenated into a single file and standardized and normalized using Excel (Mac v16.65) as described in the methods section. Hierarchical clustering was performed using the open-source software package Morpheus (v. 1.0). Curve fitting and plot generation were performed in Graphpad Prism (v8). NMR data were processed using MNova (v14.1.0-24037). Mass spectrometry data were processed using Waters MassLynx (v. 4.1) or Waters UNIFI software (v.). Quantitative mass spectrometry data were analyzed using Skyline (v21.1.0.146).

For manuscripts utilizing custom algorithms or software that are central to the research but not yet described in published literature, software must be made available to editors and reviewers. We strongly encourage code deposition in a community repository (e.g. GitHub). See the Nature Portfolio guidelines for submitting code & software for further information.

Data

Policy information about availability of data

All manuscripts must include a data availability statement. This statement should provide the following information, where applicable:

- Accession codes, unique identifiers, or web links for publicly available datasets
- A description of any restrictions on data availability
- For clinical datasets or third party data, please ensure that the statement adheres to our policy

The Genome sequence data for all drug resistant E. coli mutants generated in this study have been deposited in the NCBI database under accession code PRJNA932790 [https://www.ncbi.nlm.nih.gov/bioproject/932790]. The NMR data for compounds 1-4 have been deposited in the Natural Products Magnetic Resonance Database under accession numbers NP0331529, NP0017652, NP0017654, NP0331530 [https://np-mrd.org/natural_products/NP0331529, https://np-mrd.org/natural_products/NP0017652, https://np-mrd.org/natural_products/NP0017654, https://np-mrd.org/natural_products/NP0331530]. The raw data from primary and secondary screens, as well as checkerboard assays and resistance passaging have been deposited in Zenodo [https://doi.org/10.5281/zenodo.7552218]. Source data are provided with this paper.

Human research participants

Policy information about studies involving human research participants and Sex and Gender in Research.

Reporting on sex and gender	N/A
Population characteristics	N/A
Recruitment	N/A
Ethics oversight	N/A

Note that full information on the approval of the study protocol must also be provided in the manuscript.

Field-specific reporting

Please select the one below that is the best fit for your research. If you are not sure, read the appropriate sections before making your selection.

☒ Life sciences ☐ Behavioural & social sciences ☐ Ecological, evolutionary & environmental sciences

For a reference copy of the document with all sections, see nature.com/documents/nr-reporting-summary-flat.pdf

Life sciences study design

All studies must disclose on these points even when the disclosure is negative.

Sample size	Antibiotic control library size (80 compounds) was determined by commercial availability of reference material and supply costs. Most compound classes include a minimum of three members. Natural product library size (6,195 prefractions) include the full set of available prefractions in our in-house library which has been developed over a 15 year period. No prefractions were excluded from this study.
Data exclusions	No data were exluded from either primary or secondary screening. In some cases, initial screening experiments failed quality control (QC) metrics. In all cases these experiments were repeated to obtain data that met QC requirements (Z' factor > 0.5).
Replication	All primary and secondary screening was performed in triplicate except for the primary natural product (NP) screen that was performed as a single replicate due to material limitations with the NP library. For intracellular accumulation experiments five replicates (n = 5) were prepared for each bacterial strain per drug condition (no drug, 4 µM 1, and 4 µM mupirocin). One replicate was lost for the Cef6 intracellular borrelidin treatment (n = 4).
Randomization	NP prefractions are arrayed randomly in screening pates, without pre-organization by taxonomic origin or other groupings.
Blinding	The chemical composition of prefractions is not known during the primary or secondary screening stages, meaning that the NP library is blinded with respect to molecular composition.

Reporting for specific materials, systems and methods

We require information from authors about some types of materials, experimental systems and methods used in many studies. Here, indicate whether each material, system or method listed is relevant to your study. If you are not sure if a list item applies to your research, read the appropriate section before selecting a response.

Materials & experimental systems

n/a	Involved in the study
☒	☐ Antibodies
☒	☐ Eukaryotic cell lines
☒	☐ Palaeontology and archaeology
☒	☐ Animals and other organisms
☒	☐ Clinical data
☒	☐ Dual use research of concern

Methods

n/a	Involved in the study
☒	☐ ChIP-seq
☒	☐ Flow cytometry
☒	☐ MRI-based neuroimaging
